# Supplementary material for: Does weight management research for adults with severe obesity represent them? Analysis of systematic review data
Source: BMJ Open. 2022 May 30;12(5):e054459. doi: 10.1136/bmjopen-2021-054459 (PMC9157335; doi:10.1136/bmjopen-2021-054459)
Supplement: Supplementary data [file bmjopen-2021-054459supp001.pdf]

Appendix 1 Literature search strategies

Literature searching was conducted in June 2016 and updated in April/May 2017. The following databases were searched without language restriction: MEDLINE, MEDLINE Epub Ahead of Print & MEDLINE In-Process, PsychINFO, Cumulative Index to Nursing and Allied Health Literature (CINAHL), Science Citation Index (SCI), Cochrane Central Register of Controlled Trials (CENTRAL), and ClincalTrials.gov. Ovid MEDLINE and EMBASE autoalerts have been in place since 2002 and results are screened regularly for long-term RCTs on obesity management in adults (≥ 1 year of follow-up). Copies of relevant reports are retained and the reviewers hand-searched these reports eligible studies. Therefore, search strategies for MEDLINE and EMBASE excluded the results of the autoalert search (using the Boolean operator NOT). A supplementary search of MEDLINE was undertaken to identify systematic reviews of severe or morbid obesity, and reference lists were scrutinised for additional studies.

RCTs for Review 1 (weight loss or weight maintenance programmes for adults with obesity and Review 5 (weight loss or weight maintenance programmes for adults with obesity compared with bariatric surgery)
MEDLINE and EMBASE
Ovid multifile search: http://shibboleth.ovid.com/
Database: Embase <1988 to 2017 Week 17>, Ovid MEDLINE(R) Epub Ahead of Print, In-Process & Other Non-Indexed Citations, Ovid MEDLINE(R) Daily and Ovid MEDLINE(R) <1946 to Present> 25th April 2017
Date of Search: 25th April 2017
1 Obesity, Morbid/dh, dt, su, th use ppez
2 Morbid Obesity/dt, su, th use emed
3 Obesity, Morbid/ use ppez
4 Morbid Obesity/ use emed
5 (obes$ adj3 (morbid$ or severe$)).tw.
6 (morbid obesity or severe obesity).kw.
7 (bmi adj ("27$" or "28$" or "29$")).tw.
8 (bmi adj ("30$" or "31$" or "32$" or "33$" or "34$" or "35$" or "36$" or "37$" or "38$" or "39$")).tw.
9 (bmi adj ("40$" or "41$" or "42$" or "43$" or "44$" or "45$" or "46$" or "47$" or "48$" or "49$")).tw.
10 (bmi adj ("50$" or "51$" or "52$" or "53$" or "54$" or "55$")).tw.
11 (body mass index adj ("35$" or "36$" or "37$" or "38$" or "39$")).tw.
12 (body mass index adj ("40$" or "41$" or "42$" or "43$" or "44$" or "45$" or "46$" or "47$" or "48$" or "49$")).tw.
13 (body mass index adj ("50$" or "51$" or "52$" or "53$" or "54$" or "55$")).tw.
14 or/3-12
15 exp bariatric surgery/
16 bariatric.tw,kw.

17 (gastric adj3 (bypass or bypass or band$ or balloon$)).tw.
18 (gastroplast$ or gastrect$).tw.
19 (jejunoileal adj3 (bypass or by pass)).tw.
20 lipectom$.tw
21 diet therapy/ use ppez or caloric restriction/ use ppez or diet, carbohydrate-restricted/ use ppez or diet, fat-restricted/ use ppez or diet, reducing/ use ppez
22 diet/ use emed or low calory diet/ use emed or low carbohydrate diet/ use emed
23 (diet adj3 (restrict$ or reduc$ or modif$ or calorie$)).tw.
24 (calori$ adj3 (reduc$ or restrict$ or limit$)).tw.
25 exp anti-obesity agents/ use ppez
26 tetrahydrolipstatin/ use emed
27 sibutramine/ use emed
28 rimonabant/ use emed
29 (appetite adj3 (reduc$ or depress$)).tw.
30 (orlistat or xenical).tw,rn.
31 (sibutramin$ or arcalion or reductil or medaria or meridia).tw,rn.
32 (rimonabant or acomplia or zimulti).tw,rn.
33 exp exercise/
34 exp sports/
35 (exercise or aerobic or physical activ$).tw.
36 weight loss/ use ppez
37 weight reduction/ use emed
38 (weight adj3 (reduc$ or loss or lose)).tw.
39 Weight Reduction Programs/ use ppez
40 exp Body Weight Management/ use emed
41 Behavior Therapy/
42 Health Education/
43 Counseling/ use ppez
44 Nutritional counseling/ use emed
45 Life Style/ use ppez
46 Lifestyle modification/ use emed
47 (modif$ adj3 (behavio?r or diet or eat? or eating)).tw.
48 ((diet or weight) adj3 (educat$ or counsel$)).tw.

49 or/15-46
50 1 or 2 or (14 and 49)
51 randomized controlled trial.pt.
52 controlled clinical trial.pt
53 randomi?ed.ab.
54 placebo.ab.
55 drug therapy.fs.
56 randomly.ab.
57 trial.ab.
58 groups.ab
59 nonhuman/ not human/
60 exp clinical trial/ use emed
61 randomization/ use emed
62 51 or 52 or 53 or 54 or 55 or 56 or 57 or 58 or 60 or 61
63 exp animals/ use ppez not humans/ use ppez
64 nonhuman/ use emed or animal/ use emed
65 64 not exp human/ use emed
66 62 not (63 or 65)
67 50 and 66
68 67 not (abstract or letter or note or editorial or comment).pt.
69 limit 68 to yr="1990 -Current"
70 obesity/
71 (obesity adj2 (morbid or diabet$)).tw.
72 obesity, morbid/ use ppez
73 morbid obesity/ use emed
74 obes$.tw.
75 weight loss/ use ppez
76 weight reduction/ use emed
77 (weight adj1 (los$ or reduc$ or maint$ or control)).tw.
78 (diet adj5 weight).tw.
79 overweight.tw.
80 or/70-79
81 80 and 66

82 69 not 81

PsycINFO

Ovid: http://shibboleth.ovid.com/

Database: PsycINFO <1987 to April Week 3 2017>

Date of Search: 25th April 2017

1 (obes$ adj3 (morbid$ or severe$)).tw.
2 (bmi adj ("27$" or "28$" or "29$")).tw.
3 (bmi adj ("30$" or "31$" or "32$" or "33$" or "34$" or "35$" or "36$" or "37$" or "38$" or "39$")).tw.
4 (bmi adj ("40$" or "41$" or "42$" or "43$" or "44$" or "45$" or "46$" or "47$" or "48$" or "49$")).tw.
5 (bmi adj ("50$" or "51$" or "52$" or "53$" or "54$" or "55$")).tw. (
6 (body mass index adj ("27$" or "28$" or "29$")).tw.
7 (body mass index adj ("30$" or "31$" or "32$" or "33$" or "34$" or "35$" or "36$" or "37$" or "38$" or "39$")).tw.
8 (body mass index adj ("40$" or "41$" or "42$" or "43$" or "44$" or "45$" or "46$" or "47$" or "48$" or "49$")).tw.
9 (body mass index adj ("50$" or "51$" or "52$" or "53$" or "54$" or "55$")).tw. (7)
10 or/1-9
11 clinical trials/
12 randomi?ed.tw,md.
13 randomly.tw,md.
14 trial?.tw,md
15 or/11-14
16 10 and 15

CINAHL

http://search.ebscohost.com

!990- 25th April 2017

Date of Search: 25th April 2017

S1 (MH "Obesity, Morbid") OR TX (obes|* N3 (morbid or sever*))
S2 TX (bmi N3 (27 or 28 or 29 or 30 or 31 or 32 or 33 or 34 or 35 or 36 or 37 or 38 or 39).) OR TX (bmi N3 (40 or 41 or 42 or 43 or 44 or 45 or 46 or 47 or 48 or 49).) OR TX (bmi N3 (50 or 51 or 52 or 53 or 54 or 55).)
S3 TX (body mass index N3 (35 or 36 or 37 or 38 or 39).) OR TX (body mass index N3 (40 or 41 or 42 or 43 or 44 or 45 or 46 or 47 or 48 or 49).) OR TX (body mass index N3 (50 or 51 or 52 or 53 or 54 or 55).)
S4 S1 OR S2 OR S3
S5 (MH "Bariatric Surgery+")
S6 TX bariatric OR TX gastroplasty
S7 TX bariatric OR TX gastroplast* AND TX gastrect*
S8 TX (gastric N3 (by pass or by pass or band* or balloon*)) OR TX (jejunoileal N3 (bypass or by pass) OR TX lipectom*
S9 (MH "Diet Therapy") OR (MH "Diet, Reducing") OR (MH "Restricted Diet") OR (MH "Diet, Fat-Restricted") OR (MH "Diet, Low Carbohydrate")
S10 TX (diet N3 (restrict* or reduc* or modif* or calorie*))) OR TX (calori* N3 (reduc* or restrict* or limit*)).)
S11 (MH "Antiobesity Agents+")
S12 TX (orlistat or xenical) OR TX (sibutramin$ or arcalion or reductil or medaria or meridia) OR TX (rimonabant or acomplia or zimulti)
S13 (MH "Exercise+")
S14 TX exercise or aerobic or physical activ*).
S15 (MH "Physical Fitness")
S16 (MH "Weight Loss")
S17 TX weight N3 (reduc* or loss or lose)).
S18 (MH "Weight Reduction Programs")
S19 (MH "Counseling")
S20 (MH "Life Style") OR (MH "Life Style Changes")
S21 TX (modif* N3 (behavior or behaviour or diet or eat* or eating) OR TX ((diet or weight) N3 (educat* or counsel*)).)

S22 S5 OR S6 OR S7 OR S8 OR S9 OR S10 OR S11 OR S12 OR S13 OR S14 OR S15 OR S16 OR S17 OR S18 OR S19 OR S21
S23 S4 AND S22 Limiters - Published Date: 19900101-20161231
S24 S4 AND S22 Limiters - Exclude MEDLINE records
Science Citation Index
www.webofknowledge.com
1988 - 25th April 2017
Date of Search: 25th April 2017
1 (TS=(morbid NEAR/3 obes$)) AND DOCUMENT TYPES: (Article)
2 (TS=(severe$ NEAR/3 obes$)) AND DOCUMENT TYPES: (Article)
3 (TS=(bmi NEAR/3 (27 or 28 or 29 or 30 or 31 or 32 or 33 or 34 or 35 or 36 or 37 or 38 or 39))) AND DOCUMENT TYPES: (Article)
4 ((TS=(bmi NEAR/3 (40 or 41 or 42 or 43 or 44 or 45 or 46 or 47 or 48 or 49)))) AND DOCUMENT TYPES: (Article)
5 ((TS=(bmi NEAR/3 (50 or 51 or 52 or 53 or 54 or 55)))) AND DOCUMENT TYPES: (Article)
6 #5 OR #4 OR #3 OR #2 OR #1
7 (TS=(randomized or randomised or randomly)) AND DOCUMENT TYPES: (Article)
8 (TS=clinical trial*) AND DOCUMENT TYPES: (Article)
9 #8 OR #7
10 #9 AND #6
11 (TS=(bariatric or gastroplast* or gastrect*)) AND DOCUMENT TYPES: (Article)
12 (TS=((gastric or jejunoileal) NEAR/3 bypass)) AND DOCUMENT TYPES: (Article)
13 ((TS=((gastric or jejunoileal) NEAR/3 "by pass"))) AND DOCUMENT TYPES: (Article)
14 (TS=(gastric NEAR/3 (band* or balloon*))) AND DOCUMENT TYPES: (Article)

15 (TS=(diet NEAR/3 (restrict* or reduc* or modif* or calorie*)).) AND DOCUMENT TYPES: (Article)
16 (TS=(calori* NEAR/3 (reduc* or restrict* or limit*)).) AND DOCUMENT TYPES: (Article)
17 (TS=(appetite NEAR/3 (reduc* or depress*)).) AND DOCUMENT TYPES: (Article)
18 (TS=(orlistat or xenical).) AND DOCUMENT TYPES: (Article)
19 (TS=(sibutramin or arcalion or reductil or medaria or meridia).) AND DOCUMENT TYPES: (Article)
20 (TS=(rimonabant or acomplia or zimulti).) AND DOCUMENT TYPES: (Article)
21 (TS=(exercise or aerobic or physical active*).) AND DOCUMENT TYPES: (Article)
22 (TS=(weight NEAR/3 (reduc* or loss or lose))) AND DOCUMENT TYPES: (Article)
23 (TS=(modif* NEAR/3 (behavior or behaviour or diet or eat? or eating)).) AND DOCUMENT TYPES: (Article)
24 (TS=(diet NEAR/3 (educat* or counsel*)).) AND DOCUMENT TYPES: (Article)
25 (TS=(weight NEAR/3 (educat* or counsel*))) AND DOCUMENT TYPES: (Article)
26 #25 OR #24 OR #23 OR #22 OR #21 OR #20 OR #19 OR #18 OR #17 OR #16 OR #15 OR #14 OR #13 OR #12 OR #11
#27 #10 AND #26
Cochrane Central Register of Controlled Trials
www.thecochranelibrary.com
Issue 4 April 2017
Date of Search: 25th April 2017
#1 MeSH descriptor: [Obesity, Morbid] explode all trees and with qualifier(s): [Diet therapy - DH, Surgery - SU, Therapy - TH]

#2 MeSH descriptor: [Obesity, Morbid] explode all trees
#3 obes* near/3 (morbid* or severe*):ti,ab,kw (Word variations have been searched)
#4 bmi near/1 (27* or 28* or 29* or 30* or 31* or 32* or 33* or 34* or 35* or 36* or 37* or 38* or 39*):ti,ab,kw or bmi near/1 (40* or 41* or 42* or 43* or 44* or 45* or 46* or 47* or 48* or 49*):ti,ab,kw or bmi near/1 (50* or 51* or 52* or 53* or 54* or 55*):ti,ab,kw (Word variations have been searched)
#5 body mass index near/1 (27* or 28* or 29* or 30* or 31* or 32* or 33* or 34* or 35* or 36* or 37* or 38* or 39*):ti,ab,kw or body mass index near/1 (40* or 41* or 42* or 43* or 44* or 45* or 46* or 47* or 48* or 49*):ti,ab,kw or body mass index near/1 (50* or 51* or 52* or 53* or 54* or 55*):ti,ab,kw (Word variations have been searched)
#6 #2 or #3 or #4 or #5
#7 MeSH descriptor: [Bariatric Surgery] explode all trees
#8 bariatric:ti,ab,kw (Word variations have been searched)
#9 gastric near/3 (bypass or by pass or band* or balloon*):ti,ab,kw or gastroplast* or gastrect*:ti,ab,kw or lipectom*:ti,ab,kw or jejunoileal near/3 (bypass or by pass):ti,ab,kw (Word variations have been searched)
#10 MeSH descriptor: [Diet Therapy] explode all trees
#11 MeSH descriptor: [Diet, Reducing] explode all trees
#12 MeSH descriptor: [Diet, Fat-Restricted] explode all trees
#13 MeSH descriptor: [Diet, Carbohydrate-Restricted] explode all trees
#14 MeSH descriptor: [Caloric Restriction] explode all trees
#15 diet near/3 (restrict* or reduc* or modif* or calorie*):ti,ab,kw or calori* near/3 (reduc* or restrict* or limit*):ti,ab,kw (Word variations have been searched)
#16 MeSH descriptor: [Anti-Obesity Agents] explode all trees
#17 appetite near/3 (reduc* or depress*):ti,ab,kw or orlistat or xenical:ti,ab,kw or sibutramin or arcalion or reductil or medaria or meridia:ti,ab,kw or rimonabant or acomplia or zimulti:ti,ab,kw (Word variations have been searched)
#18 MeSH descriptor: [Exercise] explode all trees
#19 MeSH descriptor: [Sports] explode all trees
#20 exercise or aerobic* or physical activ*:ti,ab,kw (Word variations have been searched)

#21 MeSH descriptor: [Weight Loss] explode all trees
#22 MeSH descriptor: [Weight Reduction Programs] explode all trees
#23 weight near/3 (reduc* or loss or lose) .:ti,ab,kw (Word variations have been searched)
#24 weight near/3 (reduc* or loss or lose) .:ti,ab,kw (Word variations have been searched)
#25 MeSH descriptor: [Behavior Therapy] explode all trees
#26 MeSH descriptor: [Health Education] explode all trees
#27 MeSH descriptor: [Counseling] explode all trees
#28 MeSH descriptor: [Life Style] explode all trees
#29 diet near/3 (educat* or counsel*):ti,ab,kw or modif* near/3 (behaviour or behavior or diet or eat or eating):ti,ab,kw or weight near/3 (educat* or counsel*):ti,ab,kw (Word variations have been searched)
#30 #7 or #8 or #9 or #10 or #11 or #12 or #13 or #14 or #15 or #16 or #17 or #18 or #19 or #20 or #21 or #22 or #23 or #24 or #25 or #26 or #27 or #28 or #29
#31 #6 and #30
#32 #1 or #31 Publication Year from 1990 to 2016, in Trials
#33 conference abstract (Word variations have been searched)
#34 #32 not #33

Appendix 2 PRISMA flow diagram

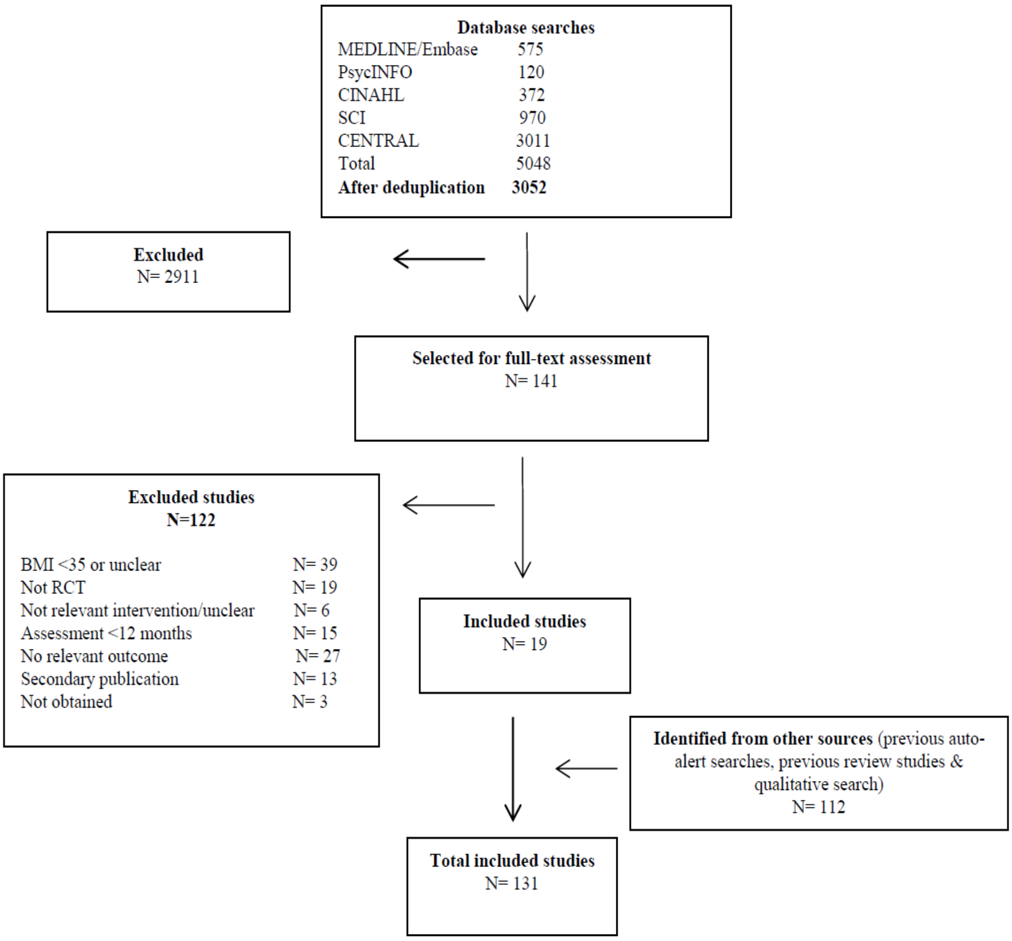

Appendix 3 List of included studies

Agras WS, Berkowitz RI, Arnow BA, Telch CF, Marnell M, Henderson J, et al. Maintenance following a very-low-calorie diet. *Journal of Consulting & Clinical Psychology* 1996;**64:**610-3.

Annesi JJ. Mediation of the relationship of behavioural treatment type and changes in psychological predictors of healthy eating by body satisfaction changes in women with obesity. *Obesity Research & Clinical Practice* 2017;**11:**97-107.

Appel LJ, Clark JM, Yeh HC, Wang NY, Coughlin JW, Daumit G, et al. Comparative effectiveness of weight-loss interventions in clinical practice. *New England Journal of Medicine* 2011;**365:**1959-68.

Azar KM, Xiao L, Ma J. Baseline obesity status modifies effectiveness of adapted diabetes prevention program lifestyle interventions for weight management in primary care. *BioMed Research International* 2013;191209.

Bacon L, Keim NL, Van Loan MD, Derricote M, Gale B, Kazaks A, et al. Evaluating a 'non-diet' wellness intervention for improvement of metabolic fitness, psychological well-being and eating and activity behaviors. *International Journal of Obesity & Related Metabolic Disorders* 2002;**26:**854-65.

Bakris G, Calhoun D, Egan B, Hellmann C, Dolker M, Kingma I. Orlistat improves blood pressure control in obese subjects with treated but inadequately controlled hypertension. *Journal of Hypertension* 2002;**20:**2257-67.

Bartels SJ, Pratt SI, Aschbrenner KA, Barre LK, Jue K, Wolfe RS, et al. Clinically Significant Improved Fitness and Weight Loss Among Overweight Persons With Serious Mental Illness. *Psychiatric Services* 2013;**64:**729-36.

Bartels SJ, Pratt SI, Aschbrenner KA, Barre LK, Naslund JA, Wolfe R, et al. Pragmatic Replication Trial of Health Promotion Coaching for Obesity in Serious Mental Illness and Maintenance of Outcomes. *American Journal of Psychiatry* 2015;**172:**344-52.

Bazzano LA, Hu T, Reynolds K, Yao L, Bunol C, Liu Y, et al. Effects of low-carbohydrate and low-fat diets: a randomized trial. *Annals of Internal Medicine* 2014;**161:**309-18.

Bennett GG, Warner ET, Glasgow RE, Askew S, Goldman J, Ritzwoller DP, et al. Obesity treatment for socioeconomically disadvantaged patients in primary care practice. *Archives of Internal Medicine* 2012;**172:**565-74.

Berry DC, Schwartz TA, McMurray RG, Skelly AH, Neal M, Hall EG, et al. The family partners for health study: a cluster randomized controlled trial for child and parent weight management. *Nutrition & Diabetes* 2014;**4:**e101.

Berteus Forslund H, Klingstrom S, Hagberg H, Londahl M, Torgerson JS, Lindroos AK. Should snacks be recommended in obesity treatment? A 1-year randomized clinical trial. *European Journal of Clinical Nutrition* 2008;**62:**1308-17.

Beutel ME, Dippel A, Szczepanski M, Thiede R, Wiltink J. Mid-term effectiveness of behavioral and psychodynamic inpatient treatments of severe obesity based on a randomized study. *Psychotherapy and Psychosomatics* 2006;**75:**337-45.

Bliddal H, Leeds AR, Stigsgaard L, Astrup A, Christensen R. Weight loss as treatment for knee osteoarthritis symptoms in obese patients: 1-Year results from a randomised controlled trial. *Annals of the Rheumatic Diseases* 2011;**70:**1798-803.

Brehm BJ, Lattin BL, Summer SS, Boback JA, Gilchrist GM, Jandacek RJ, et al. One-year comparison of a high-monounsaturated fat diet with a high-carbohydrate diet in type 2 diabetes. *Diabetes Care* 2009;**32:**215-20.

Broom I, Hughes E, Dodson P, Reckless J. The role of orlistat in the treatment of obese patients with mild to moderate hypercholesterolaemia: Consequences for coronary risk. *British Journal of Cardiology* 2002;**9:**460-1+8.

Broom I, Wilding J, Stott P, Myers N. Randomised trial of the effect of orlistat on body weight and cardiovascular disease risk profile in obese patiensts: UK multimorbidity study. *International Journal of Clinical Practice* 2002;**56:**494-9.

Burguera B, Jesus Tur J, Escudero AJ, Alos M, Pagan A, Cortes B, et al. An Intensive Lifestyle Intervention Is an Effective Treatment of Morbid Obesity: The TRAMOMTANA Study-A Two-Year Randomized Controlled Clinical Trial. *International journal of endocrinology* 2015.

Cheskin LJ, Mitchell AM, Jhaveri AD, Mitola AH, Davis LM, Lewis RA, et al. Efficacy of meal replacements versus a standard food-based diet for weight loss in type 2 diabetes: a controlled clinical trial. *Diabetes Educator* 2008;**34:**118-27.

Christensen P, Frederiksen R, Bliddal H, Riecke BF, Bartels EM, Henriksen M, et al. Comparison of three weight maintenance programs on cardiovascular risk, bone and vitamins in sedentary older adults. *Obesity* 2013;**21:**1982-90.

Courcoulas AP, Goodpaster BH, Eagleton JK, Belle SH, Kalarchian MA, Lang W, et al. Surgical vs medical treatments for type 2 diabetes mellitus: a randomized clinical trial. *JAMA Surgery* 2014;**149:**707-15.

Cummings DE, Arterburn DE, Westbrook EO, Kuzma JN, Stewart SD, Chan CP, et al. Gastric bypass surgery vs intensive lifestyle and medical intervention for type 2 diabetes: the CROSSROADS randomised controlled trial. *Diabetologia* 2016;**59:**945-53.

Dalle Grave R, Calugi S, Gavasso I, El Ghoch M, Marchesini G. A randomized trial of energy-restricted high-protein versus high-carbohydrate, low-fat diet in morbid obesity. *Obesity* 2013;**21:**1774-81.

Damschroder LJ, Lutes LD, Kirsh S, Kim HM, Gillon L, Holleman RG, et al. Small-changes obesity treatment among veterans: 12-month outcomes. *American Journal of Preventive Medicine* 2014;**47:**541-53.

Daubenmier J, Moran PJ, Kristeller J, Acree M, Bacchetti P, Kemeny ME, et al. Effects of a mndfulness-based weight loss intervention in adults with obesity: A randomized clinical trial. *Obesity* 2016;**24:**794-804.

Daumit GL, Dickerson FB, Wang NY, Dalcin A, Jerome GJ, Anderson CAM, et al. A behavioral weight-loss intervention in persons with serious mental illness. *New England Journal of Medicine* 2013;**368:**1594-602.

Davidson MH, Hauptman J, DiGirolamo M, Foreyt JP, Halsted CH, Heber D, et al. Weight control and risk factor reduction in obese subjects treated for 2 years with orlistat: A randomized controlled trial. *Journal of the American Medical Association* 1999;**281:**235-42.

Davis NJ, Tomuta N, Schechter C, Isasi CR, Segal-Isaacson CJ, Stein D, et al. Comparative study of the effects of a 1-year dietary intervention of a low-carbohydrate diet versus a low-fat diet on weight and glycemic control in type 2 diabetes. *Diabetes Care* 2009;**32:**1147-52.

Delbridge EA, Prendergast LA, Pritchard JE, Proietto J. One-year weight maintenance after significant weight loss in healthy overweight and obese subjects: Does diet composition matter? *American Journal of Clinical Nutrition* 2009;**90:**1203-14.

Dennison KF, Galante D, Dennison D, Golaszewski T. A one year post-program assessment of a computer-assisted instruction weight management program for industrial employees: lessons learned. *Journal of Health Education* 1996;**27:**38-43.

Dixon JB, O'Brien PE, Playfair J, Chapman L, Schachter LM, Skinner S, et al. Adjustable gastric banding and conventional therapy for type 2 diabetes: a randomized controlled trial. *JAMA* 2008;**299:**316-23.

Dixon JB, Schachter LM, O'Brien PE, Jones K, Grima M, Lambert G, et al. Surgical vs conventional therapy for weight loss treatment of obstructive sleep apnea: A randomized controlled trial. *JAMA* 2012;**308:**1142-9.

Djuric Z, Mirasolo J, Kimbrough L, Brown DR, Heilbrun LK, Canar L, et al. A pilot trial of spirituality counseling for weight loss maintenance in African American breast cancer survivors. *Journal of the National Medical Association* 2009;**101:**552-64.

Dutton GR, Nackers LM, Dubyak PJ, Rushing NC, Huynh TVT, Tan F, et al. A randomized trial comparing weight loss treatment delivered in large versus small groups. *International Journal of Behavioral Nutrition and Physical Activity* 2014;**11 (1)**

Eaton CB, Hartman SJ, Perzanowski E, Pan GH, Roberts MB, Risica PM, et al. A Randomized Clinical Trial of a Tailored Lifestyle Intervention for Obese, Sedentary, Primary Care Patients. *Annals of Family Medicine* 2016;**14:**311-9.

Ebbeling CB, Leidig MM, Feldman HA, Lovesky MM, Ludwig DS. Effects of a low-glycemic load vs low-fat diet in obese young adults: A randomized trial. *Journal of the American Medical Association* 2007;**297:**2092-102.

Esposito K, Giugliano F, Di Palo C, Giugliano G, Marfella R, D'Andrea F, et al. Effect of lifestyle changes on erectile dysfunction in obese men: A randomized controlled trial. *Journal of the American Medical Association* 2004;**291:**2978-84.

Finer N, James WPT, Kopelman PG, Lean MEJ, Williams G. One-year treatment of obesity: A randomized, double-blind, placebo-controlled, multicentre study of orlistat, a gastrointestinal lipase inhibitor. *International Journal of Obesity* 2000;**24:**306-13.

Fitzgibbon ML, Stolley MR, Schiffer L, Sharp LK, Singh V, Dyer A. Obesity reduction black intervention trial (ORBIT): 18-month results. *Obesity* 2010;**18:**2317-25.

Flechtner-Mors M, Boehm BO, Wittmann R, Thoma U, Ditschuneit HH. Enhanced weight loss with protein-enriched meal replacements in subjects with the metabolic syndrome. *Diabetes/Metabolism Research and Reviews* 2010;**26:**393-405.

Foster GD, Wyatt HR, Hill JO, Makris AP, Rosenbaum DL, Brill C, et al. Weight and metabolic outcomes after 2 years on a low-carbohydrate versus low-fat diet: A randomized trial. *Annals of Internal Medicine* 2010;**153:**147-57.

Goodpaster BH, Delany JP, Otto AD, Kuller L, Vockley J, South-Paul JE, et al. Effects of diet and physical activity interventions on weight loss and cardiometabolic risk factors in severely obese adults: a randomized trial. *JAMA* 2010;**304:**1795-802.

Gorin AA, Raynor HA, Fava J, Maguire K, Robichaud E, Trautvetter J, et al. Randomized controlled trial of a comprehensive home environment-focused weight-loss program for adults. *Health Psychology* 2013;**32:**128-37.

Green CA, Yarborough BJH, Leo MC, Stumbo SP, Perrin NA, Nichols GA, et al. Weight maintenance following the STRIDE lifestyle intervention for individuals taking antipsychotic medications. *Obesity* 2015;**23:**1995-2001.

Hakala P, Karvetti RL, Rönnemaa T. Group vs. individual weight reduction programmes in the treatment of severe obesity--a five year follow-up study. *International journal of obesity and related metabolic disorders* 1993;**17:**97-102.
Hakala P. Weight reduction programmes at a rehabilitation centre and a health centre based on group counselling and individual support: Short- and long-term follow-up study. *International Journal of Obesity* 1994;**18:**483-9.

Hauptman J, Lucas C, Boldrin MN, Collins H, Segal KR. Orlistat in the long-term treatment of obesity in primary care settings. *Archives of Family Medicine* 2000;**9:**160-7.

Hunt K, Wyke S, Gray CM, Anderson AS, Brady A, Bunn C, et al. A gender-sensitised weight loss and healthy living programme for overweight and obese men delivered by Scottish Premier League football clubs (FFIT): A pragmatic randomised controlled trial. *The Lancet* 2014;**383:**1211-21.

Iqbal N, Vetter ML, Moore RH, Chittams JL, Dalton-Bakes CV, Dowd M, et al. Effects of a low-intensity intervention that prescribed a low-carbohydrate vs. a low-fat diet in obese, diabetic participants. *Obesity* 2010;**18:**1733-8.

Jesudason DR, Pedersen E, Clifton PM. Weight-loss diets in people with type 2 diabetes and renal disease: a randomized controlled trial of the effect of different dietary protein amounts. *American Journal of Clinical Nutrition* 2013;**98:**494-501.

Kahleova H, Hill M, Pelikanova T. Vegetarian vs. conventional diabetic diet - A 1-year follow-up. *Cor et Vasa* 2014;**56:**e140-e4.

Kelley DE, Bray GA, Pi-Sunyer FX, Klein S, Hill J, Miles J, et al. Clinical efficacy of orlistat therapy in overweight and obese patients with insulin-treated type 2 diabetes: A 1-year randomized controlled trial. *Diabetes Care* 2002;**25:**1033-41.

Keranen AM, Savolainen MJ, Reponen AH, Kujari ML, Lindeman SM, Bloigu RS, et al. The effect of eating behavior on weight loss and maintenance during a lifestyle intervention. *Preventive Medicine* 2009;**49:**32-8.

Khoo J, Piantadosi C, Duncan R, Worthley SG, Jenkins A, Noakes M, et al. Comparing effects of a low-energy diet and a high-protein low-fat diet on sexual and endothelial function, urinary tract symptoms, and inflammation in obese diabetic men. *Journal of Sexual Medicine* 2011;**8:**2868-75.

Krebs JD, Elley CR, Parry-Strong A, Lunt H, Drury PL, Bell DA, et al. The Diabetes Excess Weight Loss (DEWL) Trial: A randomised controlled trial of high-protein versus high-carbohydrate diets over 2 years in type 2 diabetes. *Diabetologia* 2012;**55:**905-14.

Krempf M, Louvet JP, Allanic H, Miloradovich T, Joubert JM, Attali JR. Weight reduction and long-term maintenance after 18 months treatment with orlistat for obesity. *International Journal of Obesity* 2003;**27:**591-7.

Kumanyika SK, Fassbender JE, Sarwer DB, Phipps E, Allison KC, Localio R, et al. One-year results of the think health study of weight management in primary care practices. *Obesity* 2012;**20:**1249-57.

Kumanyika SK, Shults J, Fassbender J, Whitt MC, Brake V, Kallan MJ, et al. Outpatient weight management in African-Americans: The Healthy Eating and Lifestyle Program (HELP) study. *Preventive Medicine* 2005;**41:**488-502.

Kumanyika SK, Wadden TA, Shults J, Fassbender JE, Brown SD, Bowman MA, et al. Trial of family and friend support for weight loss in African American adults. *Archives of Internal Medicine* 2009;**169:**1795-804.

Lantz H, Peltonen M, Agren L, Torgerson JS. Intermittent versus on-demand use of a very low calorie diet: A randomized 2-year clinical trial. *Journal of Internal Medicine* 2003;**253:**463-71.

Latner JD, Ciao AC, Wendicke AU, Murakami JM, Durso LE. Community-based behavioral weight-loss treatment: Long-term maintenance of weight loss, physiological, and psychological outcomes. *Behaviour Research and Therapy* 2013;**51:**451-9.

Lillis J, Niemeier HM, Thomas JG, Unick J, Ross KM, Leahey TM, et al. A Randomized Trial of an Acceptance-Based Behavioral Intervention for Weight Loss in People with High Internal Disinhibition. *Obesity* 2016;**24:**2509-14.

Linde JA, Simon GE, Ludman EJ, Ichikawa LE, Operskalski BH, Arterburn D, et al. A randomized controlled trial of behavioral weight loss treatment versus combined weight loss/depression treatment among women with comorbid obesity and depression. *Annals of behavioral medicine* 2011;**41:**119-30.

Little P, Stuart B, Hobbs FR, Kelly J, Smith ER, Bradbury KJ, et al. An internet-based intervention with brief nurse support to manage obesity in primary care (POWeR+): a pragmatic, parallel-group, randomised controlled trial. *The Lancet Diabetes and Endocrinology* 2016;**4:**821-8.

Look Ahead Research Group, Wing RR, Bolin P, Brancati FL, Bray GA, Clark JM, et al. Cardiovascular effects of intensive lifestyle intervention in type 2 diabetes.[Erratum appears in N Engl J Med. 2014 May 8;370(19):1866]. *New England Journal of Medicine* 2013;**369:**145-54.

Lowe MR, Butryn ML, Thomas JG, Coletta M. Meal replacements, reduced energy density eating, and weight loss maintenance in primary care patients: a randomized controlled trial. *Obesity* 2014;**22:**94-100.

Ma J, Strub P, Xiao L, Lavori PW, Camargo CA, Jr., Wilson SR, et al. Behavioral weight loss and physical activity intervention in obese adults with asthma. A randomized trial. *Annals of the American Thoracic Society* 2015;**12:**1-11.

Maclaughlin HL, Hall WL, Patel AG, Blacklock RM, Swift PA, Phanish MK, et al. Weight loss, adipokines, and quality of life after sleeve gastrectomy in obese patients with stages 3-4 CKD: A randomized controlled pilot study. *American Journal of Kidney Diseases* 2014;**64:**660-3.

Manzoni GM, Cesa GL, Bacchetta M, Castelnuovo G, Conti S, Gaggioli A, et al. Virtual reality-enhanced cognitive-behavioral therapy for morbid obesity: A randomized controlled study with 1 year follow-up. *Cyberpsychology, Behavior, and Social Networking* 2016;**19:**134-40.

Martin PD, Dutton GR, Rhode PC, Horswell RL, Ryan DH, Brantley PJ. Weight loss maintenance following a primary care intervention for low-income minority women. *Obesity* 2008;**16:**2462-7.

Mayer-Davis EJ, D'Antonio AM, Smith SM, Kirkner G, Martin SL, Parra-Medina D, et al. Pounds off with empowerment (POWER): A clinical trial of weight management strategies for black and white adults with diabetes who live in medically underserved rural communities. *American Journal of Public Health* 2004;**94:**1736-42.

McRobbie H, Hajek P, Peerbux S, Kahan BC, Eldridge S, Trepel D, et al. Tackling obesity in areas of high social deprivation: clinical effectiveness and cost-effectiveness of a task-based weight management group programme - a randomised controlled trial and economic evaluation. *Health Technology Assessment* 2016;**20(79).**.

Melin I, Karlstrom B, Lappalainen R, Berglund L, Mohsen R, Vessby B. A programme of behaviour modification and nutrition counselling in the treatment of obesity: A randomised 2-y clinical trial. *International Journal of Obesity* 2003;**27:**1127-35.

Mensinger JL, Calogero RM, Stranges S, Tylka TL. A weight-neutral versus weight-loss approach for health promotion in women with high BMI: A randomized-controlled trial. *Appetite* 2016;**105:**364-74.

Miles JM, Leiter L, Hollander P, Wadden T, Anderson JW, Doyle M, et al. Effect of orlistat in overweight and obese patients with type 2 diabetes treated with metformin. *Diabetes Care* 2002;**25:**1123-8.

Mingrone G, Greco AV, Giancaterini A, Scarfone A, Castagneto M, Pugeat M. Sex hormone-binding globulin levels and cardiovascular risk factors in morbidly obese subjects before and after weight reduction induced by diet or malabsorptive surgery. *Atherosclerosis* 2002;**161:**455-62.

Mingrone G, Panunzi S, De Gaetano A, Guidone C, Iaconelli A, Leccesi L, et al. Bariatric surgery versus conventional medical therapy for type 2 diabetes. *New England Journal of Medicine* 2012;**366:**1577-85.

Moreno B, Bellido D, Sajoux I, Goday A, Saavedra D, Crujeiras AB, et al. Comparison of a very low-calorie-ketogenic diet with a standard low-calorie diet in the treatment of obesity. *Endocrine* 2014;**47:**793-805.

Nackers LM, Middleton KR, Dubyak PJ, Daniels MJ, Anton SD, Perri MG. Effects of prescribing 1,000 versus 1,500 kilocalories per day in the behavioral treatment of obesity: A randomized trial. *Obesity* 2013;**21:**2481-7.

Nilsen V, Bakke PS, Gallefoss F. Effects of lifestyle intervention in persons at risk for type 2 diabetes mellitus - results from a randomised, controlled trial. *BMC public health* 2011;**11:**893.

O'Neil PM, Miller-Kovach K, Tuerk PW, Becker LE, Wadden TA, Fujioka K, et al. Randomized Controlled Trial of a Nationally Available Weight Control Program Tailored for Adults with Type 2 Diabetes. *Obesity* 2016;**24:**2269-77.

Ostbye T, Stroo M, Brouwer RJ, Peterson BL, Eisenstein EL, Fuemmeler BF, et al. Steps to Health employee weight management randomized Control trial: Short-term follow-up results. *Journal of Occupational and Environmental Medicine* 2015;**57:**188-95.

Pascale RW, Wing RR, Butler BA, Mullen M, Bononi P. Effects of a behavioral weight loss program stressing calorie restriction versus calorie plus fat restriction in obese individuals with NIDDM or a family history of diabetes. *Diabetes Care* 1995;**18:**1241-8.

Pedersen E, Jesudason DR, Clifton PM. High protein weight loss diets in obese subjects with type 2 diabetes mellitus. *Nutrition, Metabolism and Cardiovascular Diseases* 2014;**24:**554-62.

Pekkarinen T, Kaukua J, Mustajoki P. Long-term weight maintenance after a 17-week weight loss intervention with or without a one-year maintenance program: A randomized controlled trial. *Journal of Obesity* 2015;Article 651460

Perri MG, Limacher MC, Durning PE, Janicke DM, Lutes LD, Bobroff LB, et al. Extended-care programs for weight management in rural communities: The Treatment of Obesity in Underserved Rural Settings (TOURS) randomized trial. *Archives of Internal Medicine* 2008;**168:**2347-54.

Perri MG, Limacher MC, Von Castel-Roberts K, Daniels MJ, Durning PE, Janicke DM, et al. Comparative effectiveness of three doses of weight-loss counseling: Two-year findings from the Rural LITE Trial. *Obesity* 2014;**22:**2293-300.

Perri MG, Nezu AM, McKelvey WF, Shermer RL, Renjilian DA, Viegener BJ. Relapse prevention training and problem-solving therapy in the long-term management of obesity. *Journal of Consulting and Clinical Psychology* 2001;**69:**722-6.

Poston WS, Reeves RS, Haddock CK, Stormer S, Balasubramanyam A, Satterwhite O, et al. Weight loss in obese Mexican Americans treated for 1-year with orlistat and lifestyle modification. *International Journal of Obesity & Related Metabolic Disorders* 2003;**27:**1486-93.

Purcell K, Sumithran P, Prendergast LA, Bouniu CJ, Delbridge E, Proietto J. The effect of rate of weight loss on long-term weight management: A randomised controlled trial. *The Lancet Diabetes and Endocrinology* 2014;**2:**954-62.

Rapoport L, Clark M, Wardle J. Evaluation of a modified cognitive-behavioural programme for weight management. *International Journal of Obesity* 2000;**24:**1726-37.

Reichard A, Saunders MD, Saunders RR, Donnelly JE, Lauer E, Sullivan DK, et al. A comparison of two weight management programs for adults with mobility impairments. *Disability and Health Journal* 2015;**8:**61-9.

Reis LO, Favaro WJ, Barreiro GC, Oliveira LC, Chaim EA, Fregonesi A, et al. Erectile dysfunction and hormonal imbalance in morbidly obese male is reversed after gastric bypass surgery: a prospective randomized controlled trial. *International journal of andrology* 2010;**33:**736-44.

Richelsen B, Tonstad S, Rossner S, Toubro S, Niskanen L, Madsbad S, et al. Effect of orlistat on weight regain and cardiovascular risk factors following a very-low-energy diet in abdominally obese patients: A 3-year randomized, placebo-controlled study. *Diabetes Care* 2007;**30:**27-32.

Rock CL, Flatt SW, Pakiz B, Taylor KS, Leone AF, Brelje K, et al. Weight loss, glycemic control, and cardiovascular disease risk factors in response to differential diet composition in a weight loss program in type 2 diabetes: A randomized controlled trial. *Diabetes Care* 2014;**37:**1573-80.

Rossner S, Flaten H. VLCD versus LCD in long-term treatment of obesity. *International Journal of Obesity* 1997;**21:**22-6.

Ryttig KR, Flaten H, Rossner S. Long-term effects of a very low calorie diet (Nutrilett) in obesity treatment. A prospective, randomized, comparison between VLCD and a hypocaloric diet + behavior modification and their combination. *International Journal of Obesity* 1997;**21:**574-9.

Ryttig KR, Rossner S. Weight maintenance after a very low calorie diet (VLCD) weight reduction period and the effects of VLCD supplementation. A prospective, randomized, comparative, controlled long-term trial. *Journal of Internal Medicine* 1995;**238:**299-306.

Schauer PR, Kashyap SR, Wolski K, Brethauer SA, Kirwan JP, Pothier CE, et al. Bariatric surgery versus intensive medical therapy in obese patients with diabetes. *New England Journal of Medicine* 2012;**366:**1567-76.

Shikany JM, Thomas AS, Beasley TM, Lewis CE, Allison DB. Randomized controlled trial of the Medifast 5 & 1 plan for weight loss. *International Journal of Obesity* 2013;**37:**1571-8.

Sjostrom L, Rissanen A, Andersen T, Boldrin M, Golay A, Koppeschaar HPF, et al.

Randomised placebo-controlled trial of orlistat for weight loss and prevention of weight regain in obese patients. *Lancet* 1998;**352:**167-72.

Soenen S, Bonomi AG, Lemmens SGT, Scholte J, Thijssen MAMA, Van Berkum F, et al. Relatively high-protein or 'low-carb' energy-restricted diets for body weight loss and body weight maintenance? *Physiology and Behavior* 2012;**107:**374-80.

Spring B, Duncan JM, Janke EA, Kozak AT, McFadden HG, Demott A, et al. Integrating technology into standard weight loss treatment a randomized controlled trial. *JAMA Internal Medicine* 2013;**173:**105-11.

Stahre L, Hallstrom T. A short-term cognitive group treatment program gives substantial weight reduction up to 18 months from the end of treatment. A randomized controlled trial. *Eating and Weight Disorders* 2005;**10:**51-8.

Stenius-Aarniala B, Poussa T, Kvarnstrom J, Gronlund EL, Ylikahri M, Mustajoki P. Immediate and long term effects of weight reduction in obese people with asthma: Randomised controlled study. *British Medical Journal* 2000;**320:**827-32.

Stern L, Iqbal N, Seshadri P, Chicano KL, Daily DA, McGrory J, et al. The Effects of Low-Carbohydrate versus Conventional Weight Loss Diets in Severely Obese Adults: One-Year Follow-up of a Randomized Trial. *Annals of Internal Medicine* 2004;**140:**778-85+I-27.

Strobl V, Knisel W, Landgraf U, Faller H. A combined planning and telephone aftercare intervention for obese patients: effects on physical activity and body weight after one year. *Journal of rehabilitation medicine* 2013;**45:**198-205.

Swinburn BA, Carey D, Hills AP, Hooper M, Marks S, Proietto J, et al. Effect of orlistat on cardiovascular disease risk in obese adults. *Diabetes Obesity & Metabolism* 2005;**7:**254-62.

Torgerson J, Agren L, Sjostrom L. Effects on body weight of strict or liberal adherence to an initial period of VLCD treatment. A randomised, one-year clinical trial of obese subjects. *International Journal of Obesity* 1999;**23:**190-7.

Torgerson JS, Hauptman J, Boldrin MN, Sjostrom L. XENical in the Prevention of Diabetes in Obese Subjects (XENDOS) Study: A randomized study of orlistat as an adjunct to lifestyle changes for the prevention of type 2 diabetes in obese patients. *Diabetes Care* 2004;**27:**155-61.

Torgerson JS, Lissner L, Lindroos AK, Kruijer H, Sjostrom L. VLCD plus dietary and behavioural support versus support alone in the treatment of severe obesity. A randomised two-year clinical trial. *International Journal of Obesity* 1997;**21:**987-94.

Tsai AG, Wadden TA, Rogers MA, Day SC, Moore RH, Islam BJ. A Primary Care Intervention for Weight Loss: Results of a Randomized Controlled Pilot Study. *Obesity* 2010;**18:**1614-8.

Van Name MA, Camp AW, Magenheimer EA, Li F, Dziura JD, Montosa A, et al. Effective translation of an intensive lifestyle intervention for hispanic women with prediabetes in a community health center setting. *Diabetes Care* 2016;**39:**525-31.

Villareal DT, Chode S, Parimi N, Sinacore DR, Hilton T, Armamento-Villareal R, et al. Weight loss, exercise, or both and physical function in obese older adults. *New England Journal of Medicine* 2011;**364:**1218-29.

Villareal DT, Shah K, Banks MR, Sinacore DR, Klein S. Effect of weight loss and exercise therapy on bone metabolism and mass in obese older adults: A one-year randomized controlled trial. *Journal of Clinical Endocrinology and Metabolism* 2008;**93:**2181-7.

von Gruenigen VE, Courneya KS, Gibbons HE, Kavanagh MB, Waggoner SE, Lerner E. Feasibility and effectiveness of a lifestyle intervention program in obese endometrial cancer patients: A randomized trial. *Gynecologic Oncology* 2008;**109:**19-26.

Wadden TA, Foster GD, Letizia KA. One-year behavioral treatment of obesity: Comparison of moderate and severe caloric restriction and the effects of weight maintenance therapy. *Journal of Consulting and Clinical Psychology* 1994;**62:**165-71.

Wadden TA, Berkowitz RI, Vogt RA, Steen SN, Stunkard AJ, Foster GD. Lifestyle modification in the pharmacologic treatment of obesity: a pilot investigation of a potential primary care approach. *Obesity research* 1997;**5:**218-26.

Wadden TA, Vogt RA, Foster GD, Anderson DA. Exercise and the maintenance of weight loss: 1-year follow-up of a controlled clinical trial. *Journal of Consulting and Clinical Psychology* 1998;**66:**429-33.

Wadden TA, Volger S, Sarwer DB, Vetter ML, Tsai AG, Berkowitz RI, et al. A two-year randomized trial of obesity treatment in primary care practice. *New England Journal of Medicine* 2011;**365:**1969-79.

Weinstock RS, Trief PM, Cibula D, Morin PC, Delahanty LM. Weight Loss Success in Metabolic Syndrome by Telephone Interventions: Results from the SHINE Study. *Journal of General Internal Medicine* 2013;**28:**1620-8.

West DS, DiLillo V, Bursac Z, Gore SA, Greene PG. Motivational interviewing improves weight loss in women with type 2 diabetes. *Diabetes Care* 2007;**30:**1081-7.

West DS, Gorin AA, Subak LL, Foster G, Bragg C, Hecht J, et al. A motivation-focused weight loss maintenance program is an effective alternative to a skill-based approach. *International Journal of Obesity* 2011;**35:**259-69.

Wing RR, Blair E, Marcus M, Epstein LH, Harvey J. Year-long weight loss treatment for obese patients with type II diabetes: Does including an intermittent very-low-calorie diet improve outcome? *American Journal of Medicine* 1994;**97:**354-62.

Wing RR, Marcus MD, Epstein LH, Jawad A. A "family-based" approach to the treatment of obese type II diabetic patients. *Journal of Consulting and Clinical Psychology* 1991;**59:**156-62.

Wing RR, Marcus MD, Salata R, Epstein LH, Miaskiewicz S, Blair EH. Effects of a very-low-calorie diet on long-term glycemic control in obese type 2 diabetic subjects. *Archives of Internal Medicine* 1991;**151:**1334-40.

Wing RR, Venditti E, Jakicic JM, Polley BA, Lang W. Lifestyle intervention in overweight individuals with a family history of diabetes. *Diabetes Care* 1998;**21:**350-9.

Wolf AM, Conaway MR, Crowther JQ, Hazen KY, Nadler JL, Oneida B, et al. Translating lifestyle intervention to practice in obese patients with type 2 diabetes: Improving Control with Activity and Nutrition (ICAN) study. *Diabetes Care* 2004;**27:**1570-6.

Wylie-Rosett J, Swencionis C, Ginsberg M, Cimino C, Wassertheil-Smoller S, Caban A, et al. Computerized weight loss intervention optimizes staff time: the clinical and cost results of a controlled clinical trial conducted in a managed care setting. *Journal of the American Dietetic Association* 2001;**101:**1155-62; quiz 63-64.

Yeh MC, Rodriguez E, Nawaz H, Gonzalez M, Nakamoto D, Katz DL. Technical skills for weight loss: 2-y follow-up results of a randomized trial. *International Journal of Obesity* 2003;**27:**1500-6.
