# Supplementary material for: Does weight management research for adults with severe obesity represent them? Analysis of systematic review data
Source: BMJ Open. 2022 May 30;12(5):e054459. doi: 10.1136/bmjopen-2021-054459 (PMC9157335; doi:10.1136/bmjopen-2021-054459)
Supplement: Author's manuscript [file bmjopen-2021-054459.draft_revisions.pdf]

BMJ Open is committed to open peer review. As part of this commitment we make the peer review history of every article we publish publicly available.

When an article is published we post the peer reviewers' comments and the authors' responses online. We also post the versions of the paper that were used during peer review. These are the versions that the peer review comments apply to.

The versions of the paper that follow are the versions that were submitted during the peer review process. They are not the versions of record or the final published versions. They should not be cited or distributed as the published version of this manuscript.

BMJ Open is an open access journal and the full, final, typeset and author-corrected version of record of the manuscript is available on our site with no access controls, subscription charges or pay-per-view fees (http://bmjopen.bmj.com).

If you have any questions on BMJ Open's open peer review process please email info.bmjopen@bmj.com

BMJ Open

Does weight management research for adults with severe obesity represent them? Analysis of systematic review data.

Journal:	*BMJ Open*
Manuscript ID	bmjopen-2021-054459
Article Type:	Original research
Date Submitted by the Author:	12-Jun-2021
Complete List of Authors:	Robertson, Clare; University of Aberdeen, Health Services Research Unit<br>Aceves-Martins, Magaly; University of Aberdeen, Health Services Research Unit<br>Cruickshank, Moira; University of Aberdeen, Health Services Research Unit<br>Imamura, Mari; University of Aberdeen, Health Services Research Unit<br>Avenell, Alison; University of Aberdeen, Health Services Research Unit
Keywords:	PUBLIC HEALTH, Clinical trials < THERAPEUTICS, Health policy < HEALTH SERVICES ADMINISTRATION & MANAGEMENT

SCHOLARONE™
Manuscripts

I, the Submitting Author has the right to grant and does grant on behalf of all authors of the Work (as defined in the below author licence), an exclusive licence and/or a non-exclusive licence for contributions from authors who are: i) UK Crown employees; ii) where BMJ has agreed a CC-BY licence shall apply, and/or iii) in accordance with the terms applicable for US Federal Government officers or employees acting as part of their official duties; on a worldwide, perpetual, irrevocable, royalty-free basis to BMJ Publishing Group Ltd ("BMJ") its licensees and where the relevant Journal is co-owned by BMJ to the co-owners of the Journal, to publish the Work in this journal and any other BMJ products and to exploit all rights, as set out in our licence.

The Submitting Author accepts and understands that any supply made under these terms is made by BMJ to the Submitting Author unless you are acting as an employee on behalf of your employer or a postgraduate student of an affiliated institution which is paying any applicable article publishing charge ("APC") for Open Access articles. Where the Submitting Author wishes to make the Work available on an Open Access basis (and intends to pay the relevant APC), the terms of reuse of such Open Access shall be governed by a Creative Commons licence – details of these licences and which Creative Commons licence will apply to this Work are set out in our licence referred to above.

Other than as permitted in any relevant BMJ Author's Self Archiving Policies, I confirm this Work has not been accepted for publication elsewhere, is not being considered for publication elsewhere and does not duplicate material already published. I confirm all authors consent to publication of this Work and authorise the granting of this licence.

For peer review only

Does weight management research for adults with severe obesity represent them? Analysis of systematic review data.

Clare Robertson (ORCiD ID 0000-0001-6019-6795), Magaly Aceves-Martins (ORCiD ID 0000-0002-9441-142X), Moira Cruickshank (ORCiD ID 0000-0002-5182-884X), Mari Imamura (ORCiD ID 0000-0003-4871-0354), Alison Avenell (ORCiD ID 0000-0003-4813-5628)

Clare Robertson, Research Fellow, Health Services Research Unit, University of Aberdeen, Aberdeen, UK

Magaly Aceves-Martins, Research Fellow, Health Services Research Unit, University of Aberdeen, Aberdeen, UK, The Rowett Institute, University of Aberdeen, Aberdeen, UK

Moira Cruickshank, Research Fellow, Health Services Research Unit, University of Aberdeen, Aberdeen, UK

Mari Imamura, Research Fellow, Health Services Research Unit, University of Aberdeen, Aberdeen, UK

Alison Avenell, Professor, Health Services Research Unit, University of Aberdeen, Aberdeen, UK

Corresponding author: Clare Robertson, Health Services Research Unit, University of Aberdeen, Health Sciences Building, Foresterhill, Aberdeen, UK, Telephone: +44 (0) 1224 438086, Email: c.robertson@abdn.ac.uk

Manuscript word count: 3150

For peer review only - http://bmjopen.bmj.com/site/about/guidelines.xhtml

ABSTRACT

Objective

To determine the extent to which current evidence from long-term randomised controlled trials (RCTs) of weight management, which are most likely to inform guidelines and practice, are generalisable and applicable to those most at risk, particularly under-served adult groups with severe obesity (BMI $\geq$35kg/m^2).

Design

Descriptive analysis of 131 RCTs included in a comprehensive systematic review of long-term weight management interventions, including diet, lifestyle, bariatric surgery and orlistat, for adults with BMI$\geq$35kg/m^2, as part of the REBALANCE project (NIHR HTA15/09/04). Reporting of trial inclusion and exclusion criteria, trial recruitment strategies, baseline characteristics, and outcomes were analysed using a pre-defined list of characteristics informed by the PROGRESS-Plus framework and the UK Equality Act 2010.

Setting

RCTs delivered in any setting in any country.

Study selection

RCTs published after 1990 with $\geq$1 year of follow-up. Eligible interventions were focused on lifestyle change, bariatric surgery, or orlistat.

Results

Few (6.1%) trials reported adapting recruitment to appeal to under-served groups. Only 10.0% reported culturally adapting their trial materials. Only 6.1% of trials gave any

justification for their exclusion criteria, yet over half excluded participation for age or mental health reasons. Just over half of the trials reported participants' race or ethnicity, and only a fifth reported socioeconomic status. Where outcomes were reported for under-served groups, the most common analysis was by sex (47.3%) followed by race or ethnicity (16.8%). Only 3.1% of trials reported outcomes according to socioeconomic status.

Conclusions and relevance

Trialists should justify inclusion and exclusion criteria and report sufficient data to allow comparisons between their populations and those for whom the interventions most apply. Guidance for considering under-served groups may improve the appropriateness of research and inform greater engagement of under-served communities with health and social care services.

Systematic review registration

PROSPERO CRD42016040190

For peer review only

For peer review only - http://bmjopen.bmj.com/site/about/guidelines.xhtml

ARTICLE SUMMARY

Strengths and limitations of this study

- A unique analysis exploring whether randomised trials evaluating interventions for weight management for people with severe obesity consider the needs of under-served groups
- Data set includes up-to-date, best available randomised controlled trial evidence (considered to be the highest level of evidence to inform guidelines and clinical practice) identified using robust and exhaustive literature search strategies.
- Data set includes wide-ranging weight management interventions delivered in different settings, from an international perspective
- Our analysis was limited by poor reporting of whether subgroup reporting by under-served groups was planned and whether trials were adequately powered to detect subgroup differences
- Non-randomised study designs, interventions with shorter follow-up, and unpublished studies may have been more inclusive in their designs and reporting.

For peer review only - http://bmjopen.bmj.com/site/about/guidelines.xhtml

Background

In high-income countries, and increasingly in low and middle-income countries, lower incomes, less education, lower socioeconomic status, and disability are associated with greater risks of obesity for adults.[1-5] Most of these risk factors are stronger for women than men, [1-3,6] although men may be less likely to undertake weight management programmes.[7] Being an adult with obesity is associated with a lower health-related quality of life than a healthy weight adult of the same socioeconomic status.[8] More severe obesity, such as BMI 35kg/m^2 or more, with its associated greater risks for comorbidities, reduced quality of life and premature mortality [9] is particularly related to lower socioeconomic status, [1, 10] and intellectual and physical disabilities.[4] Poorer outcomes from COVID-19 are also strongly related to obesity, particularly severe obesity.[5] For countries such as the US and UK, some racial or ethnic groups may also be at much greater risk of obesity, especially severe obesity. [10, 11]

Preventing obesity and providing effective interventions, particularly for people with more severe obesity, is, therefore, one of the major public health challenges and vital in terms of addressing health inequalities. While organisations such as the U.S. Food and Drug Administration (FDA),[12-14] the National Institutes of Health (NIH)[15] and the National Institutes of Health Research (NIHR)[16] have produced guidelines on the inclusion of individuals of all ages, sexes/genders, races and ethnicities, and other physical, sensory/perceptual, cognitive and emotional characteristics, there is a lack of accessible policy-ready evidence on what works in terms of interventions to reduce inequalities in obesity. It is also recognised that some groups, (for example, socially disadvantaged, less educated, and minority race or ethnic groups) may be less likely to be recruited into randomised controlled trials (RCTs) for lifestyle interventions. It is, therefore, important to

For peer review only - http://bmjopen.bmj.com/site/about/guidelines.xhtml

understand the extent to which the current evidence base is applicable to those who are most at risk of experiencing poorer obesity-related health outcomes and have more severe obesity.

This study aimed to determine the extent to which the findings from intervention studies of weight management, as exemplified by long-term randomised controlled trials (RCTs), are generalisable and applicable to those most at risk, particularly under-served groups with severe obesity. To examine these questions, we set out:

1. to describe inclusion and exclusion criteria for RCTs of adult weight management interventions, and in those trials:
2. to describe efforts to tailor recruitment strategies to improve recruitment of people from under-served groups,
3. to describe efforts to culturally adapt interventions for these groups,
4. to describe reported baseline characteristics and outcomes for these groups.

Methods

Our dataset comprised 131 RCTs included in a systematic review of weight management interventions for adults with severe obesity (BMI>35kg/m^2), as part of the REBALANCE project (NIHR HTA15/09/04).[9] Eligible interventions included diet, lifestyle, bariatric surgery, or orlistat. RCTs were restricted to publications after 1990 up to May 2017 to reflect more recent practice. Trials had to report long-term data on weight change (≥ 1 year of follow-up). The decision to focus on long-term RCTs for this study was informed by the preference for high-quality, long-term evidence of lasting effectiveness in guideline documents [17, 18] and are, therefore, most likely to influence treatment policy decisions. We attempted to contact the first, second and last authors of the main publications to identify all additional materials (i.e. protocols, trial materials and diet books) to inform our data

For peer review only - http://bmjopen.bmj.com/site/about/guidelines.xhtml

extraction. Full details of the REBALANCE project, including the search strategy, have been published.[9] The PRISMA (Preferred Reporting Items for Systematic Reviews and Meta-Analyses) flow chart is presented in Appendix 1. Ethical approval was not required.

In the absence of definitions of under-served groups, we identified under-served groups by using protected characteristics informed by the PROGRESS-Plus framework.[19] and the UK Equality Act 2010.[20] Four reviewers (MA-M, MC, MI and CR) conducted double data coding of each RCT for their reporting of trial inclusion and exclusion criteria, trial recruitment strategies, baseline characteristics, and outcome reporting for the following characteristic groups, with disagreements resolved by consensus:

- Older age
- Physical health
- Mental health (including, but not limited to, depression, psychosis, schizophrenia, substance abuse and eating disorders)
- Comorbidities (e.g. Types 1 and 2 diabetes mellitus)
- Gender/sex (including RCTs recruiting only men or women)
- Sexual orientation
- Gender reassignment
- Marriage or civil partnership status
- Pregnancy
- Religion or belief
- Place of residence/housing (including residents of supported accommodation and homeowner status)
- Race or ethnicity
- Language

For peer review only - http://bmjopen.bmj.com/site/about/guidelines.xhtml

- Occupational status
- Education/literacy
- Socioeconomic status (SES), including individual SES and participants recruited from rural or disadvantaged geographical locations
- Social capital (including social support networks and/or social isolation)
- PROGRESS Plus (personal characteristics associated with discrimination (e.g. age, disability), features of relationships (e.g. smoking parents, excluded from school), time-dependent relationships (e.g. leaving the hospital, respite care, other instances where a person may be temporarily at a disadvantage)

For a trial to be coded as having adapted their recruitment strategy for an under-served group, additional efforts to employ strategies that would appeal to that particular group (e.g. held recruitment days in particular settings or developed recruitment materials in multiple languages) had to be demonstrated. Trials that solely recruited from one characteristic group using conventional recruitment methods (e.g. newspaper or radio advertisements) were not coded as having an adapted recruitment strategy. Similarly, trials had to demonstrate that their interventions were designed with an under-served group in mind to be coded as having delivered a culturally adapted intervention. The focus of this study was to provide a description of trial methods and trial reporting to answer each of our research questions in relation to under-served groups; therefore, no formal statistical analysis was conducted.

Patient and public involvement

Although we did not consult patient representatives for this particular analysis, three patient representatives were members of the REBALANCE project Advisory Group, who

For peer review only - http://bmjopen.bmj.com/site/about/guidelines.xhtml

contributed to developing the research questions, data interpretation and reporting of the research findings.

Results

From the 131 included trials, 41 (31.3%) provided us with additional materials for their publications. The majority (81/131, 61.8%) of included studies were set in North America (80 in the USA and 1 in the USA and Canada), 41 out of 131 (31.3%) were in European countries (including 8 in the UK), 8 (6.1%) were in the Southern Hemisphere (six in Australia, one in New Zealand and one in Australia and New Zealand) and one in Brazil. None of the trials were set in low-income countries. Just under half (62/131, 47.3%) of the studies were published between 2011 and 2017. Only five (3.8%) trials were linked to publications reporting qualitative data.[21-25] Few trials had follow-up durations longer than 12 months, with the exceptions of the US Look AHEAD trial [26] (median duration of 9.6 years), and four trials with follow-up times of five years. [27-30] The Look AHEAD trial was the largest trial, including over 5000 participants. Interventions were wide ranging, including very low calorie and calorie reducing diets, orlistat, bariatric surgery and lifestyle weight management programmes. Details of the characteristics of the included studies can be found in the supplementary files of the REBALANCE report.[9]

Trial recruitment

More than half of the trials (71/131, 54%) recruited participants either solely or partially through a health service provider. Recruitment methods were unclear or not reported in 18 trials (13%). Recruitment methods for the other trials were mainly advertisements in local newspapers or other media. Based on their reporting, only three (2.3%) trials were judged to have adapted their recruitment strategies to appeal to under-served groups. These strategies

For peer review only - http://bmjopen.bmj.com/site/about/guidelines.xhtml

included holding pre-recruitment presentations in US schools,[31] recruitment events at football stadia of Scottish Premier League football clubs [23] and having bilingual staff take informed consent and provide written consent forms in both English and Spanish languages.[32] It was unclear in a further five (3.8%) trials whether recruitment strategies had been adapted beyond conventional methods.[33-37]

Seven trials (5.3%, six from the US and one from New Zealand) recruited participants from diverse racial or ethnic groups and reported cultural adaptations to their interventions. Five (3.8%) of these trials [37-41] included advice on regional or culturally adapted recipes and foods for specific ethnic groups. Two trials (1.5%) [32, 42] had interventions that were delivered by bilingual staff. One trial [42] also reported that the intervention was designed for delivery in populations with limited literacy and numeracy and impaired access to health-promoting resources.

A further five trials (all from the US) [43-47] sought recruitment from specific racial or ethnic groups and reported intervention adaptations to increase cultural salience. Four of these trials [43-46] included culturally specific dietary advice and recipes. One trial described including bilingual interventions [43] and three trials described including interventionists from specific racial or ethnic groups.[47,45,46] Two trials [45,46] described using logos and programme identification "for African-Americans", with one of these trials [45] including a video greeting from an African-American principal investigator.

The number of trials reporting inclusion and exclusion criteria by protected characteristic groups is presented in Table 1. Four older trials (3.1%) [48-51] did not report any inclusion criteria, and inclusion criteria was unclear in one further study.[52] Seven (5.3%) [49, 53-58] trials

For peer review only

did not report any explicit exclusion criteria and did not report that they had no exclusion criteria. Eight (6.1%) trials reported either full [23, 25, 44, 59-61] or partial [31, 62] justification for their exclusion criteria. Over half (58.0%) of the trials reported excluding people with mental health conditions and 44.2% excluded people with substance abuse or addiction issues. The majority of trials also excluded adults from older age groups and based on current or planned pregnancy.

Twenty-one (16.0%) trials were judged to have inclusion criteria that might have implicitly excluded certain disadvantaged groups, such as people who do not have healthcare insurance,[63,64] people who do not belong to a particular religious community group,[65] people without regular internet [22] or telephone [66] access, and English language comprehension.[31,42,59,67-77] In a further 40 (30.5%) trials, it was unclear if trial recruitment could have implicitly excluded disadvantaged groups. Few trials reported their inclusion criteria so as to include particular under-served groups or were judged to have made efforts to maximise trial recruitment from these groups. When trial recruitment was targeted, this was usually to recruit women only (19 trials) or people with Type 2 diabetes (28 trials).

For peer review only

Table 1 The number (and percent) of trials (n=131) reporting inclusion and exclusion criteria by protected characteristics included in the REBALANCE systematic review of RCTs

	**Inclusion**			**Exclusion**
	Protected characteristic is reported in the inclusion criteria	Protected characteristic is not reported in inclusion criteria, but an effort was made to recruit from the protected characteristic group	Unclear if the protected characteristic was targeted for inclusion or if the trial unintentionally recruited solely/mainly from the protected characteristic group	Protected characteristic is reported in the exclusion criteria, or the reported inclusion criteria clearly excluded the protected characteristic
Physical health	10 (7.6%)	0	0	51 (38.9%)
Diabetes Type 1	0	0	0	15 (11.5%)
Diabetes Type 2	28 (21.4%)	0	0	29 (22.1%)
Diabetes (Type 1 and 2 or Type not reported)	0	0	0	3 (2.3%)
Mental health	6 (4.6%)	0	0	76 (58.0%)
Substance abuse	0	0	0	58 (44.2%)
Eating disorder	0	0	0	35 (26.7%)
Race/ethnicity	7 (5.3%)	5 (3.8%)	2 (1.5%)	1 (0.8%)
Lower income/sociodemographic group/residents of rural area	5 (3.8%)	1 (0.8%)	0	0
Women only	19 (14.5%)	0	4 (3.1%)	0
Pregnancy	0	0	0	72 (54.9%)
Men only	4 (3.0%)	0	1 (0.76%)	5 (3.8%)
Older age	2 (1.5%)	1 (0.8%)	0	82 (62.6%)[a]
Language	0	0	0	16 (12.2%)
Marital status	1 (0.76%)	0	0	1 (0.76%)

a. includes 8 RCTs recruiting participants up to 75 years, 1 RCT recruited participants up to 76 years and 3 RCTs recruited participants aged up to 80 years

For peer review only

Reporting of baseline characteristics

Details of the number of trials reporting baseline characteristics of their participants by each of the protected characteristic groups are presented in Table 2. The majority of trials reported age (99.2%) and sex (97.7%) in their description of baseline participant characteristics. Just over half of the trials (58.0%) reported race or ethnicity. Education history was less well-reported (40.5%). Socioeconomic status was reported by 22.9%, and occupation status by 21.4%. Of the trials that were not specifically for people with diabetes, six (4.6%) included diabetes in their reporting of baseline characteristics. Few trials reported details of the other protected characteristics, and none reported details of gender reassignment, sexual orientation or pregnancy.

For peer review only

Table 2 The number (and percent) of trials (n=131) reporting protected characteristics at baseline in the REBALANCE systematic review of RCTs

	Protected characteristic is reported at baseline for each intervention arm	Protected characteristic is reported at baseline for the whole trial	Total
Age	126 (96.2%)	4 (3.0%)	130 (99.2%)
Physical health	10 (7.6%)	-	10 (7.6%)
Mental health	10 (7.6%)	-	10 (7.6%)
Diabetes	6 (4.6%)	-	6 (4.6%)
Sex	126 (96.2%)	2 (1.5%)	128 (97.7%)
Gender reassignment	0	0	0
Sexual orientation	0	0	0
Marriage/civil partnership status	38 (29.0%)	-	38 (29.0%)
Pregnancy	0	0	0
Place of residence/housing	6 (4.6%)	-	6 (4.6%)
Occupation status	27 (20.6%)	1 (0.8%)	28 (21.4%)
Education/literacy	51 (38.9%)	2 (1.5%)	53 (40.5%)
Socioeconomic status	29 (22.1%)	1 (0.8%)	30 (22.9%)
Social capital	2 (1.5%)	-	2 (1.5%)
Religion/belief	1 (0.8%)	-	1 (0.8%)
Race/ethnicity	74 (56.5%)	2 (1.5%)	76 (58.0%)
PROGRESS Plus	2 (1.5%)	-	2 (1.5%)

For peer review only

Outcome reporting

Details of the number of trials reporting outcomes by each of the protected characteristics are shown in Table 3. Very few trials reported outcomes by protected characteristic groups. Where outcomes were reported by protected characteristics, the most common group was sex (47.3%) followed by race or ethnicity (16.8%).

For peer review only

Table 3 The number (and percent) of trials (n=131) reporting outcome data for protected characteristics in the REBALANCE systematic review of RCTs

	Trial recruitment was targeted at people from the protected characteristic group	One or more outcome(s) reported for the protected characteristic in planned subgroup analysis	One or more outcome(s) reported for the protected characteristic – unclear if subgroup analysis was pre-planned	Total
Older Age	2 (1.5%)[a]	2 (1.5%)	3 (2.3%)	7 (5.3%)
Physical health	10 (7.6%)	0	0	10 (7.6%)
Mental health	6 (4.6%)	0	2 (1.5%)	8 (6.1%)
Diabetes	28 (21.3%)	0	1 (0.8%)	29 (22.1%)
Sex	23 (17.5%) [b]	17 (13.0%)	22 (16.8%)	62 (47.3%)
Gender reassignment	0	0	0	0
Sexual orientation	0	0	0	0
Marriage/civil partnership status	1 (0.76%)	1 (0.8%)	3 (2.3%)	5 (3.8%)
Pregnancy	0	0	0	0
Place of residence/housing	0	2 (1.5%)	0	2 (1.5%)
Occupation status	2 (1.5%)	2 (1.5%)	4 (3.1%)	8 (6.1%)
Education/literacy	0	1 (0.8%)	6 (4.6%)	7 (5.3%)
Socioeconomic status	5 (3.8%)	4 (3.1%)	3 (2.3%)	12 (9.2%)
Social capital	1 (0.8%)	3 (2.3%)	2 (1.5%)	6 (4.6%)
Religion/belief	1 (0.8%)	0	0	1 (0.8%)
Race/ethnicity	8 (6.1%)	5 (3.8%)	9 (6.9%)	22 (16.8%)
PROGRESS Plus	0	0	0	0

a. Both trials recruited participants aged ≥65 years
b. 19 women only trials, 4 men only trials

For peer review only

Discussion

Main findings

Our findings demonstrate that most trialists testing weight management strategies to help adults with severe obesity fail to consider populations who are most at risk of poorer health outcomes. Almost all trials were from high-income countries, where lower socioeconomic status and income are associated with a greater prevalence of obesity, particularly severe obesity.[1-5] At most, 6.1% of trials reported adapting recruitment to appeal to under-served groups. 10.0% reported culturally adapting their trial materials for ethnic groups or people with limited English language literacy or numeracy. Only 6.1% of trials gave any justification for their exclusion criteria, yet more than half excluded participation for age or mental health reasons. Just over half of the trials reported participants' race or ethnicity, and only around a fifth reported socioeconomic status. Where outcomes were reported for under-served groups, the most common analysis was by sex (47.3%) followed by race or ethnicity (16.8%); however, where analyses were presented as subgroups, it was often unclear whether these analyses were planned or unplanned. This is a finding that was also demonstrated by Liu and colleagues (2020),[78] who highlighted a lack of transparent reporting of intentions to analyse race and ethnicity subgroups in Cochrane intervention reviews. Only 3.1% of the trials we reviewed reported outcomes according to socioeconomic status.

Although we were limited by trial reporting and the information contained in additional materials obtained from around a third of trials, our findings are concerning. In almost all trials, it would be impossible to assess the generalisability of findings to the wider population of adults with severe obesity. There is clear evidence [1-5,10-11] that under-served groups with lower incomes, less education, lower socioeconomic status, intellectual and physical disabilities, or poorer mental health are more at risk of obesity, particularly severe obesity, in

For peer review only - http://bmjopen.bmj.com/site/about/guidelines.xhtml

high-income countries, especially the US which provided the majority of trials examined. Nevertheless, the lack of reporting for characteristics reflecting under-served groups suggests that trial investigators did not consider including them in the design, recruitment, analysis or reporting of their interventions.

Very few trials appeared to have engaged people with obesity in the design of services, which is important, given that a systematic review of qualitative research by Sutcliffe and colleagues [79] showed how service users have perspectives that should inform weight management services to improve their reach. From systematic reviews, researchers have clearly demonstrated the need to involve communities in all stages of research in order to enhance the engagement and generalisability of that research, acknowledging that this requires extended timeframes and greater costs.[80,81] For example, Ni She and colleagues (2018) [82] undertook a rapid realist review of the mechanisms and resources needed to engage under-served, seldom-heard groups in health and social care research, with items grouped by an expert panel under the headings of environmental and social planning, service provision, guidelines, fiscal measures, communication and marketing, and regulation and legislation. In the US, Arnegard and colleagues (2020)[83] have also called on the NIH's stakeholder groups to redouble their efforts to encourage sex- and gender-aware reporting of biomedical investigations. This is a call that we endorse following the findings from our previous systematic review of weight management interventions for men with obesity.[7] Our review highlighted the paucity of evidence for men, who are less likely to take part in weight management interventions and the lack of engagement of men in all aspects of intervention design, and optimal trial recruitment processes of weight management. [7]

For peer review only - http://bmjopen.bmj.com/site/about/guidelines.xhtml

While the reasons for the under-representation of under-served groups in RCTs are likely to be complex and multifaceted, with many known and unknown barriers to participation, there is evidence that, for some groups, willingness to participate is not a predominant factor.[84] Mindful of the need to improve the engagement of under-served groups in research in the UK, the National Institute for Health Research set up the INCLUDE project, [16] which has led to the INCLUDE ethnicity framework; [85] providing four key questions on who should be involved in research, and how to facilitate involvement. Others have investigated and found a lack of external validity in trials for people with asthma,[86] type 2 diabetes,[87, 88] and neurological disease,[89] or a failure to justify exclusion criteria in trials of cardiovascular disease prevention [90] other than for safety reasons. These publications did not consider the socioeconomic status or educational attainment of trial participants. In a systematic review of 305 trials of clinical conditions, He and colleagues [91] found high exclusion rates in trials for people with hypertension (83.0%), lipid-lowering drugs in primary prevention (85.9%), type 2 diabetes (81.7%), COPD (84.3%) and asthma (96.0%), with no strong evidence that exclusion rates had changed with time.

More recently, others have also highlighted the small number of intervention studies testing out weight management for under-served adults,[92] or policies to assist socioeconomically disadvantaged groups. [93] A 2015 systematic review of interventions aimed at reducing socioeconomic inequalities for adults with obesity [94] found that primary care-delivered tailored weight loss programmes and group weight loss interventions had the most evidence of potential effectiveness in reducing obesity, at least in the short term, among low-income women, but there were few individual-level intervention studies and a lack of long term evidence of effectiveness.

For peer review only - http://bmjopen.bmj.com/site/about/guidelines.xhtml

Our literature search attempted to identify all long-term randomised trials published since 1990 for adults with BMI ≥35kg/m^2 irrespective of the type of lifestyle intervention, including comparisons with orlistat and bariatric surgery. Although we included publications in any language from any country, we cannot exclude the possibility that we failed to find some trials, particularly those from low income countries, which might not be listed in the databases we searched.

We did not contact authors for the additional information extracted for this analysis and were limited by poor reporting by trial authors. Similarly, some smaller trials might have been underpowered to detect differences in treatment effects between subgroups. Nevertheless, we consider these characteristics to be pivotal to interpreting these trials into real-world guidance and services, so would expect their presentation in trial publications, especially at baseline. We assessed long-term RCT evidence because it is most likely to inform guidance on weight management. Other study designs, interventions with shorter follow-up, and unpublished studies may have been more inclusive in their designs and reporting.

We used categories informed by the PROGRESS-PLUS framework [19] and the UK Equality Act 2010 [20] as the key characteristics for identifying those under-served participants that should be considered for study design, public patient involvement, recruitment, analysis and reporting, not just for trials of weight management, but trials generally. O'Neill (2014)[19] has shown how the prior PROGRESS framework can be used as an equity lens for systematic reviews and methodological studies; however, NIHR's INCLUDE project has recently published a more extensive list of categories of under-served groups to consider with regard to representation in trials.[95]

For peer review only - http://bmjopen.bmj.com/site/about/guidelines.xhtml

Recommendations for research

Trialists should justify inclusion and exclusion criteria, and report sufficient data to allow comparisons between their populations and the populations for whom the interventions apply. NIHR's INCLUDE project's ethnicity framework provides important factors to consider with regard to ethnic groups (https://www.trialforge.org/trial-forge-centre/include/), which can help provide transferable considerations for other under-served groups. However, a wider equity lens may be needed in the face of groups with multiple disadvantages. Although guidance for research will aid considerations of equity, we do not yet have ways of assessing when proportional representation in larger trials and sub-group reporting for under-served groups is insufficient. This research should be explicitly conducted with and for these under-served groups.

Conclusions and Recommendations for practice

Although we have focussed on research, similar issues of poor reporting, lack of generalisability, and less engagement with under-served groups are even more relevant for health and social care services. Thus, guidance for research may improve the appropriateness of that research and help inform greater engagement of under-served communities with health and social care services.

For peer review only - http://bmjopen.bmj.com/site/about/guidelines.xhtml

REFERENCES

1. Booth HP, Charlton J, Gulliford MC. Socioeconomic inequality in morbid obesity with body mass index more than 40 kg/m^2 in the United States and England. *SSM Popul Health* 2017;3:172–178
2. Devaux M, Sassi F. Social inequalities in obesity and overweight in 11 OECD countries. *Eur J Public Health* 2013; 23(3):464–469
3. El-Sayed AM, Scarborough P, Galea S. Unevenly distributed: a systematic review of the health literature about socioeconomic inequalities in adult obesity in the United Kingdom. *BMC Public Health* 2012, 12:18
4. Hsieh K, Rimmer JH, Heller T. Obesity and associated factors in adults with intellectual disability. *J Intellect Disabil Res* 2014;58(9):851-863
5. Public Health England. Excess Weight and COVID-19 Insights from new evidence [Internet]. London: PHE publications; 2021 [cited February 2021]. Available from: https://assets.publishing.service.gov.uk/government/uploads/system/uploads/attachment_data/file/907966/PHE_insight_Excess_weight_and_COVID-19__FINAL.pdf
6. Grech A, Allman-Farinelli M. Prevalence and period trends of overweight and obesity in Australian young adults. *Eur J Clin Nutr* 2016; 70: 1083–1085
7. Robertson C, Archibald D, Avenell A, et al. Systematic reviews of and integrated report on the quantitative, qualitative and economic evidence base for the management of obesity in men. *Health Technol Assess* 2014;18(35).
8. Minet Kinge J, Morris S. Socioeconomic variation in the impact of obesity on health-related quality of life. *Soc Sci Med* 2010;71:1864e1871
9. Avenell A, Robertson C, Skea Z, et al. Bariatric surgery, lifestyle interventions and orlistat for severe obesity: the REBALANCE mixed-methods systematic review and economic evaluation. *Health Technol Assess* 2018;22(68).

For peer review only - http://bmjopen.bmj.com/site/about/guidelines.xhtml

10. Green MA, Rowe F. Explaining the widening distribution of Body Mass Index: A decomposition analysis of trends for England, 2002–2004 and 2012–2014. *Area* 2020;1–11.

11. Ogden CL, Fryar CD, Martin CB, et al. Trends in Obesity Prevalence by Race and Hispanic Origin—1999-2000 to 2017-2018. *JAMA* 2020;324(12):1208–1210

12. U.S.Food and Drug Administration (FDA). Guidance for industry: collection of race and ethnicity data in clinical trials. https://www.fda.gov/media/75453/download Accessed March 2021

13. U.S.Food and Drug Administration (FDA). Evaluation and reporting of age-, race-, and ethnicity-specific data in medical device clinical studies. https://www.fda.gov/media/98686/download Accessed March 2021

14. U.S.Food and Drug Administration (FDA). Design considerations for devices intended for home use. https://www.fda.gov/media/84830/download Accessed March 2021

15. National Institutes of Health [internet]. U.S. 2019. Inclusion policies for research involving human subjects. https://grants.nih.gov/policy/inclusion.htm (accessed March 2021)

16. National Institute for Health Research [Internet]. London: The Institute; 2020 [cited February 2021]. Improving inclusion of under-served groups in clinical research: Guidance from the NIHR INCLUDE project. Available from: www.nihr.ac.uk/documents/improving-inclusion-of-under-served-groups-in-clinical-research-guidance-from-include-project/25435

17. National Institute for Health and Care Excellence. Obesity: identification assessment and management. London; NICE 2015. URL https://www.nice.org.uk/guidance/cg189 (accessed May 2021)

For peer review only - http://bmjopen.bmj.com/site/about/guidelines.xhtml

18. National Institute for Health and Care Excellence. Weight management: lifestyle services for overweight or obese adults. London; NICE 2014. URL https://www.nice.org.uk/guidance/ph53 (accessed May 2021)

19. O'Neill J, Tabish H, Welch V, et al. Applying an equity lens to interventions: using PROGRESS ensures consideration of socially stratifying factors to illuminate inequities in health. *J Clin Epidemiol* 2014, 67(1):56-64

20. Government Equalities Office, Equality and Human Rights Commission [Internet]. London: Government Digital Service; 2013 [cited February 2021]. Equality Act 2010: guidance. Available from: https://www.gov.uk/guidance/equality-act-2010-guidance

21. Yeh MC, Rodriguez E, Nawaz H, et al. Technical skills for weight loss: 2-y follow-up results of a randomized trial. *Int J Obes Relat Metab Disord* 2003;27:1500–6

22. Little P, Stuart B, Hobbs FR, et al. Randomised controlled trial and economic analysis of an internet-based weight management programme: POWeR+ (Positive Online Weight Reduction). *Health Technol Assess* 2017;21(4).

23. Hunt K, Gray CM, Maclean A, Smillie S, Bunn C, Wyke S. Do weight management programmes delivered at professional football clubs attract and engage high risk men? A mixed-methods study. *BMC Public Health* 2014;14:50

24. McRobbie H, Hajek P, Peerbux S, et al. Tackling obesity in areas of high social deprivation: clinical effectiveness and cost-effectiveness of a task-based weight management group programme – a randomised controlled trial and economic evaluation. *Health Technol Assess* 2016;20(79).

25. Green CA, Yarborough BJ, Leo MC, et al. Weight maintenance following the STRIDE lifestyle intervention for individuals taking antipsychotic medications. *Obesity* 2015;23:1995–2001.

For peer review only - http://bmjopen.bmj.com/site/about/guidelines.xhtml

26. The Look AHEAD Research Group. Cardiovascular effects of intensive lifestyle intervention in type 2 diabetes. N Engl J Med 2013;369:145–54.

27. Hakala P, Karvetti RL, Rönnemaa T. Group vs. individual weight reduction programmes in the treatment of severe obesity – a five year follow-up study. Int J Obes Relat Metab Disord 1993;17:97–102.

28. Hakala P. Weight reduction programmes at a rehabilitation centre and a health centre based on group counselling and individual support: short- and long-term follow-up study. Int J Obes Relat Metab Disord 1994;18:483–9.

29. Mingrone G, Greco AV, Giancaterini A, Scarfone A, Castagneto M, Pugeat M. Sex hormone binding globulin levels and cardiovascular risk factors in morbidly obese subjects before and after weight reduction induced by diet or malabsorptive surgery. Atherosclerosis 2002;161:455–62.

30. Schauer PR, Kashyap SR, Wolski K, Brethauer SA, Kirwan JP, Pothier CE, et al. Bariatric surgery versus intensive medical therapy in obese patients with diabetes. N Engl J Med 2012;366:1567–76.

31. Berry DC, Schwartz TA, McMurray RG, et al. The family partners for health study: a cluster randomized controlled trial for child and parent weight management. *Nutr Diabetes* 2014;4:e101

32. Van Name MA, Camp AW, Magenheimer EA, et al. Effective translation of an intensive lifestyle intervention for Hispanic women with prediabetes in a community health center setting. *Diabetes Care* 2016;39:525–31

33. Hauptman J, Lucas C, Boldrin MN, Collins H, Segal KR. Orlistat in the long-term treatment of obesity in primary care settings. *Arch Fam Med* 2000;9:160–7

34. Kahleova H, Hill M, Pelikanova T. Vegetarian vs. conventional diabetic diet – a 1-year follow-up. *Cor Vasa* 2014;56:e140–4

For peer review only

35. Kelley DE, Bray GA, Pi-Sunyer FX, et al. Clinical efficacy of orlistat therapy in overweight and obese patients with insulin-treated type 2 diabetes: a 1-year randomized controlled trial. *Diabetes Care* 2002;25:1033–41

36. Khoo J, Piantadosi C, Duncan R, et al. Comparing effects of a low-energy diet and a high-protein low-fat diet on sexual and endothelial function, urinary tract symptoms, and inflammation in obese diabetic men. *J Sex Med* 2011;8:2868–75

37. Kumanyika SK, Fassbender JE, Sarwer DB, et al. One-year results of the Think Health! study of weight management in primary care practices. *Obesity* 2012;20:1249–57

38. Krebs JD, Elley CR, Parry-Strong A, et al. The Diabetes Excess Weight Loss (DEWL) Trial: a randomised controlled trial of high-protein versus high-carbohydrate diets over 2 years in type 2 diabetes. *Diabetologia* 2012;55:905–14

39. Mayer-Davis EJ, D'Antonio AM, Smith SM, et al. Pounds off with empowerment (POWER): a clinical trial of weight management strategies for black and white adults with diabetes who live in medically underserved rural communities. *Am J Public Health* 2004;94:1736–42

40. Perri MG, Limacher MC, Durning PE, et al. Extended-care programs for weight management in rural communities: the Treatment of Obesity in Underserved Rural Settings (TOURS) randomized trial. *Arch Intern Med* 2008;168:2347–54

41. Perri MG, Limacher MC, von Castel-Roberts K, et al. Comparative effectiveness of three doses of weight-loss counseling: two-year findings from the rural LITE trial. *Obesity* 2014;22:2293–300

42. Bennett GG, Warner ET, Glasgow RE, et al. Obesity treatment for socioeconomically disadvantaged patients in primary care practice. *Arch Intern Med* 2012;172:565–74

For peer review only - http://bmjopen.bmj.com/site/about/guidelines.xhtml

43. Poston WS, Reeves RS, Haddock CK, et al. Weight loss in obese Mexican Americans treated for 1-year with orlistat and lifestyle modification. *Int J Obes Relat Metab Disord* 2003;27:1486–93

44. Martin PD, Dutton GR, Rhode PC, Horswell RL, Ryan DH, Brantley PJ. Weight loss maintenance following a primary care intervention for low-income minority women. *Obesity* 2008;16:2462–7.

45. Kumanyika SK, Shults J, Fassbender J, et al. Outpatient weight management in African-Americans: the Healthy Eating and Lifestyle Program (HELP) study. *Prev Med* 2005;41:488–502

46. Kumanyika SK, Wadden TA, Shults J, et al. Trial of family and friend support for weight loss in African American adults. *Arch Intern Med* 2009;169:1795–804

47. Fitzgibbon ML, Stolley MR, Schiffer L, Sharp LK, Singh V, Dyer A. Obesity Reduction Black Intervention Trial (ORBIT): 18-month results. Obesity 2010;18:2317–25

48. Agras WS, Berkowitz RI, Arnow BA, et al. Maintenance following a very-low-calorie diet. *J Consult Clin Psychol* 1996;64:610–13

49. Dennison KF, Galante D, Dennison D, Golaszewski T. A one year post-program assessment of a computer-assisted instruction weight management program for industrial employees: lessons learned. *J Health Educ* 1996;27:38–43

50. Wadden TA, Berkowitz RI, Vogt RA, Steen SN, Stunkard AJ, Foster GD. Lifestyle modification in the pharmacologic treatment of obesity: a pilot investigation of a potential primary care approach. *Obes Res* 1997;5:218–26

51. Wadden TA, Vogt RA, Foster GD, Anderson DA. Exercise and the maintenance of weight loss: 1-year follow-up of a controlled clinical trial. *J Consult Clin Psychol* 1998;66:429–33

For peer review only

52. Mingrone G, Greco AV, Giancaterini A, Scarfone A, Castagneto M, Pugeat M. Sex hormone-binding globulin levels and cardiovascular risk factors in morbidly obese subjects before and after weight reduction induced by diet or malabsorptive surgery. Atherosclerosis 2002;161:455–62

53. Torgerson JS, Lissner L, Lindroos AK, Kruijer H, Sjöström L. VLCD plus dietary and behavioural support versus support alone in the treatment of severe obesity. A randomised two-year clinical trial. *Int J Obes Relat Metab Disord* 1997;21:987–94

54. Wing RR, Marcus MD, Epstein LH, Jawad A. A 'family-based' approach to the treatment of obese type II diabetic patients. *J Consult Clin Psychol* 1991;59:156–62

55. Melin I, Karlström B, Lappalainen R, Berglund L, Mohsen R, Vessby B. A programme of behaviour modification and nutrition counselling in the treatment of obesity: a randomised 2-y clinical trial. *Int J Obes Relat Metab Disord* 2003;27:1127–35

56. Pascale RW, Wing RR, Butler BA, Mullen M, Bononi P. Effects of a behavioral weight loss program stressing calorie restriction versus calorie plus fat restriction in obese individuals with NIDDM or a family history of diabetes. *Diabetes Care* 1995;18:1241–8.

57. Perri MG, Nezu AM, McKelvey WF, Shermer RL, Renjilian DA, Viegener BJ. Relapse prevention training and problem-solving therapy in the long-term management of obesity. *J Consult Clin Psychol* 2001;69:722–6

58. Richelsen B, Tonstad S, Rössner S, et al. Effect of orlistat on weight regain and cardiovascular risk factors following a very-low-energy diet in abdominally obese patients: a 3-year randomized, placebo-controlled study. *Diabetes Care* 2007;30:27–32

For peer review only - http://bmjopen.bmj.com/site/about/guidelines.xhtml

59. Azar KM, Xiao L, Ma J. Baseline obesity status modifies effectiveness of adapted diabetes prevention program lifestyle interventions for weight management in primary care. *Biomed Res Int* 2013;2013:191209

60. Cummings DE, Arterburn DE, Westbrook EO, et al. Gastric bypass surgery vs intensive lifestyle and medical intervention for type 2 diabetes: the CROSSROADS randomised controlled trial. *Diabetologia* 2016;59:945–53

61. Daubenmier J, Moran PJ, Kristeller J, et al. Effects of a mindfulness-based weight loss intervention in adults with obesity: a randomized clinical trial. *Obesity* 2016;24:794–804

62. Dixon JB, Schachter LM, O'Brien PE, et al. Surgical vs. conventional therapy for weight loss treatment of obstructive sleep apnea: a randomized controlled trial. *JAMA* 2012;308:1142–9.

63. Dutton GR, Nackers LM, Dubyak PJ, et al. A randomized trial comparing weight loss treatment delivered in large versus small groups. *Int J Behav Nutr Phys Act* 2014;11:123

64. Ostbye T, Stroo M, Brouwer RJ, et al. Steps to Health employee weight management randomized control trial: Short-term follow-up results. J Occup Environ Med 2015;57:188-95

65. Latner JD, Ciao AC, Wendicke AU, Murakami JM, Durso LE. Community-based behavioural weight-loss treatment: long-term maintenance of weight loss, physiological, and psychological outcomes. *Behav Res Ther* 2013;51:451–9

66. Lowe MR, Butryn ML, Thomas JG, Coletta M. Meal replacements, reduced energy density eating, and weight loss maintenance in primary care patients: a randomized controlled trial. *Obesity* 2014;22:94–100

For peer review only

67. Bartels SJ, Pratt SI, Aschbrenner KA, et al. Clinically significant improved fitness and weight loss among overweight persons with serious mental illness. *Psychiatr Serv* 2013;64:729–36

68. Bliddal H, Leeds AR, Stigsgaard L, Astrup A, Christensen R. Weight loss as treatment for knee osteoarthritis symptoms in obese patients: 1-year results from a randomised controlled trial. *Ann Rheum Dis* 2011;70:1798–803

69. Christensen P, Frederiksen R, Bliddal H, et al. Comparison of three weight maintenance programs on cardiovascular risk, bone and vitamins in sedentary older adults. *Obesity* 2013;21:1982–90.

70. Damschroder LJ, Lutes LD, Kirsh S, et al. Small-changes obesity treatment among veterans: 12-month outcomes. *Am J Prev Med* 2014;47:541–53.

71. Eaton CB, Hartman SJ, Perzanowski E, et al. A randomized clinical trial of a tailored lifestyle intervention for obese, sedentary, primary care patients. *Ann Fam Med* 2016;14:311–19

72. Ströbl V, Knisel W, Landgraf U, Faller H. A combined planning and telephone aftercare intervention for obese patients: effects on physical activity and body weight after one year. *J Rehabil Med* 2013;45:198–205

73. Wolf AM, Conaway MR, Crowther JQ, et al. Improving Control with Activity and Nutrition (ICAN) Study. Translating lifestyle intervention to practice in obese patients with type 2 diabetes: Improving Control with Activity and Nutrition (ICAN) study. *Diabetes Care* 2004;27:1570–6

74. Mensinger JL, Calogero RM, Stranges S, Tylka TL. A weight-neutral versus weight-loss approach for health promotion in women with high BMI: a randomized-controlled trial. *Appetite* 2016;105:364–74

For peer review only - http://bmjopen.bmj.com/site/about/guidelines.xhtml

75. Nackers LM, Middleton KR, Dubyak PJ, Daniels MJ, Anton SD, Perri MG. Effects of prescribing 1,000 versus 1,500 kilocalories per day in the behavioral treatment of obesity: a randomized trial. *Obesity* 2013;21:2481–7.
76. Nilsen V, Bakke PS, Gallefoss F. Effects of lifestyle intervention in persons at risk for type 2 diabetes mellitus – results from a randomised, controlled trial. *BMC Public Health* 2011;11:893
77. O'Neil PM, Miller-Kovach K, Tuerk PW, et al. Randomized controlled trial of a nationally available weight control program tailored for adults with type 2 diabetes. *Obesity* 2016;24:2269–77
78. Liu P, Ross JS, Ioannidis JP, Dhruva SS, Vasiliou V, Wallach JD. Prevalence and significance of race and ethnicity subgroup analyses in Cochrane intervention reviews. Clinical Trials. 2020;17(2):231-234
79. Sutcliffe K, Melendez-Torres GJ, Burchet, HED, Richardso, M, Rees R, Thomas J. The importance of service-users' perspectives: A systematic review of qualitative evidence reveals overlooked critical features of weight management programmes. *Health Expect* 2018;21: 563– 573
80. De Las Nueces, D, Hacker K, DiGirolamo, A, Hicks LS. A Systematic Review of Community-Based Participatory Research to Enhance Clinical Trials in Racial and Ethnic Minority Groups. *Health Serv Res* 2012;47:1363-1386
81. Bonevski, B., Randell, M., Paul, C. et al. Reaching the hard-to-reach: a systematic review of strategies for improving health and medical research with socially disadvantaged groups. *BMC Med Res Methodol* 2014;14:42
82. Ní Shé, É, Morton, S, Lambert, V, et al. Clarifying the mechanisms and resources that enable the reciprocal involvement of seldom heard groups in health and social care

For peer review only - http://bmjopen.bmj.com/site/about/guidelines.xhtml

research: A collaborative rapid realist review process. *Health Expect* 2019; 22: 298–306

83. Arnegard ME, Whitten LA, Hunter C, and Clayton JA. Sex as a Biological Variable: A 5-Year Progress Report and Call to Action. *J Women's Health*. 2020.858-864.
84. Wendler D, Kington R, Madans J, Van Wye G, Christ-Schmidt H, et al. Are racial and ethnic minorities less willing to participate in health research? PLoS Med 2006; 3(2): e19.
85. Trial Forge [Internet]. Aberdeen, Scotland: Trial Forge; 2020 [cited February 2021]. The INCLUDE ethnicity framework. Available from: https://www.trialforge.org/trial-forge-centre/include/
86. Travers J, Marsh S, Williams M, et al. External validity of randomised controlled trials in asthma: to whom do the results of the trials apply? *Thorax* 2007;62:219-223
87. Saunders CD, Byrne B, Guthrie RS, et al. External validity of randomized controlled trials of glycaemic control and vascular disease: how representative are participants? *Diabet Med* 2013;30:300– 308
88. Hoppe C, Kerr D. Minority underrepresentation in cardiovascular outcome trials for type 2 diabetes. *Lancet Diabetes Endocrinol* 2017;5(1):13
89. Trivedi RB, Humphreys K. Participant exclusion criteria in treatment research on neurological disorders: Are unrepresentative study samples problematic? *Contemp Clin Trials* 2015;44:20–25
90. Schmid AF, Groenwold RHH, van Deldena JJM, et al. Justification of exclusion criteria was underreported in a review of cardiovascular trials. *J Clin Epidemiol* 2014;67:635-644
91. He J, Morales DR, Guthrie B. Exclusion rates in randomized controlled trials of treatments for physical conditions: a systematic review. *Trials* 2020;21:228

For peer review only - http://bmjopen.bmj.com/site/about/guidelines.xhtml

92. Hayba, N, Partridge SR, Nour MM, Grech A, Allman Farinelli M. Effectiveness of lifestyle interventions for preventing harmful weight gain among young adults from lower socioeconomic status and ethnically diverse backgrounds: a systematic review. Obes Rev 2018;19:333– 346
93. Olstad DL, Ancilotto R, Teychenne M, et al. Can targeted policies reduce obesity and improve obesity-related behaviours in socioeconomically disadvantaged populations? A systematic review. Obes Rev 2017;18:791– 807
94. Bambra CL, Hillier FC, Cairns J-M, Kasim A, Moore HJ, Summerbell CD. How effective are interventions at reducing socioeconomic inequalities in obesity among children and adults? Two systematic reviews. *Public Health Res* 2015;3(1).
95. Witham MD, Anderson E, Carroll C, et al. Developing a roadmap to improve trial delivery for under-served groups: results from a UK multi-stakeholder process. *Trials* 2020;21:694

Acknowledgements

We thank the members of the REBALANCE Project and Advisory Groups for their contributions to the REBALANCE project. We thank Shaun Treweek and Heidi Gardner, Health Services Research Unit, University of Aberdeen, for helpful discussions on trial generalisability and inclusion of under-served groups.

Competing interests

None.

Author Contributorship

For peer review only - http://bmjopen.bmj.com/site/about/guidelines.xhtml

CR, MA-M, MC, MI, AA contributed to conception and design, acquisition of data, or analysis and interpretation of data, and revised data critically for important intellectual content of this manuscript. All authors critically reviewed the manuscript and approved the final version submitted for publication. CR takes responsibility for the integrity of the data and the accuracy of the data analysis.

Funding

The REBALANCE study was funded by the National Institute for Health Research (NIHR) Health Technology Assessment Programme (Project number: 15/09/04). The views expressed are those of the author(s) and not necessarily those of the NHS, the NIHR or the Department of Health. The Health Services Research Unit is funded by the Chief Scientist Office of the Scottish Government Health and Social Care Directorates.

Transparency statement

CR affirms that the manuscript is an honest, accurate, and transparent account of the study being reported; that no important aspects of the study have been omitted; and that any discrepancies from the study as planned have been explained

Data sharing

Study data are all available from the NIHR journals library ((REBALANCE) REview of Behaviour And Lifestyle interventions for severe obesity: AN evidenCE synthesis (nihr.ac.uk) or can be obtained from the lead author.

For peer review only - http://bmjopen.bmj.com/site/about/guidelines.xhtml

Appendix 1 PRISMA flow diagram

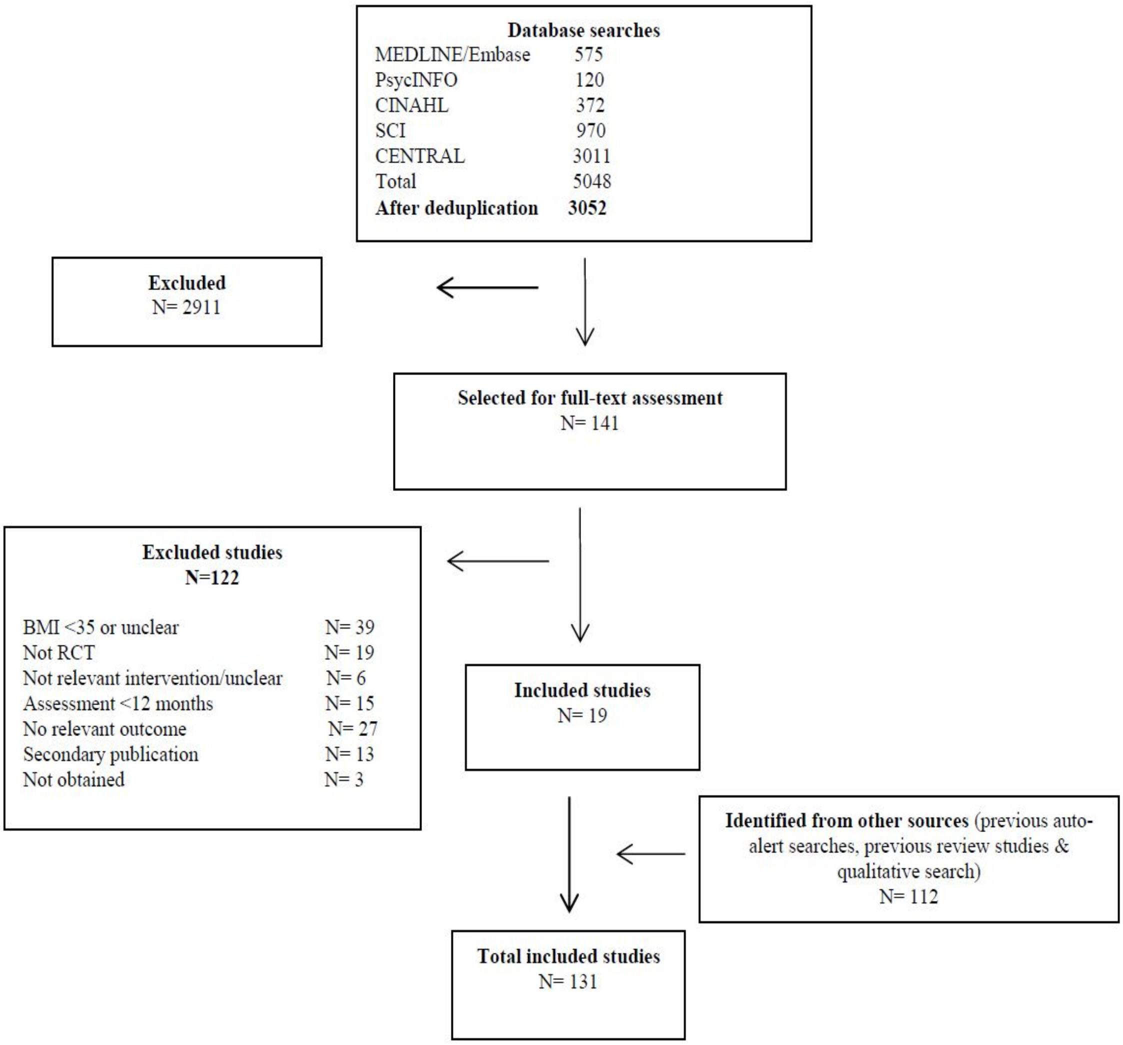

PRISMA 2009 Checklist

Section/topic	#	Checklist item	Reported on page #
**TITLE**			
Title	1	Identify the report as a systematic review, meta-analysis, or both.	The report is not a meta-analysis. It is an analysis of data included in a systematic review. This is identified by the manuscript title.
**ABSTRACT**			
Structured summary	2	Provide a structured summary including, as applicable: background; objectives; data sources; study eligibility criteria, participants, and interventions; study appraisal and synthesis methods; results; limitations; conclusions and implications of key findings; systematic review registration number.	2-3
**INTRODUCTION**			
Rationale	3	Describe the rationale for the review in the context of what is already known.	5-6
Objectives	4	Provide an explicit statement of questions being addressed with reference to participants, interventions, comparisons, outcomes, and study design (PICOS).	6
**METHODS**			
Protocol and registration	5	Indicate if a review protocol exists, if and where it can be accessed (e.g., Web address), and, if available, provide registration information including registration number.	3, 6
Eligibility criteria	6	Specify study characteristics (e.g., PICOS, length of follow-up) and report characteristics (e.g., years considered, language, publication status) used as criteria for eligibility, giving rationale.	6 (and available from the REBALANCE main report)
Information sources	7	Describe all information sources (e.g., databases with dates of coverage, contact with study authors to identify additional studies) in the search and date last searched.	6

For peer review only

PRISMA 2009 Checklist

Section/topic	#	Checklist item	Reported on page #
Search	8	Present full electronic search strategy for at least one database, including any limits used, such that it could be repeated.	6 (available from the REBALANCE main report)
Study selection	9	State the process for selecting studies (i.e., screening, eligibility, included in systematic review, and, if applicable, included in the meta-analysis).	6
Data collection process	10	Describe method of data extraction from reports (e.g., piloted forms, independently, in duplicate) and any processes for obtaining and confirming data from investigators.	6-8
Data items	11	List and define all variables for which data were sought (e.g., PICOS, funding sources) and any assumptions and simplifications made.	6-8
Risk of bias in individual studies	12	Describe methods used for assessing risk of bias of individual studies (including specification of whether this was done at the study or outcome level), and how this information is to be used in any data synthesis.	6 (available from the REBALANCE main report)
Summary measures	13	State the principal summary measures (e.g., risk ratio, difference in means).	6-8
Synthesis of results	14	Describe the methods of handling data and combining results of studies, if done, including measures of consistency (e.g., I^2) for each meta-analysis.	6-8

Section/topic	#	Checklist item	Reported on page #
Risk of bias across studies	15	Specify any assessment of risk of bias that may affect the cumulative evidence (e.g., publication bias, selective reporting within studies).	6 (risk of bias assessment is available from the REBALANCE main report)
Additional analyses	16	Describe methods of additional analyses (e.g., sensitivity or subgroup analyses, meta-regression), if done, indicating which were pre-specified.	NA
**RESULTS**			
Study selection	17	Give numbers of studies screened, assessed for eligibility, and included in the review, with reasons for exclusions at each stage, ideally with a flow diagram.	Appendix 1
Study characteristics	18	For each study, present characteristics for which data were extracted (e.g., study size, PICOS, follow-up period) and provide the citations.	6 (available from the

For peer review only - http://bmjopen.bmj.com/site/about/guidelines.xhtml

PRISMA 2009 Checklist

			Rebalance main report)
Risk of bias within studies	19	Present data on risk of bias of each study and, if available, any outcome level assessment (see item 12).	6 (available from the Rebalance main report)
Results of individual studies	20	For all outcomes considered (benefits or harms), present, for each study: (a) simple summary data for each intervention group (b) effect estimates and confidence intervals, ideally with a forest plot.	8-16
Synthesis of results	21	Present results of each meta-analysis done, including confidence intervals and measures of consistency.	12, 14, 16
Risk of bias across studies	22	Present results of any assessment of risk of bias across studies (see Item 15).	6 (available from the REBALANCE main report)
Additional analysis	23	Give results of additional analyses, if done (e.g., sensitivity or subgroup analyses, meta-regression [see Item 16]).	NA
**DISCUSSION**			
Summary of evidence	24	Summarize the main findings including the strength of evidence for each main outcome; consider their relevance to key groups (e.g., healthcare providers, users, and policy makers).	17-19
Limitations	25	Discuss limitations at study and outcome level (e.g., risk of bias), and at review-level (e.g., incomplete retrieval of identified research, reporting bias).	20
Conclusions	26	Provide a general interpretation of the results in the context of other evidence, and implications for future research.	21
**FUNDING**			
Funding	27	Describe sources of funding for the systematic review and other support (e.g., supply of data); role of funders for the systematic review.	34

From: Moher D, Liberati A, Tetzlaff J, Altman DG, The PRISMA Group (2009). Preferred Reporting Items for Systematic Reviews and Meta-Analyses: The PRISMA Statement. PLoS Med 6(7): e1000097. doi:10.1371/journal.pmed1000097

For more information, visit: **www.prisma-statement.org**.

For peer review only - http://bmjopen.bmj.com/site/about/guidelines.xhtml

BMJ Open

Does weight management research for adults with severe obesity represent them? Analysis of systematic review data.

Journal:	*BMJ Open*
Manuscript ID	bmjopen-2021-054459.R1
Article Type:	Original research
Date Submitted by the Author:	10-Nov-2021

Complete List of Authors:	Robertson, Clare; University of Aberdeen, Health Services Research Unit<br>Aceves-Martins, Magaly; University of Aberdeen, Health Services Research Unit<br>Cruickshank, Moira; University of Aberdeen, Health Services Research Unit<br>Imamura, Mari; University of Aberdeen, Health Services Research Unit<br>Avenell, Alison; University of Aberdeen, Health Services Research Unit

**Primary Subject Heading**:	Health services research
Secondary Subject Heading:	Health policy, Public health
Keywords:	PUBLIC HEALTH, Clinical trials < THERAPEUTICS, Health policy < HEALTH SERVICES ADMINISTRATION & MANAGEMENT

SCHOLARONE™
Manuscripts

I, the Submitting Author has the right to grant and does grant on behalf of all authors of the Work (as defined in the below author licence), an exclusive licence and/or a non-exclusive licence for contributions from authors who are: i) UK Crown employees; ii) where BMJ has agreed a CC-BY licence shall apply, and/or iii) in accordance with the terms applicable for US Federal Government officers or employees acting as part of their official duties; on a worldwide, perpetual, irrevocable, royalty-free basis to BMJ Publishing Group Ltd ("BMJ") its licensees and where the relevant Journal is co-owned by BMJ to the co-owners of the Journal, to publish the Work in this journal and any other BMJ products and to exploit all rights, as set out in our licence.

The Submitting Author accepts and understands that any supply made under these terms is made by BMJ to the Submitting Author unless you are acting as an employee on behalf of your employer or a postgraduate student of an affiliated institution which is paying any applicable article publishing charge ("APC") for Open Access articles. Where the Submitting Author wishes to make the Work available on an Open Access basis (and intends to pay the relevant APC), the terms of reuse of such Open Access shall be governed by a Creative Commons licence – details of these licences and which Creative Commons licence will apply to this Work are set out in our licence referred to above.

Other than as permitted in any relevant BMJ Author's Self Archiving Policies, I confirm this Work has not been accepted for publication elsewhere, is not being considered for publication elsewhere and does not duplicate material already published. I confirm all authors consent to publication of this Work and authorise the granting of this licence.

For peer review only

Does weight management research for adults with severe obesity represent them? Analysis of systematic review data.

Clare Robertson (ORCiD ID 0000-0001-6019-6795), Magaly Aceves-Martins (ORCiD ID 0000-0002-9441-142X), Moira Cruickshank (ORCiD ID 0000-0002-5182-884X), Mari Imamura (ORCiD ID 0000-0003-4871-0354), Alison Avenell (ORCiD ID 0000-0003-4813-5628)

Clare Robertson, Research Fellow, Health Services Research Unit, University of Aberdeen, Aberdeen, UK

Magaly Aceves-Martins, Research Fellow, Health Services Research Unit, University of Aberdeen, Aberdeen, UK, The Rowett Institute, University of Aberdeen, Aberdeen, UK

Moira Cruickshank, Research Fellow, Health Services Research Unit, University of Aberdeen, Aberdeen, UK

Mari Imamura, Research Fellow, Health Services Research Unit, University of Aberdeen, Aberdeen, UK

Alison Avenell, Professor, Health Services Research Unit, University of Aberdeen, Aberdeen, UK

Corresponding author: Clare Robertson, Health Services Research Unit, University of Aberdeen, Health Sciences Building, Foresterhill, Aberdeen, UK, Telephone: +44 (0) 1224 438086, Email: c.robertson@abdn.ac.uk

Manuscript word count: 3976

For peer review only - http://bmjopen.bmj.com/site/about/guidelines.xhtml

ABSTRACT

Objective

Our objective was to determine the extent to which current evidence from long-term randomised controlled trials (RCTs) of weight management are generalisable and applicable to under-served adult groups with obesity (body mass index [BMI] $\geq$35kg/m^2).

Methods

Descriptive analysis of 131 RCTs, published after 1990 to May 2017 with ≥1 year of follow-up, included in a systematic review of long-term weight management interventions, for adults with BMI ≥35kg/m^2(the REBALANCE project). Studies were identified from MEDLINE, EMBASE, PsychINFO, SCI, CENTRAL, and from hand-searching. . Reporting of trial inclusion and exclusion criteria, trial recruitment strategies, baseline characteristics, and outcomes were analysed using a pre-defined list of characteristics informed by the PROGRESS (Place of residence, Race/ethnicity/culture/language, Occupation, Gender/sex, Religion, Education, Socioeconomic status, Social capital) -Plus framework and the UK Equality Act 2010.

Results

Few (6.1%) trials reported adapting recruitment to appeal to under-served groups. 10.0% reported culturally adapting their trial materials. Only 6.1% of trials gave any justification for their exclusion criteria, yet over half excluded participation for age or mental health reasons. Just over half (58%) of the trials reported participants' race or ethnicity, and a fifth reported socioeconomic status. Where outcomes were reported for under-served groups, the most

For peer review only - http://bmjopen.bmj.com/site/about/guidelines.xhtml

common analysis was by sex (47.3%) followed by race or ethnicity (16.8%). 3.1% of trials reported outcomes according to socioeconomic status.

Discussion

Although we were limited by poor trial reporting, our results indicate inadequate representation of people most at risk of obesity. Guidance for considering under-served groups may improve the appropriateness of research and inform greater engagement with health and social care services.

Funding

National Institute for Health Research Health Technology Assessment Programme (Project number: 15/09/04).

Systematic review registration

PROSPERO CRD42016040190

For peer review only - http://bmjopen.bmj.com/site/about/guidelines.xhtml

ARTICLE SUMMARY

Strengths and limitations of this study

- A unique analysis exploring whether randomised trials evaluating interventions for weight management for people with severe obesity consider the needs of under-served groups
- Data set includes up-to-date, best available randomised controlled trial evidence (considered to be the highest level of evidence to inform guidelines and clinical practice) identified using robust and exhaustive literature search strategies.
- Data set includes wide-ranging weight management interventions delivered in different settings, from an international perspective
- Our analysis was limited by poor reporting of whether subgroup reporting by under-served groups was planned and whether trials were adequately powered to detect subgroup differences
- Non-randomised study designs, interventions with shorter follow-up, and unpublished studies may have been more inclusive in their designs and reporting.

only

For peer review only - http://bmjopen.bmj.com/site/about/guidelines.xhtml

Background

In high-income countries, and increasingly in low and middle-income countries, lower incomes, less education, lower socioeconomic status, and disability are associated with greater risk of obesity for adults.[1-5] While the underlying causes of obesity are varied, there is an increasing association between obesity and deprivation that is driving poorer health outcomes and increasing health inequalities.[6] For example, most of these risk factors are stronger for women than men, [1-3,7] although men with obesity may be less likely than women to undertake weight management programmes.[8] Being an adult with obesity is associated with a lower health-related quality of life than a healthy weight adult of the same socioeconomic status.[9] More severe obesity, such as BMI ≥35kg/m^2 or more, with its associated greater risks for comorbidities, reduced quality of life and premature mortality [10] is particularly related to lower socioeconomic status, [1, 11] and intellectual and physical disabilities.[4] Poorer outcomes from COVID-19 are also strongly related to obesity, particularly severe obesity.[5] For countries such as the US, UK, Australia and New Zealand, some racial or ethnic groups may also be at much greater risk of obesity, especially severe obesity. [11- 14]

Preventing obesity and providing effective interventions, particularly for people with more severe obesity, is, therefore, a major public health challenge and vital in terms of addressing health inequalities. While organisations such as the U.S. Food and Drug Administration (FDA),[15-18] the National Institutes of Health (NIH)[15] and the National Institutes of Health Research (NIHR)[19] have produced guidelines on the inclusion of individuals of all ages, sexes/genders, races and ethnicities, and other physical, sensory/perceptual, cognitive and emotional characteristics, there is a lack of accessible policy-ready evidence on what works in terms of interventions to reduce inequalities in obesity. It is also recognised that some

For peer review only - http://bmjopen.bmj.com/site/about/guidelines.xhtml

groups, (for example, socially disadvantaged, less educated, and minority race or ethnic groups) may be less likely to be recruited into randomised controlled trials (RCTs) for lifestyle interventions.[20-24] Similarly, religion [25] and sexual orientation [26, 27] have been linked to weight and body image. It is, therefore, important to understand the extent to which the current evidence base is applicable to those who are most at risk of experiencing poorer obesity-related health outcomes and have more severe obesity.

This study aimed to determine the extent to which the findings from intervention studies of weight management, as exemplified by long-term randomised controlled trials (RCTs), are generalisable and applicable to those most at risk, particularly under-served groups with severe obesity. To examine these questions, we set out:

1. to describe inclusion and exclusion criteria for RCTs of adult weight management interventions, and in those trials:
2. to describe efforts to tailor recruitment strategies to improve recruitment of people from under-served groups,
3. to describe efforts to culturally adapt interventions to increase the accessibility or appeal to shared characteristics of an under-served group,
4. to describe reported baseline characteristics and outcomes for these groups.

Methods

Our dataset comprised 131 RCTs included in a systematic review of weight management interventions for adults with BMI ≥35kg/m^2, as part of the REBALANCE project (NIHR HTA15/09/04).[10] BMI ≥35kg/m^2 was chosen as this is a cut-off often used for accessing bariatric surgery or secondary care weight management clinical services in the UK. Eligible interventions included diet (including, but not limited to, very low calorie diets and meal

For peer review only - http://bmjopen.bmj.com/site/about/guidelines.xhtml

replacements), lifestyle (including combinations of diet, physical activity, and types of counselling), bariatric surgery, or orlistat. RCTs were restricted to publications after 1990 up to May 2017 to reflect more recent clinical practice. Literature searching was conducted in June 2016 and updated in April/May 2017. Details of the literature search method and search strategy are available in Appendix 1 of the supplementary files. Trials had to report long-term data on weight change (≥ 1 year of follow-up) and include trial populations with a baseline mean or median BMI ≥35kg/m^2. The decision to focus on long-term RCTs for this study was informed by the preference for high-quality, long-term evidence of lasting effectiveness in guideline documents [8, 28- 31] and are, therefore, most likely to influence treatment policy decisions. Reports published as abstracts or conference proceedings only were excluded. Three reviewers screened titles, abstracts and full text reports with a 10% quality assessment check. We attempted to contact the first, second and last authors of the main publications to identify all additional materials (i.e. protocols, trial materials and diet books) to inform our data extraction for the main report. Full details of the REBALANCE project, including the protocol, have been published.[10]

In the absence of definitions of under-served groups, we identified under-served groups by using protected characteristics informed by the PROGRESS-Plus framework[32] and the UK Equality Act 2010.[33] Four reviewers (MA-M, MC, MI and CR) conducted double data coding of each RCT for their reporting of whether trials reported details of their inclusion and exclusion criteria, trial recruitment strategies, baseline characteristics, and outcome reporting for the following characteristic groups, with disagreements resolved by consensus:

- Older age
- Physical health

For peer review only - http://bmjopen.bmj.com/site/about/guidelines.xhtml

- Mental health (including, but not limited to, depression, psychosis, schizophrenia, substance abuse and eating disorders)
- Comorbidities (e.g. Types 1 and 2 diabetes mellitus)
- Gender/sex (including RCTs recruiting only men or women)
- Sexual orientation
- Gender reassignment
- Marriage or civil partnership status
- Pregnancy
- Religion or belief
- Place of residence/housing (including residents of supported accommodation and homeowner status)
- Race or ethnicity
- Language
- Occupational status
- Education/literacy
- Socioeconomic status (SES), including individual SES and participants recruited from rural or disadvantaged geographical locations
- Social capital (including social support networks and/or social isolation)
- PROGRESS Plus (personal characteristics associated with discrimination (e.g. age, disability), features of relationships (e.g. smoking parents, excluded from school), time-dependent relationships (e.g. leaving the hospital, respite care, other instances where a person may be temporarily at a disadvantage)

For inclusion and exclusion criteria, trials were coded by pre-defined categories indicating whether any of the characteristic groups were clearly reported in the inclusion/exclusion

For peer review only - http://bmjopen.bmj.com/site/about/guidelines.xhtml
For peer review only

criteria, or, where details were not reported, whether the setting of the trial encouraged/discouraged inclusion of individuals from a particular characteristic group (e.g. recruitment was set in a health centre predominantly serving people from a characteristic group), or by whether it was unclear that the trial included/excluded people from any of the characteristic groups. For baseline and outcome reporting, we coded whether the protected characteristic was reported and, if reported, whether it was reported for individual treatment groups or the trial population as a whole. Where subgroup analyses were reported, we coded these according to whether it was clear/unclear from the study report that analyses were pre-planned. For a trial to be coded as having adapted their recruitment strategy for an under-served group, additional efforts to employ strategies that would appeal to that particular group (e.g. held recruitment days in particular settings or developed recruitment materials in multiple languages) had to be demonstrated. Trials that solely recruited from one characteristic group using conventional recruitment methods (e.g. newspaper or radio advertisements) were not coded as having an adapted recruitment strategy. Similarly, trials had to demonstrate that their interventions were designed with an under-served group in mind to be coded as having delivered a culturally adapted intervention. The focus of this study was to provide a description of trial methods and trial reporting to answer each of our research questions in relation to under-served groups; therefore, no formal statistical analysis was conducted.

Ethics approval

Ethical approval was not required.

Patient and public involvement

For peer review only - http://bmjopen.bmj.com/site/about/guidelines.xhtml

Although we did not consult patient representatives for this particular analysis, three patient representatives were members of the REBALANCE project Advisory Group, who contributed to developing the research questions, data interpretation and reporting of the research findings.

Results

From the total of 131 included trials, 19 were identified from database searching and 112 were identified from autoalert searching. The PRISMA (Preferred Reporting Items for Systematic Reviews and Meta-Analyses) flow chart and list of included studies are presented in Appendices 2 and 3 of the supplementary files. Of the 131 trials, 41 (31.3%) provided us with additional materials for their publications, although most of the information for the current analysis was obtained from the primary publications. The majority (81/131, 61.8%) of included studies were set in North America (80 in the USA and 1 in the USA and Canada), 41 out of 131 (31.3%) were in European countries (including 8 in the UK), 8 (6.1%) were in the Southern Hemisphere (six in Australia, one in New Zealand and one in Australia and New Zealand) and one in Brazil. None of the trials were set in low-income countries. Just under half (62/131, 47.3%) of the studies were published between 2011 and 2017. Five (3.8%) trials were linked to publications reporting qualitative data.[34-38] Few trials had follow-up durations longer than 12 months, with the exceptions of the US Look AHEAD trial [39] (median duration of 9.6 years), and four trials with follow-up times of five years. [40-43] The Look AHEAD trial was the largest trial, including over 5000 participants. Interventions were wide ranging, including very low calorie (19/131, 14.5%), orlistat (12/131 9.2%), bariatric surgery (11/131, 8.4%) and other lifestyle weight management programmes incorporating diet and physical activity advice (89/131, 67.9%). Details of the characteristics of the included studies can be found in the supplementary files of the REBALANCE report.[10]

For peer review only - http://bmjopen.bmj.com/site/about/guidelines.xhtml

Trial recruitment

More than half of the trials (71/131, 54%) recruited participants either solely or partially through a health service provider, for example, either solely from outpatient clinics and general practices,[44, 45] or by physician referral and targeted mailing.[46] Recruitment methods were unclear or not reported in 14 trials (10.7%).[47-59] Recruitment methods for the other trials were mainly advertisements in local newspapers or other media. Based on their reporting, only three (2.3%) trials were judged to have adapted their recruitment strategies to appeal to under-served groups.[36, 60, 61] These pre-planned strategies included holding pre-recruitment presentations in US schools,[60] recruitment events at football stadiums of Scottish Premier League football clubs [36] and having bilingual staff take informed consent and provide written consent forms in both English and Spanish languages.[61] It was unclear in a further five (3.8%) trials whether recruitment strategies had been adapted beyond conventional methods.[53-55,62, 63]

Regarding adaptions to interventions, seven trials (5.3%, six from the US and one from New Zealand) [61, 63-68] recruited participants from diverse racial or ethnic groups and reported cultural adaptations to their interventions. Five (3.8%) of these trials [63-67] included advice on regional or culturally adapted recipes and foods for specific ethnic groups. Two trials (1.5%) [61, 68] had interventions that were delivered by bilingual staff. One trial [68] also reported that the intervention was designed for delivery in populations with limited literacy and numeracy and impaired access to health-promoting resources. While two trials [52, 69] recruited participants from workplace settings (an automobile manufacturer and a university; both were not considered to meet the PROGRESS-Plus occupation definition), and one trial recruited married participants,[70] the trials did not report any attempts to alter their recruitment

For peer review only - http://bmjopen.bmj.com/site/about/guidelines.xhtml

strategies or interventions to appeal to under-served occupation or social capital groups. The trials did not report recruitment strategies or adaptions to interventions for sexual orientation.

A further five trials (all from the US) [71-75] sought recruitment from specific racial or ethnic groups and reported intervention adaptations to increase cultural salience. Four of these trials [71-74] included culturally specific dietary advice and recipes. One trial described including bilingual interventions [71] and three trials described including interventionists from specific racial or ethnic groups.[75,73,74] Two trials [73, 74] described using logos and programme identification "for African-Americans", with one of these trials [73] including a video greeting from an African-American principal investigator.

The number of trials reporting inclusion and exclusion criteria by protected characteristic groups is presented in Table 1. Four older trials (3.1%) [47, 52, 76,77] did not report any inclusion criteria, and inclusion criteria was unclear in one further study.[42] Seven (5.3%) [52, 70, 78-82] trials did not report any explicit exclusion criteria and did not report that they had no exclusion criteria. Eight (6.1%) trials reported either full [36, 38, 45, 72, 83, 84] or partial [60, 85] justification for their exclusion criteria. Justification for exclusion criteria included prevention of poor adherence and losses to follow-up, [38, 45, 84, 85] such as substance abuse, mental health problems, or cognitive impairment (that might, in the opinion of the investigators, hinder participation), lower BMI cut-offs for Asian people,[60] non-English language speakers where the intervention required English language comprehension,[84] influence of pregnancy and breastfeeding on weight,[84] taking medications that influence weight,[72] and contraindications or safety concerns associated with participating in the intervention (e.g. risk of participants with cardiovascular disease participating in exercise programmes). [36, 45, 84, 85] Over half (58.0%) of the trials reported excluding people with

For peer review only - http://bmjopen.bmj.com/site/about/guidelines.xhtml

mental health conditions and 44.2% excluded people with substance abuse or addiction issues. The majority of trials also excluded adults from older age groups and based on current or planned pregnancy.

Twenty-one (16.0%) trials were judged to have inclusion criteria that might have implicitly excluded certain disadvantaged groups, such as people who do not have healthcare insurance,[69, 79] people who do not belong to a particular religious community group,[87] people without regular internet [35] or telephone [88] access, and English language comprehension.[44, 45, 49, 56, 60, 68, 89-96] In a further 40 (30.5%) trials, it was unclear if trial recruitment could have implicitly excluded disadvantaged groups. Few trials reported their inclusion criteria so as to include particular under-served groups or were judged to have made efforts to maximise trial recruitment from these groups. When trial recruitment was targeted, this was usually to recruit women only (19 trials) or people with Type 2 diabetes (28 trials).

For peer review only

Table 1 The number (and percent) of trials (n=131) reporting inclusion and exclusion criteria by protected characteristics included in the REBALANCE systematic review of RCTs

	Inclusion			Exclusion
	Protected characteristic is reported in the inclusion criteria, n (%)	Protected characteristic is not reported in inclusion criteria, but an effort was made to recruit from the protected characteristic group, n (%)	Unclear if the protected characteristic was targeted for inclusion or if the trial unintentionally recruited solely/mainly from the protected characteristic group, n (%)	Protected characteristic is reported in the exclusion criteria, or the reported inclusion criteria clearly excluded the protected characteristic, n (%)
Place of residence/housing	5 (3.8%)	0	0	0
Race/ethnicity	7 (5.3%)	5 (3.8%)	2 (1.5%)	1 (0.8%)
Occupation status	0	0	0	0
Women only	19 (14.5%)	0	4 (3.1%)	0
Pregnancy	0	0	0	72 (54.9%)
Men only	4 (3.0%)	0	1 (0.76%)	5 (3.8%)
Religion/belief	1 (0.8%)			
Education/literacy	0	0	0	0
Socioeconomic status	3 (2.3%)	3 (2.3%)	0	0
Marital status	1 (0.8%)	0	0	1 (0. 8%)
Older age	2 (1.5%)	1 (0.8%)	0	82 (62.6%)[a]
Physical health	10 (7.6%)	0	0	51 (38.9%)
Diabetes Type 1	0	0	0	15 (11.5%)
Diabetes Type 2	28 (21.4%)	0	0	29 (22.1%)
Diabetes (Type 1 and 2 or Type not reported)	0	0	0	3 (2.3%)
Mental health	6 (4.6%)	0	0	76 (58.0%)
Substance abuse	0	0	0	58 (44.2%)
Eating disorder	0	0	0	35 (26.7%)
Language	0	0	0	16 (12.2%)

a. includes 8 RCTs recruiting participants up to 75 years, 1 RCT recruited participants up to 76 years and 3 RCTs recruited participants aged up to 80 years

For peer review only

Reporting of baseline characteristics

Details of the number of trials reporting baseline characteristics of their participants by each of the protected characteristic groups are presented in Table 2. The majority of trials reported age (99.2%) and sex (97.7%) in their description of baseline participant characteristics. Just over half of the trials (58.0%) reported race or ethnicity. Education history was less well-reported (40.5%). Socioeconomic status was reported by 22.9%, and occupation status by 21.4%. Of the trials that were not specifically for people with diabetes, six (4.6%) included diabetes in their reporting of baseline characteristics. Two (1.5%) trials reported whether people lived alone or not (coded as social capital).[97, 98] Few trials reported details of the other protected characteristics, and none reported details of gender reassignment, sexual orientation or pregnancy.

For peer review only - http://bmjopen.bmj.com/site/about/guidelines.xhtml

Table 2 The number (and percent) of trials (n=131) reporting protected characteristics at baseline in the REBALANCE systematic review of RCTs

	Protected characteristic is reported at baseline for each intervention arm	Protected characteristic is reported at baseline for the whole trial	Total
Age	126 (96.2%)	4 (3.0%)	130 (99.2%)
Physical health	10 (7.6%)	-	10 (7.6%)
Mental health	10 (7.6%)	-	10 (7.6%)
Diabetes	6 (4.6%)	-	6 (4.6%)
Sex	126 (96.2%)	2 (1.5%)	128 (97.7%)
Gender reassignment	0	0	0
Sexual orientation	0	0	0
Marriage/civil partnership status	38 (29.0%)	-	38 (29.0%)
Pregnancy	0	0	0
Place of residence/housing	6 (4.6%)	-	6 (4.6%)
Occupation status	27 (20.6%)	1 (0.8%)	28 (21.4%)
Education/literacy	51 (38.9%)	2 (1.5%)	53 (40.5%)
Socioeconomic status	29 (22.1%)	1 (0.8%)	30 (22.9%)
Social capital	2 (1.5%)	-	2 (1.5%)
Religion/belief	1 (0.8%)	-	1 (0.8%)
Race/ethnicity	74 (56.5%)	2 (1.5%)	76 (58.0%)
PROGRESS Plus	2 (1.5%)	-	2 (1.5%)

For peer review only

Outcome reporting

Details of the number of trials reporting outcomes by each of the protected characteristics are shown in Table 3. Very few trials reported outcomes by protected characteristic groups. Where outcomes were reported by protected characteristics, the most common group was sex (47.3%) followed by race or ethnicity (16.8%).

For peer review only

Table 3 The number (and percent) of trials (n=131) reporting outcome data for protected characteristics in the REBALANCE systematic review of RCTs

	Trial recruitment was targeted at people from the protected characteristic group	One or more outcome(s) reported for the protected characteristic in planned subgroup analysis	One or more outcome(s) reported for the protected characteristic – unclear if subgroup analysis was pre-planned	Total
Older Age	2 (1.5%)[a]	2 (1.5%)	3 (2.3%)	7 (5.3%)
Physical health	10 (7.6%)	0	0	10 (7.6%)
Mental health	6 (4.6%)	0	2 (1.5%)	8 (6.1%)
Diabetes	28 (21.3%)	0	1 (0.8%)	29 (22.1%)
Sex	23 (17.5%) [b]	17 (13.0%)	22 (16.8%)	62 (47.3%)
Gender reassignment	0	0	0	0
Sexual orientation	0	0	0	0
Marriage/civil partnership status	1 (0.8%)	1 (0.8%)	3 (2.3%)	5 (3.8%)
Pregnancy	0	0	0	0
Place of residence/housing	0	2 (1.5%)	0	2 (1.5%)
Occupation status	2 (1.5%)	2 (1.5%)	4 (3.1%)	8 (6.1%)
Education/literacy	0	1 (0.8%)	6 (4.6%)	7 (5.3%)
Socioeconomic status	5 (3.8%)	4 (3.1%)	3 (2.3%)	12 (9.2%)
Social capital	1 (0.8%)	3 (2.3%)	2 (1.5%)	6 (4.6%)
Religion/belief	1 (0.8%)	0	0	1 (0.8%)
Race/ethnicity	8 (6.1%)	5 (3.8%)	9 (6.9%)	22 (16.8%)
PROGRESS Plus	0	0	0	0

a. Both trials recruited participants aged ≥65 years
b. 19 women only trials, 4 men only trials

For peer review only

Discussion

Main findings

Our findings demonstrate that most trialists testing weight management strategies to help adults with severe obesity fail to consider populations who are most at risk of poorer health outcomes. Almost all trials were from high-income countries, where lower socioeconomic status and income are associated with a greater prevalence of obesity, particularly severe obesity.[1-5] Few trials reported adapting recruitment to appeal to under-served groups, or reported culturally adapting their trial materials for ethnic groups or people with limited English language literacy or numeracy. This is concerning as limiting the accessibility or appeal of trials could limit the representativeness of the trial population, and thus limit the generalisability of trial findings. Only 6.1% of trials gave any justification for their exclusion criteria, yet more than half excluded participation for age or mental health reasons. Where justification for exclusion was reported, the rationale included excluding people who were deemed likely to have poor intervention adherence or were more likely to be lost to follow-up, such as people with substance abuse, cognitive impairment, or mental health problems. Excluding these groups could lead to an unrealistic estimation of the real-world effectiveness of interventions. Just over half of the trials reported participants' race or ethnicity, and only around a fifth reported socioeconomic status. Where outcomes were reported for under-served groups, the most common analysis was by sex (47.3%) followed by race or ethnicity (16.8%); however, where analyses were presented as subgroups, it was often unclear whether these analyses were planned or unplanned. Similarly, some smaller trials might have been underpowered to detect differences in treatment effects between subgroups, but this was also unclear from trial reporting. This finding was also demonstrated by Liu and colleagues (2020),[99] who highlighted a lack of transparent reporting of intentions to analyse race and ethnicity subgroups in Cochrane intervention reviews. Only 3.1% of the trials we reviewed

For peer review only - http://bmjopen.bmj.com/site/about/guidelines.xhtml

reported outcomes according to socioeconomic status. Few trials reported outcomes by the other protected characteristics.

Although we were limited by the available information in the published reports,, our findings are concerning. In almost all trials, it would be impossible to assess the generalisability of findings to the wider population of adults with severe obesity. There is clear evidence [1-5,11,12] that under-served groups with lower incomes, less education, lower socioeconomic status, intellectual and physical disabilities, or poorer mental health are more at risk of obesity, particularly severe obesity, in high-income countries, especially the US which provided the majority of trials examined. We do not have relevant data to be able to comment on the reasons for poor reporting. Nevertheless, the lack of reporting for characteristics reflecting under-served groups suggests that trial investigators did not consider, or faced barriers that prevented their inclusion in the design, recruitment, and analysis or reporting of their interventions.

Our finding that few trials adapted their recruitment methods or interventions to appeal to under-served groups suggests lack of engagement with under-served people with obesity in the design of services. This is important, given that a systematic review of qualitative research by Sutcliffe and colleagues [100] showed how service users have perspectives that should inform weight management services to improve their reach. From systematic reviews, researchers have clearly demonstrated the need to involve communities in all stages of research in order to enhance the engagement and generalisability of that research, acknowledging that this requires extended timeframes and greater costs.[21, 101] For example, Ni She and colleagues (2018) [102] undertook a rapid realist review of the mechanisms and resources needed to engage under-served, seldom-heard groups in health and social care

For peer review only - http://bmjopen.bmj.com/site/about/guidelines.xhtml

research, with items grouped by an expert panel under the headings of environmental and social planning, service provision, guidelines, fiscal measures, communication and marketing, and regulation and legislation. In the US, Arnegard and colleagues (2020)[103] have also called on the NIH's stakeholder groups to redouble their efforts to encourage sex- and gender-aware reporting of biomedical investigations. We endorse this call following the findings from our previous systematic review of weight management interventions for men with obesity.[8] Our review highlighted the paucity of evidence for men, who are less likely to take part in weight management interventions and the lack of engagement of men in all aspects of intervention design, and optimal trial recruitment processes of weight management. [8]

While the reasons for the under-representation of under-served groups in RCTs are likely to be complex and multifaceted, with many known and unknown barriers to participation, there is evidence that, for some groups, willingness to participate is not a predominant factor.[104] Mindful of the need to improve the engagement of under-served groups in research in the UK, the National Institute for Health Research set up the INCLUDE project, [19] which has led to the INCLUDE ethnicity framework; [105] providing four key questions on who should be involved in research, and how to facilitate involvement. Others have investigated and found a lack of external validity in trials for people with asthma,[106] type 2 diabetes,[107, 108] and neurological disease,[109] or a failure to justify exclusion criteria in trials of cardiovascular disease prevention [110] other than for safety reasons. These publications did not consider the socioeconomic status or educational attainment of trial participants. In a systematic review of 305 trials of clinical conditions, He and colleagues [111] found high exclusion rates in trials for people with hypertension (83.0%), lipid-lowering drugs in primary prevention (85.9%), type

For peer review only - http://bmjopen.bmj.com/site/about/guidelines.xhtml

2 diabetes (81.7%), COPD (84.3%) and asthma (96.0%), with no strong evidence that exclusion rates had changed with time.

More recently, others have also highlighted the small number of intervention studies testing out weight management for under-served adults,[112] or policies to assist socioeconomically disadvantaged groups. [113] A 2015 systematic review of interventions aimed at reducing socioeconomic inequalities for adults with obesity [114] found that primary care-delivered tailored weight loss programmes and group weight loss interventions had the most evidence of potential effectiveness in reducing obesity, at least in the short term, among low-income women, but there were few individual-level intervention studies and a lack of long term evidence of effectiveness.

Strengths and limitations

We used categories informed by the PROGRESS-PLUS framework [32] and the UK Equality Act 2010 [33] as the key characteristics for identifying those under-served participants that should be considered for study design, public patient involvement, recruitment, analysis and reporting, not just for trials of weight management, but trials generally. O'Neill (2014)[32] has shown how the prior PROGRESS framework can be used as an equity lens for systematic reviews and methodological studies; however, NIHR's INCLUDE project has recently published a more extensive list of categories of under-served groups to consider with regard to representation in trials.[115]

Our literature search attempted to identify all long-term randomised trials published since 1990 for adults with BMI ≥35kg/m^2 irrespective of the type of lifestyle intervention, including comparisons with orlistat and bariatric surgery. Although we included publications

For peer review only - http://bmjopen.bmj.com/site/about/guidelines.xhtml

in any language from any country, we cannot exclude the possibility that we failed to find some trials, particularly those from low income countries, which might not be listed in the databases we searched.

We did not re-contact authors for the additional information extracted for this analysis and were limited by poor reporting by trial authors. Some trials were statistically underpowered to detect subgroup differences, and this might explain under-reporting of under-served characteristics; however, this was unclear from the trial reports. It is also possible that some trialists were unable to obtain relevant baseline data for some under-served groups if this was deemed sensitive by an ethics committee, e.g. sexual orientation. Nevertheless, we consider that most characteristics are pivotal to interpreting these trials into real-world guidance and services, so we would expect their presentation in trial publications, especially at baseline. We assessed long-term RCT evidence because it is most likely to inform guidance on weight management.[28-31] Other study designs, interventions with shorter follow-up, and unpublished studies may have been more inclusive in their designs and reporting.

Recommendations for research

Trialists should improve reporting of their justification of inclusion and exclusion criteria to meet current CONSORT statement guidelines,[116] and report sufficient data to allow comparisons between their populations and the populations for whom the interventions apply. Including core criteria for baseline reporting within the CONSORT checklist [116] could help to improve the completeness of reporting of these factors. NIHR's INCLUDE project's ethnicity framework provides important factors to consider with regard to ethnic groups (https://www.trialforge.org/trial-forge-centre/include/), which can help provide transferable considerations for other under-served groups. However, a wider equity lens may be needed in

For peer review only - http://bmjopen.bmj.com/site/about/guidelines.xhtml

the face of groups with multiple disadvantages. Although guidance for research will aid considerations of equity, we do not yet have ways of assessing when proportional representation in larger trials and sub-group reporting for under-served groups is insufficient. This research should be explicitly conducted with and for these under-served groups, ensuring user involvement in all stages of the research process.

Conclusions and recommendations for practice

Long-term RCTs of weight management in people with BMI ≥35mg^2 lack generalisability and engagement with under-served groups, who are particularly relevant for health and social care services. Thus, guidance for weight management research on how to improve the representation of under-served groups in clinical trials may improve the appropriateness of that research and help inform greater engagement of under-served communities with health and social care services.

For peer review only

REFERENCES

1. Booth HP, Charlton J, Gulliford MC. Socioeconomic inequality in morbid obesity with body mass index more than 40 kg/m^2 in the United States and England. *SSM Popul Health* 2017;3:172–178
2. Devaux M, Sassi F. Social inequalities in obesity and overweight in 11 OECD countries. *Eur J Public Health* 2013; 23(3):464–469
3. El-Sayed AM, Scarborough P, Galea S. Unevenly distributed: a systematic review of the health literature about socioeconomic inequalities in adult obesity in the United Kingdom. *BMC Public Health* 2012, 12:18
4. Hsieh K, Rimmer JH, Heller T. Obesity and associated factors in adults with intellectual disability. *J Intellect Disabil Res* 2014;58(9):851-863
5. Public Health England. Excess Weight and COVID-19 Insights from new evidence [Internet]. London: PHE publications; 2021 [cited February 2021]. Available from: https://assets.publishing.service.gov.uk/government/uploads/system/uploads/attachment_data/file/907966/PHE_insight_Excess_weight_and_COVID-19__FINAL.pdf
6. Holmes J. Tackling obesity: The role of the NHS in a whole-system approach. London: The Kings Fund 2021.
7. Grech A, Allman-Farinelli M. Prevalence and period trends of overweight and obesity in Australian young adults. *Eur J Clin Nutr* 2016; 70: 1083–1085
8. Robertson C, Archibald D, Avenell A, et al. Systematic reviews of and integrated report on the quantitative, qualitative and economic evidence base for the management of obesity in men. *Health Technol Assess* 2014;18(35).
9. Minet Kinge J, Morris S. Socioeconomic variation in the impact of obesity on health-related quality of life. *Soc Sci Med* 2010;71:1864e1871

For peer review only - http://bmjopen.bmj.com/site/about/guidelines.xhtml

10. Avenell A, Robertson C, Skea Z, et al. Bariatric surgery, lifestyle interventions and orlistat for severe obesity: the REBALANCE mixed-methods systematic review and economic evaluation. *Health Technol Assess* 2018;22(68).

11. Green MA, Rowe F. Explaining the widening distribution of Body Mass Index: A decomposition analysis of trends for England, 2002–2004 and 2012–2014. *Area* 2020;1–11

12. Ogden CL, Fryar CD, Martin CB, et al. Trends in Obesity Prevalence by Race and Hispanic Origin—1999-2000 to 2017-2018. *JAMA* 2020;324(12):1208–1210

13. Menigoz, K., Nathan, A. & Turrell, G. Ethnic differences in overweight and obesity and the influence of acculturation on immigrant bodyweight: evidence from a national sample of Australian adults. *BMC Public Health* 2016; 16, 932

14. New Zealand Ministry of Health. Obesity statistics. 2020. Available from https://www.health.govt.nz/nz-health-statistics/health-statistics-and-data-sets/obesity-statistics (Accessed October 2021)

15. U.S.Food and Drug Administration (FDA). Guidance for industry: collection of race and ethnicity data in clinical trials. https://www.fda.gov/media/75453/download Accessed March 2021

16. U.S.Food and Drug Administration (FDA). Evaluation and reporting of age-, race-, and ethnicity-specific data in medical device clinical studies. https://www.fda.gov/media/98686/download Accessed March 2021

17. U.S.Food and Drug Administration (FDA). Design considerations for devices intended for home use. https://www.fda.gov/media/84830/download Accessed March 2021

For peer review only - http://bmjopen.bmj.com/site/about/guidelines.xhtml

18. National Institutes of Health [internet]. U.S. 2019. Inclusion policies for research involving human subjects. https://grants.nih.gov/policy/inclusion.htm (accessed March 2021)

19. National Institute for Health Research [Internet]. London: The Institute; 2020 [cited February 2021]. Improving inclusion of under-served groups in clinical research: Guidance from the NIHR INCLUDE project. Available from: www.nihr.ac.uk/documents/improving-inclusion-of-under-served-groups-in-clinical-research-guidance-from-include-project/25435

20. Unger JM, Gralow JR, Albain KS, Ramsey SD, Hershman DL. Patient Income Level and Cancer Clinical Trial Participation: A Prospective Survey Study. *JAMA Oncol*. 2016;2(1):137-9.

21. Bonevski, B., Randell, M., Paul, C. et al. Reaching the hard-to-reach: a systematic review of strategies for improving health and medical research with socially disadvantaged groups. *BMC Med Res Methodol* 2014;14:42

22. Sateren W.B., Trimble E.L., Abrams J. et al. How Sociodemographics, Presence of Oncology Specialists, and Hospital Cancer Programs Affect Accrual to Cancer Treatment Trials. *J Clin Oncol* 2002 20:8, 2109-2117

23. Smart, A., Harrison, E. Under¬representation of minority ethnic groups in UK medical research. *Ethnicity and Health* 2017, 22: 65–82

24. Sardar MR, Badri M, Prince CT, Seltzer J, Kowey PR. Underrepresentation of Women, Elderly Patients, and Racial Minorities in the Randomized Trials Used for Cardiovascular Guidelines. *JAMA Intern Med*. 2014;174(11):1868–1870

25. Yeary, K. H. K., Sobal, J., and Wethington, E. Religion and body weight: a review of quantitative studies. *Obes Rev* 2017, 18: 1210– 122

For peer review only - http://bmjopen.bmj.com/site/about/guidelines.xhtml

26. de Souza P, Ciclitira KE. Men and Dieting: A Qualitative Analysis. *J Health Psychol* 2005;10(6):793-804

27. Gough B, Flanders G. Celebrating 'obese' bodies: Gay 'bears' talk about weight, body image and health. *Int J Mens Health* 2009;8:235–53

28. National Institute for Health and Care Excellence. Weight management: lifestyle services for overweight or obese adults. London; NICE 2014. URL https://www.nice.org.uk/guidance/ph53 (accessed May 2021)

29. National Health and Medical Research Council. Clinical practice guidelines for the management of overweight and obesity in adults, adolescents and children in Australia. Melbourne: National Health and Medical Research Council 2013

30. Hartmann-Boyce J, Johns D, Aveyard P, et al. Managing overweight and obese adults: update review. The clinical effectiveness of long-term weight management schemes for adults: Review 1a. Oxford: University of Oxford 2013

31. Hartmann-Boyce J, Johns D, Aveyard P, et al. How components of behavioural weight management programmes affect weight change: Review 1b. Oxford: University of Oxford 2013

32. O'Neill J, Tabish H, Welch V, et al. Applying an equity lens to interventions: using PROGRESS ensures consideration of socially stratifying factors to illuminate inequities in health. *J Clin Epidemiol* 2014, 67(1):56-64

33. Government Equalities Office, Equality and Human Rights Commission [Internet]. London: Government Digital Service; 2013 [cited February 2021]. Equality Act 2010: guidance. Available from: https://www.gov.uk/guidance/equality-act-2010-guidance

34. Yeh MC, Rodriguez E, Nawaz H, et al. Technical skills for weight loss: 2-y follow-up results of a randomized trial. *Int J Obes Relat Metab Disord* 2003;27:1500–6

For peer review only

For peer review only - http://bmjopen.bmj.com/site/about/guidelines.xhtml

35. Little P, Stuart B, Hobbs FR, et al. Randomised controlled trial and economic analysis of an internet-based weight management programme: POWeR+ (Positive Online Weight Reduction). *Health Technol Assess* 2017;21(4)

36. Hunt K, Gray CM, Maclean A, Smillie S, Bunn C, Wyke S. Do weight management programmes delivered at professional football clubs attract and engage high risk men? A mixed-methods study. *BMC Public Health* 2014;14:50

37. McRobbie H, Hajek P, Peerbux S, et al. Tackling obesity in areas of high social deprivation: clinical effectiveness and cost-effectiveness of a task-based weight management group programme – a randomised controlled trial and economic evaluation. *Health Technol Assess* 2016;20(79)

38. Green CA, Yarborough BJ, Leo MC, et al. Weight maintenance following the STRIDE lifestyle intervention for individuals taking antipsychotic medications. *Obesity* 2015;23:1995–2001

39. The Look AHEAD Research Group. Cardiovascular effects of intensive lifestyle intervention in type 2 diabetes. *N Engl J Med* 2013;369:145–54

40. Hakala P, Karvetti RL, Rönnemaa T. Group vs. individual weight reduction programmes in the treatment of severe obesity – a five year follow-up study. I*nt J Obes Relat Metab Disord* 1993;17:97–102

41. Hakala P. Weight reduction programmes at a rehabilitation centre and a health centre based on group counselling and individual support: short- and long-term follow-up study. *Int J Obes Relat Metab Disord* 1994;18:483–9

42. Mingrone G, Greco AV, Giancaterini A, Scarfone A, Castagneto M, Pugeat M. Sex hormone binding globulin levels and cardiovascular risk factors in morbidly obese subjects before and after weight reduction induced by diet or malabsorptive surgery. *Atherosclerosis* 2002;161:455–62

For peer review only - http://bmjopen.bmj.com/site/about/guidelines.xhtml

43. Schauer PR, Kashyap SR, Wolski K, Brethauer SA, Kirwan JP, Pothier CE, et al. Bariatric surgery versus intensive medical therapy in obese patients with diabetes. *N Engl J Med 2012*;366:1567–76

44. Bliddal H, Leeds AR, Stigsgaard L, Astrup A, Christensen R. Weight loss as treatment for knee osteoarthritis symptoms in obese patients: 1-year results from a randomised controlled trial. *Ann Rheum Dis* 2011;70:1798–803

45. Azar KM, Xiao L, Ma J. Baseline obesity status modifies effectiveness of adapted diabetes prevention program lifestyle interventions for weight management in primary care. *Biomed Res Int* 2013;2013:191209

46. Appel LJ, Clark JM, Yeh HC, Wang NY, Coughlin JW, Daumit G, et al. Comparative effectiveness of weight-loss interventions in clinical practice. *N Engl J Med* 2011;365:1959–68.

47. Agras WS, Berkowitz RI, Arnow BA, et al. Maintenance following a very-low-calorie diet. *J Consult Clin Psychol* 1996;64:610–13

48. Bakris G, Calhoun D, Egan B, Hellmann C, Dolker M, Kingma I, Orlistat and Resistant Hypertension Investigators. Orlistat improves blood pressure control in obese subjects with treated but inadequately controlled hypertension. *J Hypertens* 2002;20:2257–67

49. Bartels SJ, Pratt SI, Aschbrenner KA, et al. Clinically significant improved fitness and weight loss among overweight persons with serious mental illness. *Psychiatr Serv* 2013;64:729–36

50. Brehm BJ, Lattin BL, Summer SS, Boback JA, Gilchrist GM, Jandacek RJ, D'Alessio DA. One-year comparison of a high-monounsaturated fat diet with a high-carbohydrate diet in type 2 diabetes. *Diabetes Care* 2009;32:215–20

For peer review only - http://bmjopen.bmj.com/site/about/guidelines.xhtml

51. Broom I, Wilding J, Stott P, Myers N, UK Multimorbidity Study Group. Randomised trial of the effect of orlistat on body weight and cardiovascular disease risk profile in obese patients: UK Multimorbidity Study. *Int J Clin Pract* 2002;56:494–9

52. Dennison KF, Galante D, Dennison D, Golaszewski T. A one year post-program assessment of a computer-assisted instruction weight management program for industrial employees: lessons learned. *J Health Educ* 1996;27:38–43

53. Hauptman J, Lucas C, Boldrin MN, Collins H, Segal KR. Orlistat in the long-term treatment of obesity in primary care settings. *Arch Fam Med* 2000;9:160–7

54. Kahleova H, Hill M, Pelikanova T. Vegetarian vs. conventional diabetic diet – a 1-year follow-up. *Cor Vasa* 2014;56:e140–4

55. Kelley DE, Bray GA, Pi-Sunyer FX, et al. Clinical efficacy of orlistat therapy in overweight and obese patients with insulin-treated type 2 diabetes: a 1-year randomized controlled trial. *Diabetes Care* 2002;25:1033–41

56. Ströbl V, Knisel W, Landgraf U, Faller H. A combined planning and telephone aftercare intervention for obese patients: effects on physical activity and body weight after one year. *J Rehabil Med* 2013;45:198–205

57. Swinburn BA, Carey D, Hills AP, Hooper M, Marks S, Proietto J, et al. Effect of orlistat on cardiovascular disease risk in obese adults. *Diabetes Obes Metab* 2005;7:254–62

58. Wadden TA, Volger S, Sarwer DB, Vetter ML, Tsai AG, Berkowitz RI, et al. A two-year randomized trial of obesity treatment in primary care practice. *N Engl J Med* 2011;365:1969–79

59. Wing RR, Marcus MD, Salata R, Epstein LH, Miaskiewicz S, Blair EH. Effects of a very-low-calorie diet on long-term glycemic control in obese type 2 diabetic subjects. *Arch Intern Med* 1991;151:1334–40

For peer review only

For peer review only - http://bmjopen.bmj.com/site/about/guidelines.xhtml

60. Berry DC, Schwartz TA, McMurray RG, et al. The family partners for health study: a cluster randomized controlled trial for child and parent weight management. *Nutr Diabetes* 2014;4:e101

61. Van Name MA, Camp AW, Magenheimer EA, et al. Effective translation of an intensive lifestyle intervention for Hispanic women with prediabetes in a community health center setting. *Diabetes Care* 2016;39:525–31

62. Khoo J, Piantadosi C, Duncan R, Worthley SG, Jenkins A, Noakes M, et al. Comparing effects of a low-energy diet and a high-protein low-fat diet on sexual and endothelial function, urinary tract symptoms, and inflammation in obese diabetic men. J Sex Med 2011;8:2868–75

63. Kumanyika SK, Fassbender JE, Sarwer DB, et al. One-year results of the Think Health! study of weight management in primary care practices. *Obesity* 2012;20:1249–57

64. Krebs JD, Elley CR, Parry-Strong A, et al. The Diabetes Excess Weight Loss (DEWL) Trial: a randomised controlled trial of high-protein versus high-carbohydrate diets over 2 years in type 2 diabetes. *Diabetologia* 2012;55:905–14

65. Mayer-Davis EJ, D'Antonio AM, Smith SM, et al. Pounds off with empowerment (POWER): a clinical trial of weight management strategies for black and white adults with diabetes who live in medically underserved rural communities. *Am J Public Health* 2004;94:1736–42

66. Perri MG, Limacher MC, Durning PE, et al. Extended-care programs for weight management in rural communities: the Treatment of Obesity in Underserved Rural Settings (TOURS) randomized trial. *Arch Intern Med* 2008;168:2347–54

For peer review only

For peer review only - http://bmjopen.bmj.com/site/about/guidelines.xhtml

67. Perri MG, Limacher MC, von Castel-Roberts K, et al. Comparative effectiveness of three doses of weight-loss counseling: two-year findings from the rural LITE trial. *Obesity* 2014;22:2293–300

68. Bennett GG, Warner ET, Glasgow RE, et al. Obesity treatment for socioeconomically disadvantaged patients in primary care practice. *Arch Intern Med* 2012;172:565–74

69. Ostbye T, Stroo M, Brouwer RJ, et al. Steps to Health employee weight management randomized control trial: Short-term follow-up results. *J Occup Environ Med* 2015;57:188-95

70. Wing RR, Marcus MD, Epstein LH, Jawad A. A 'family-based' approach to the treatment of obese type II diabetic patients. J Consult Clin Psychol 1991;59:156–62

71. Poston WS, Reeves RS, Haddock CK, et al. Weight loss in obese Mexican Americans treated for 1-year with orlistat and lifestyle modification. *Int J Obes Relat Metab Disord* 2003;27:1486–93

72. Martin PD, Dutton GR, Rhode PC, Horswell RL, Ryan DH, Brantley PJ. Weight loss maintenance following a primary care intervention for low-income minority women. *Obesity* 2008;16:2462–7

73. Kumanyika SK, Shults J, Fassbender J, et al. Outpatient weight management in African-Americans: the Healthy Eating and Lifestyle Program (HELP) study. *Prev Med* 2005;41:488–502

74. Kumanyika SK, Wadden TA, Shults J, et al. Trial of family and friend support for weight loss in African American adults. *Arch Intern Med* 2009;169:1795–804

75. Fitzgibbon ML, Stolley MR, Schiffer L, Sharp LK, Singh V, Dyer A. Obesity Reduction Black Intervention Trial (ORBIT): 18-month results. *Obesity* 2010;18:2317–25

For peer review only

For peer review only - http://bmjopen.bmj.com/site/about/guidelines.xhtml

76. Wadden TA, Berkowitz RI, Vogt RA, Steen SN, Stunkard AJ, Foster GD. Lifestyle modification in the pharmacologic treatment of obesity: a pilot investigation of a potential primary care approach. *Obes Res* 1997;5:218–26

77. Wadden TA, Vogt RA, Foster GD, Anderson DA. Exercise and the maintenance of weight loss: 1-year follow-up of a controlled clinical trial. *J Consult Clin Psychol* 1998;66:429–33

78. Torgerson JS, Lissner L, Lindroos AK, Kruijer H, Sjöström L. VLCD plus dietary and behavioural support versus support alone in the treatment of severe obesity. A randomised two-year clinical trial. *Int J Obes Relat Metab Disord* 1997;21:987–94

79. Melin I, Karlström B, Lappalainen R, Berglund L, Mohsen R, Vessby B. A programme of behaviour modification and nutrition counselling in the treatment of obesity: a randomised 2-y clinical trial. *Int J Obes Relat Metab Disord* 2003;27:1127–35

80. Pascale RW, Wing RR, Butler BA, Mullen M, Bononi P. Effects of a behavioral weight loss program stressing calorie restriction versus calorie plus fat restriction in obese individuals with NIDDM or a family history of diabetes. *Diabetes Care* 1995;18:1241–8

81. Perri MG, Nezu AM, McKelvey WF, Shermer RL, Renjilian DA, Viegener BJ. Relapse prevention training and problem-solving therapy in the long-term management of obesity. *J Consult Clin Psychol* 2001;69:722–6

82. Richelsen B, Tonstad S, Rössner S, et al. Effect of orlistat on weight regain and cardiovascular risk factors following a very-low-energy diet in abdominally obese patients: a 3-year randomized, placebo-controlled study. *Diabetes Care* 2007;30:27–32

For peer review only

For peer review only - http://bmjopen.bmj.com/site/about/guidelines.xhtml

83. Cummings DE, Arterburn DE, Westbrook EO, et al. Gastric bypass surgery vs intensive lifestyle and medical intervention for type 2 diabetes: the CROSSROADS randomised controlled trial. *Diabetologia* 2016;59:945–53
84. Daubenmier J, Moran PJ, Kristeller J, et al. Effects of a mindfulness-based weight loss intervention in adults with obesity: a randomized clinical trial. *Obesity* 2016;24:794–804
85. Dixon JB, Schachter LM, O'Brien PE, Jones K, Grima M, Lambert G, et al. Surgical vs. conventional therapy for weight loss treatment of obstructive sleep apnea: a randomized controlled trial. JAMA 2012;308:1142–9
86. Dutton GR, Nackers LM, Dubyak PJ, et al. A randomized trial comparing weight loss treatment delivered in large versus small groups. *Int J Behav Nutr Phys Act* 2014;11:123
87. Latner JD, Ciao AC, Wendicke AU, Murakami JM, Durso LE. Community-based behavioural weight-loss treatment: long-term maintenance of weight loss, physiological, and psychological outcomes. *Behav Res Ther* 2013;51:451–9
88. Lowe MR, Butryn ML, Thomas JG, Coletta M. Meal replacements, reduced energy density eating, and weight loss maintenance in primary care patients: a randomized controlled trial. *Obesity* 2014;22:94–100
89. Christensen P, Frederiksen R, Bliddal H, et al. Comparison of three weight maintenance programs on cardiovascular risk, bone and vitamins in sedentary older adults. *Obesity* 2013;21:1982–90
90. Damschroder LJ, Lutes LD, Kirsh S, et al. Small-changes obesity treatment among veterans: 12-month outcomes. *Am J Prev Med* 2014;47:541–53

For peer review only - http://bmjopen.bmj.com/site/about/guidelines.xhtml

91. Eaton CB, Hartman SJ, Perzanowski E, et al. A randomized clinical trial of a tailored lifestyle intervention for obese, sedentary, primary care patients. *Ann Fam Med* 2016;14:311–19

92. Wolf AM, Conaway MR, Crowther JQ, et al. Improving Control with Activity and Nutrition (ICAN) Study. Translating lifestyle intervention to practice in obese patients with type 2 diabetes: Improving Control with Activity and Nutrition (ICAN) study. *Diabetes Care* 2004;27:1570–6

93. Mensinger JL, Calogero RM, Stranges S, Tylka TL. A weight-neutral versus weight-loss approach for health promotion in women with high BMI: a randomized-controlled trial. *Appetite* 2016;105:364–74

94. Nackers LM, Middleton KR, Dubyak PJ, Daniels MJ, Anton SD, Perri MG. Effects of prescribing 1,000 versus 1,500 kilocalories per day in the behavioral treatment of obesity: a randomized trial. *Obesity* 2013;21:2481–7

95. Nilsen V, Bakke PS, Gallefoss F. Effects of lifestyle intervention in persons at risk for type 2 diabetes mellitus – results from a randomised, controlled trial. *BMC Public Health* 2011;11:893

96. O'Neil PM, Miller-Kovach K, Tuerk PW, et al. Randomized controlled trial of a nationally available weight control program tailored for adults with type 2 diabetes. *Obesity* 2016;24:2269–77

97. Djuric Z, Mirasolo J, Kimbrough L, Brown DR, Heilbrun LK, Canar L, et al. A pilot trial of spirituality counseling for weight loss maintenance in African American breast cancer survivors. *J Natl Med Assoc* 2009;101:552–64

98. Reichard A, Saunders MD, Saunders RR, Donnelly JE, Lauer E, Sullivan DK, Ptomey L. A comparison of two weight management programs for adults with mobility impairments. *Disabil Health J* 2015;8:61–9

For peer review only - http://bmjopen.bmj.com/site/about/guidelines.xhtml

99. Liu P, Ross JS, Ioannidis JP, Dhruva SS, Vasiliou V, Wallach JD. Prevalence and significance of race and ethnicity subgroup analyses in Cochrane intervention reviews. *Clinical Trials* 2020;17(2):231-234

100. Sutcliffe K, Melendez-Torres GJ, Burchet, HED, Richardso, M, Rees R, Thomas J. The importance of service-users' perspectives: A systematic review of qualitative evidence reveals overlooked critical features of weight management programmes. *Health Expect* 2018;21: 563– 573

101. De Las Nueces, D, Hacker K, DiGirolamo, A, Hicks LS. A Systematic Review of Community-Based Participatory Research to Enhance Clinical Trials in Racial and Ethnic Minority Groups. *Health Serv Res* 2012;47:1363-1386

102. Ní Shé, É, Morton, S, Lambert, V, et al. Clarifying the mechanisms and resources that enable the reciprocal involvement of seldom heard groups in health and social care research: A collaborative rapid realist review process. *Health Expect* 2019; 22: 298– 306

103. Arnegard ME, Whitten LA, Hunter C, and Clayton JA. Sex as a Biological Variable: A 5-Year Progress Report and Call to Action. *J Women's Health*. 2020.858-864.

104. Wendler D, Kington R, Madans J, Van Wye G, Christ-Schmidt H, et al. Are racial and ethnic minorities less willing to participate in health research? *PLoS Med* 2006; 3(2): e19.

105. Trial Forge [Internet]. Aberdeen, Scotland: Trial Forge; 2020 [cited February 2021]. The INCLUDE ethnicity framework. Available from: https://www.trialforge.org/trial-forge-centre/include/

106. Travers J, Marsh S, Williams M, et al. External validity of randomised controlled trials in asthma: to whom do the results of the trials apply? *Thorax* 2007;62:219-223

For peer review only - http://bmjopen.bmj.com/site/about/guidelines.xhtml

107. Saunders CD, Byrne B, Guthrie RS, et al. External validity of randomized controlled trials of glycaemic control and vascular disease: how representative are participants? *Diabet Med* 2013;30:300– 308

108. Hoppe C, Kerr D. Minority underrepresentation in cardiovascular outcome trials for type 2 diabetes. *Lancet Diabetes Endocrinol* 2017;5(1):13

109. Trivedi RB, Humphreys K. Participant exclusion criteria in treatment research on neurological disorders: Are unrepresentative study samples problematic? *Contemp Clin Trials* 2015;44:20–25

110. Schmid AF, Groenwold RHH, van Deldena JJM, et al. Justification of exclusion criteria was underreported in a review of cardiovascular trials. *J Clin Epidemiol* 2014;67:635-644

111. He J, Morales DR, Guthrie B. Exclusion rates in randomized controlled trials of treatments for physical conditions: a systematic review. *Trials* 2020;21:228

112. Hayba, N, Partridge SR, Nour MM, Grech A, Allman Farinelli M. Effectiveness of lifestyle interventions for preventing harmful weight gain among young adults from lower socioeconomic status and ethnically diverse backgrounds: a systematic review. *Obes Rev* 2018;19:333– 346

113. Olstad DL, Ancilotto R, Teychenne M, et al. Can targeted policies reduce obesity and improve obesity-related behaviours in socioeconomically disadvantaged populations? A systematic review. *Obes Rev* 2017;18:791– 807

114. Bambra CL, Hillier FC, Cairns J-M, Kasim A, Moore HJ, Summerbell CD. How effective are interventions at reducing socioeconomic inequalities in obesity among children and adults? Two systematic reviews. *Public Health Res* 2015;3(1).

For peer review only - http://bmjopen.bmj.com/site/about/guidelines.xhtml

115. Witham MD, Anderson E, Carroll C, et al. Developing a roadmap to improve trial delivery for under-served groups: results from a UK multi-stakeholder process. *Trials* 2020;21:694

116. Moher D, Hopewell S, Schulz K F, Montori V, GÃ¸tzsche P C, Devereaux P J et al. CONSORT 2010 Explanation and Elaboration: updated guidelines for reporting parallel group randomised trials *BMJ* 2010; 340 :c869

For peer review only

Acknowledgements

We thank the members of the REBALANCE Project and Advisory Groups for their contributions to the REBALANCE project. We thank Shaun Treweek and Heidi Gardner, Health Services Research Unit, University of Aberdeen, for helpful discussions on trial generalisability and inclusion of under-served groups.

Competing interests

None.

Author Contributorship

CR, MA-M, MC, MI, AA contributed to conception and design, acquisition of data, or analysis and interpretation of data, and revised data critically for important intellectual content of this manuscript. All authors critically reviewed the manuscript and approved the final version submitted for publication. CR takes responsibility for the integrity of the data and the accuracy of the data analysis**.**

For peer review only - http://bmjopen.bmj.com/site/about/guidelines.xhtml

Funding

The REBALANCE study was funded by the National Institute for Health Research (NIHR) Health Technology Assessment Programme (Project number: 15/09/04). The views expressed are those of the author(s) and not necessarily those of the NHS, the NIHR or the Department of Health. The Health Services Research Unit is funded by the Chief Scientist Office of the Scottish Government Health and Social Care Directorates.

Transparency statement

CR affirms that the manuscript is an honest, accurate, and transparent account of the study being reported; that no important aspects of the study have been omitted; and that any discrepancies from the study as planned have been explained

Data sharing

Study data are all available from the NIHR journals library ((REBALANCE) REview of Behaviour And Lifestyle interventions for severe obesity: AN evidenCE synthesis (nihr.ac.uk) or can be obtained from the lead author.

For peer review only

For peer review only - http://bmjopen.bmj.com/site/about/guidelines.xhtml

Appendix 1 Literature search strategies

Literature searching was conducted in June 2016 and updated in April/May 2017. The following databases were searched without language restriction: MEDLINE, MEDLINE Epub Ahead of Print & MEDLINE In-Process, PsychINFO, Cumulative Index to Nursing and Allied Health Literature (CINAHL), Science Citation Index (SCI), Cochrane Central Register of Controlled Trials (CENTRAL), and ClincalTrials.gov. Ovid MEDLINE and EMBASE autoalerts have been in place since 2002 and results are screened regularly for long-term RCTs on obesity management in adults (≥ 1 year of follow-up). Copies of relevant reports are retained and the reviewers hand-searched these reports eligible studies. Therefore, search strategies for MEDLINE and EMBASE excluded the results of the autoalert search (using the Boolean operator NOT). A supplementary search of MEDLINE was undertaken to identify systematic reviews of severe or morbid obesity, and reference lists were scrutinised for additional studies.

RCTs for Review 1 (weight loss or weight maintenance programmes for adults with obesity and Review 5 (weight loss or weight maintenance programmes for adults with obesity compared with bariatric surgery)
MEDLINE and EMBASE
Ovid multifile search: http://shibboleth.ovid.com/
Database: Embase <1988 to 2017 Week 17>, Ovid MEDLINE(R) Epub Ahead of Print, In-Process & Other Non-Indexed Citations, Ovid MEDLINE(R) Daily and Ovid MEDLINE(R) <1946 to Present> 25th April 2017
Date of Search: 25th April 2017
1 Obesity, Morbid/dh, dt, su, th use ppez
2 Morbid Obesity/dt, su, th use emed
3 Obesity, Morbid/ use ppez
4 Morbid Obesity/ use emed
5 (obes$ adj3 (morbid$ or severe$)).tw.
6 (morbid obesity or severe obesity).kw.
7 (bmi adj ("27$" or "28$" or "29$")).tw.
8 (bmi adj ("30$" or "31$" or "32$" or "33$" or "34$" or "35$" or "36$" or "37$" or "38$" or "39$")).tw.
9 (bmi adj ("40$" or "41$" or "42$" or "43$" or "44$" or "45$" or "46$" or "47$" or "48$" or "49$")).tw.
10 (bmi adj ("50$" or "51$" or "52$" or "53$" or "54$" or "55$")).tw.
11 (body mass index adj ("35$" or "36$" or "37$" or "38$" or "39$")).tw.
12 (body mass index adj ("40$" or "41$" or "42$" or "43$" or "44$" or "45$" or "46$" or "47$" or "48$" or "49$")).tw.
13 (body mass index adj ("50$" or "51$" or "52$" or "53$" or "54$" or "55$")).tw.
14 or/3-12
15 exp bariatric surgery/
16 bariatric.tw,kw.

For peer review only

17 (gastric adj3 (bypass or bypass or band$ or balloon$)).tw.
18 (gastroplast$ or gastrect$).tw.
19 (jejunoileal adj3 (bypass or by pass)).tw.
20 lipectom$.tw
21 diet therapy/ use ppez or caloric restriction/ use ppez or diet, carbohydrate-restricted/ use ppez or diet, fat-restricted/ use ppez or diet, reducing/ use ppez
22 diet/ use emed or low calory diet/ use emed or low carbohydrate diet/ use emed
23 (diet adj3 (restrict$ or reduc$ or modif$ or calorie$)).tw.
24 (calori$ adj3 (reduc$ or restrict$ or limit$)).tw.
25 exp anti-obesity agents/ use ppez
26 tetrahydrolipstatin/ use emed
27 sibutramine/ use emed
28 rimonabant/ use emed
29 (appetite adj3 (reduc$ or depress$)).tw.
30 (orlistat or xenical).tw,rn.
31 (sibutramin$ or arcalion or reductil or medaria or meridia).tw,rn.
32 (rimonabant or acomplia or zimulti).tw,rn.
33 exp exercise/
34 exp sports/
35 (exercise or aerobic or physical activ$).tw.
36 weight loss/ use ppez
37 weight reduction/ use emed
38 (weight adj3 (reduc$ or loss or lose)).tw.
39 Weight Reduction Programs/ use ppez
40 exp Body Weight Management/ use emed
41 Behavior Therapy/
42 Health Education/
43 Counseling/ use ppez
44 Nutritional counseling/ use emed
45 Life Style/ use ppez
46 Lifestyle modification/ use emed
47 (modif$ adj3 (behavio?r or diet or eat? or eating)).tw.
48 ((diet or weight) adj3 (educat$ or counsel$)).tw.

For peer review only

49 or/15-46
50 1 or 2 or (14 and 49)
51 randomized controlled trial.pt.
52 controlled clinical trial.pt
53 randomi?ed.ab.
54 placebo.ab.
55 drug therapy.fs.
56 randomly.ab.
57 trial.ab.
58 groups.ab
59 nonhuman/ not human/
60 exp clinical trial/ use emed
61 randomization/ use emed
62 51 or 52 or 53 or 54 or 55 or 56 or 57 or 58 or 60 or 61
63 exp animals/ use ppez not humans/ use ppez
64 nonhuman/ use emed or animal/ use emed
65 64 not exp human/ use emed
66 62 not (63 or 65)
67 50 and 66
68 67 not (abstract or letter or note or editorial or comment).pt.
69 limit 68 to yr="1990 -Current"
70 obesity/
71 (obesity adj2 (morbid or diabet$)).tw.
72 obesity, morbid/ use ppez
73 morbid obesity/ use emed
74 obes$.tw.
75 weight loss/ use ppez
76 weight reduction/ use emed
77 (weight adj1 (los$ or reduc$ or maint$ or control)).tw.
78 (diet adj5 weight).tw.
79 overweight.tw.
80 or/70-79
81 80 and 66

For peer review only

82 69 not 81
PsycINFO
Ovid: http://shibboleth.ovid.com/
Database: PsycINFO <1987 to April Week 3 2017>
Date of Search: 25th April 2017
1 (obes$ adj3 (morbid$ or severe$)).tw.
2 (bmi adj ("27$" or "28$" or "29$")).tw.
3 (bmi adj ("30$" or "31$" or "32$" or "33$" or "34$" or "35$" or "36$" or "37$" or "38$" or "39$")).tw.
4 (bmi adj ("40$" or "41$" or "42$" or "43$" or "44$" or "45$" or "46$" or "47$" or "48$" or "49$")).tw.
5 (bmi adj ("50$" or "51$" or "52$" or "53$" or "54$" or "55$")).tw. (
6 (body mass index adj ("27$" or "28$" or "29$")).tw.
7 (body mass index adj ("30$" or "31$" or "32$" or "33$" or "34$" or "35$" or "36$" or "37$" or "38$" or "39$")).tw.
8 (body mass index adj ("40$" or "41$" or "42$" or "43$" or "44$" or "45$" or "46$" or "47$" or "48$" or "49$")).tw.
9 (body mass index adj ("50$" or "51$" or "52$" or "53$" or "54$" or "55$")).tw. (7)
10 or/1-9
11 clinical trials/
12 randomi?ed.tw,md.
13 randomly.tw,md.
14 trial?.tw,md
15 or/11-14
16 10 and 15
CINAHL
http://search.ebscohost.com
!990- 25th April 2017

For peer review only

Date of Search: 25th April 2017

S1 (MH "Obesity, Morbid") OR TX (obes|* N3 (morbid or sever*))
S2 TX (bmi N3 (27 or 28 or 29 or 30 or 31 or 32 or 33 or 34 or 35 or 36 or 37 or 38 or 39).) OR TX (bmi N3 (40 or 41 or 42 or 43 or 44 or 45 or 46 or 47 or 48 or 49).) OR TX (bmi N3 (50 or 51 or 52 or 53 or 54 or 55).)
S3 TX (body mass index N3 (35 or 36 or 37 or 38 or 39).) OR TX (body mass index N3 (40 or 41 or 42 or 43 or 44 or 45 or 46 or 47 or 48 or 49).) OR TX (body mass index N3 (50 or 51 or 52 or 53 or 54 or 55).)
S4 S1 OR S2 OR S3
S5 (MH "Bariatric Surgery+")
S6 TX bariatric OR TX gastroplasty
S7 TX bariatric OR TX gastroplast* AND TX gastrect*
S8 TX (gastric N3 (by pass or by pass or band* or balloon*)) OR TX (jejunoileal N3 (bypass or by pass) OR TX lipectom*
S9 (MH "Diet Therapy") OR (MH "Diet, Reducing") OR (MH "Restricted Diet") OR (MH "Diet, Fat-Restricted") OR (MH "Diet, Low Carbohydrate")
S10 TX (diet N3 (restrict* or reduc* or modif* or calorie*))) OR TX (calori* N3 (reduc* or restrict* or limit*)).)
S11 (MH "Antiobesity Agents+")
S12 TX (orlistat or xenical) OR TX (sibutramin$ or arcalion or reductil or medaria or meridia) OR TX (rimonabant or acomplia or zimulti)
S13 (MH "Exercise+")
S14 TX exercise or aerobic or physical activ*).
S15 (MH "Physical Fitness")
S16 (MH "Weight Loss")
S17 TX weight N3 (reduc* or loss or lose)).
S18 (MH "Weight Reduction Programs")
S19 (MH "Counseling")
S20 (MH "Life Style") OR (MH "Life Style Changes")
S21 TX (modif* N3 (behavior or behaviour or diet or eat* or eating) OR TX ((diet or weight) N3 (educat* or counsel*)).)

For peer review only

S22 S5 OR S6 OR S7 OR S8 OR S9 OR S10 OR S11 OR S12 OR S13 OR S14 OR S15 OR S16 OR S17 OR S18 OR S19 OR S21
S23 S4 AND S22 Limiters - Published Date: 19900101-20161231
S24 S4 AND S22 Limiters - Exclude MEDLINE records

Science Citation Index

www.webofknowledge.com
1988 - 25th April 2017

Date of Search: 25th April 2017

1 (TS=(morbid NEAR/3 obes$)) AND DOCUMENT TYPES: (Article)
2 (TS=(severe$ NEAR/3 obes$)) AND DOCUMENT TYPES: (Article)
3 (TS=(bmi NEAR/3 (27 or 28 or 29 or 30 or 31 or 32 or 33 or 34 or 35 or 36 or 37 or 38 or 39))) AND DOCUMENT TYPES: (Article)
4 ((TS=(bmi NEAR/3 (40 or 41 or 42 or 43 or 44 or 45 or 46 or 47 or 48 or 49)))) AND DOCUMENT TYPES: (Article)
5 ((TS=(bmi NEAR/3 (50 or 51 or 52 or 53 or 54 or 55)))) AND DOCUMENT TYPES: (Article)
6 #5 OR #4 OR #3 OR #2 OR #1
7 (TS=(randomized or randomised or randomly)) AND DOCUMENT TYPES: (Article)
8 (TS=clinical trial*) AND DOCUMENT TYPES: (Article)
9 #8 OR #7
10 #9 AND #6
11 (TS=(bariatric or gastroplast* or gastrect*)) AND DOCUMENT TYPES: (Article)
12 (TS=((gastric or jejunoileal) NEAR/3 bypass)) AND DOCUMENT TYPES: (Article)
13 ((TS=((gastric or jejunoileal) NEAR/3 "by pass"))) AND DOCUMENT TYPES: (Article)
14 (TS=(gastric NEAR/3 (band* or balloon*))) AND DOCUMENT TYPES: (Article)

For peer review only

15 (TS=(diet NEAR/3 (restrict* or reduc* or modif* or calorie*)).) AND DOCUMENT TYPES: (Article)
16 (TS=(calori* NEAR/3 (reduc* or restrict* or limit*)).) AND DOCUMENT TYPES: (Article)
17 (TS=(appetite NEAR/3 (reduc* or depress*)).) AND DOCUMENT TYPES: (Article)
18 (TS=(orlistat or xenical).) AND DOCUMENT TYPES: (Article)
19 (TS=(sibutramin or arcalion or reductil or medaria or meridia).) AND DOCUMENT TYPES: (Article)
20 (TS=(rimonabant or acomplia or zimulti).) AND DOCUMENT TYPES: (Article)
21 (TS=(exercise or aerobic or physical active*).) AND DOCUMENT TYPES: (Article)
22 (TS=(weight NEAR/3 (reduc* or loss or lose))) AND DOCUMENT TYPES: (Article)
23 (TS=(modif* NEAR/3 (behavior or behaviour or diet or eat? or eating)).) AND DOCUMENT TYPES: (Article)
24 (TS=(diet NEAR/3 (educat* or counsel*)).) AND DOCUMENT TYPES: (Article)
25 (TS=(weight NEAR/3 (educat* or counsel*))) AND DOCUMENT TYPES: (Article)
26 #25 OR #24 OR #23 OR #22 OR #21 OR #20 OR #19 OR #18 OR #17 OR #16 OR #15 OR #14 OR #13 OR #12 OR #11
#27 #10 AND #26

Cochrane Central Register of Controlled Trials
www.thecochranelibrary.com
Issue 4 April 2017
Date of Search: 25th April 2017
#1 MeSH descriptor: [Obesity, Morbid] explode all trees and with qualifier(s): [Diet therapy - DH, Surgery - SU, Therapy - TH]

For peer review only

#2 MeSH descriptor: [Obesity, Morbid] explode all trees
#3 obes* near/3 (morbid* or severe*):ti,ab,kw (Word variations have been searched)
#4 bmi near/1 (27* or 28* or 29* or 30* or 31* or 32* or 33* or 34* or 35* or 36* or 37* or 38* or 39*):ti,ab,kw or bmi near/1 (40* or 41* or 42* or 43* or 44* or 45* or 46* or 47* or 48* or 49*):ti,ab,kw or bmi near/1 (50* or 51* or 52* or 53* or 54* or 55*):ti,ab,kw (Word variations have been searched)
#5 body mass index near/1 (27* or 28* or 29* or 30* or 31* or 32* or 33* or 34* or 35* or 36* or 37* or 38* or 39*):ti,ab,kw or body mass index near/1 (40* or 41* or 42* or 43* or 44* or 45* or 46* or 47* or 48* or 49*):ti,ab,kw or body mass index near/1 (50* or 51* or 52* or 53* or 54* or 55*):ti,ab,kw (Word variations have been searched)
#6 #2 or #3 or #4 or #5
#7 MeSH descriptor: [Bariatric Surgery] explode all trees
#8 bariatric:ti,ab,kw (Word variations have been searched)
#9 gastric near/3 (bypass or by pass or band* or balloon*):ti,ab,kw or gastroplast* or gastrect*:ti,ab,kw or lipectom*:ti,ab,kw or jejunoileal near/3 (bypass or by pass):ti,ab,kw (Word variations have been searched)
#10 MeSH descriptor: [Diet Therapy] explode all trees
#11 MeSH descriptor: [Diet, Reducing] explode all trees
#12 MeSH descriptor: [Diet, Fat-Restricted] explode all trees
#13 MeSH descriptor: [Diet, Carbohydrate-Restricted] explode all trees
#14 MeSH descriptor: [Caloric Restriction] explode all trees
#15 diet near/3 (restrict* or reduc* or modif* or calorie*):ti,ab,kw or calori* near/3 (reduc* or restrict* or limit*):ti,ab,kw (Word variations have been searched)
#16 MeSH descriptor: [Anti-Obesity Agents] explode all trees
#17 appetite near/3 (reduc* or depress*):ti,ab,kw or orlistat or xenical:ti,ab,kw or sibutramin or arcalion or reductil or medaria or meridia:ti,ab,kw or rimonabant or acomplia or zimulti:ti,ab,kw (Word variations have been searched)
#18 MeSH descriptor: [Exercise] explode all trees
#19 MeSH descriptor: [Sports] explode all trees
#20 exercise or aerobic* or physical activ*:ti,ab,kw (Word variations have been searched)

For peer review only

#21 MeSH descriptor: [Weight Loss] explode all trees
#22 MeSH descriptor: [Weight Reduction Programs] explode all trees
#23 weight near/3 (reduc* or loss or lose) .:ti,ab,kw (Word variations have been searched)
#24 weight near/3 (reduc* or loss or lose) .:ti,ab,kw (Word variations have been searched)
#25 MeSH descriptor: [Behavior Therapy] explode all trees
#26 MeSH descriptor: [Health Education] explode all trees
#27 MeSH descriptor: [Counseling] explode all trees
#28 MeSH descriptor: [Life Style] explode all trees
#29 diet near/3 (educat* or counsel*):ti,ab,kw or modif* near/3 (behaviour or behavior or diet or eat or eating):ti,ab,kw or weight near/3 (educat* or counsel*):ti,ab,kw (Word variations have been searched)
#30 #7 or #8 or #9 or #10 or #11 or #12 or #13 or #14 or #15 or #16 or #17 or #18 or #19 or #20 or #21 or #22 or #23 or #24 or #25 or #26 or #27 or #28 or #29
#31 #6 and #30
#32 #1 or #31 Publication Year from 1990 to 2016, in Trials
#33 conference abstract (Word variations have been searched)
#34 #32 not #33

For peer review only

Appendix 2 PRISMA flow diagram

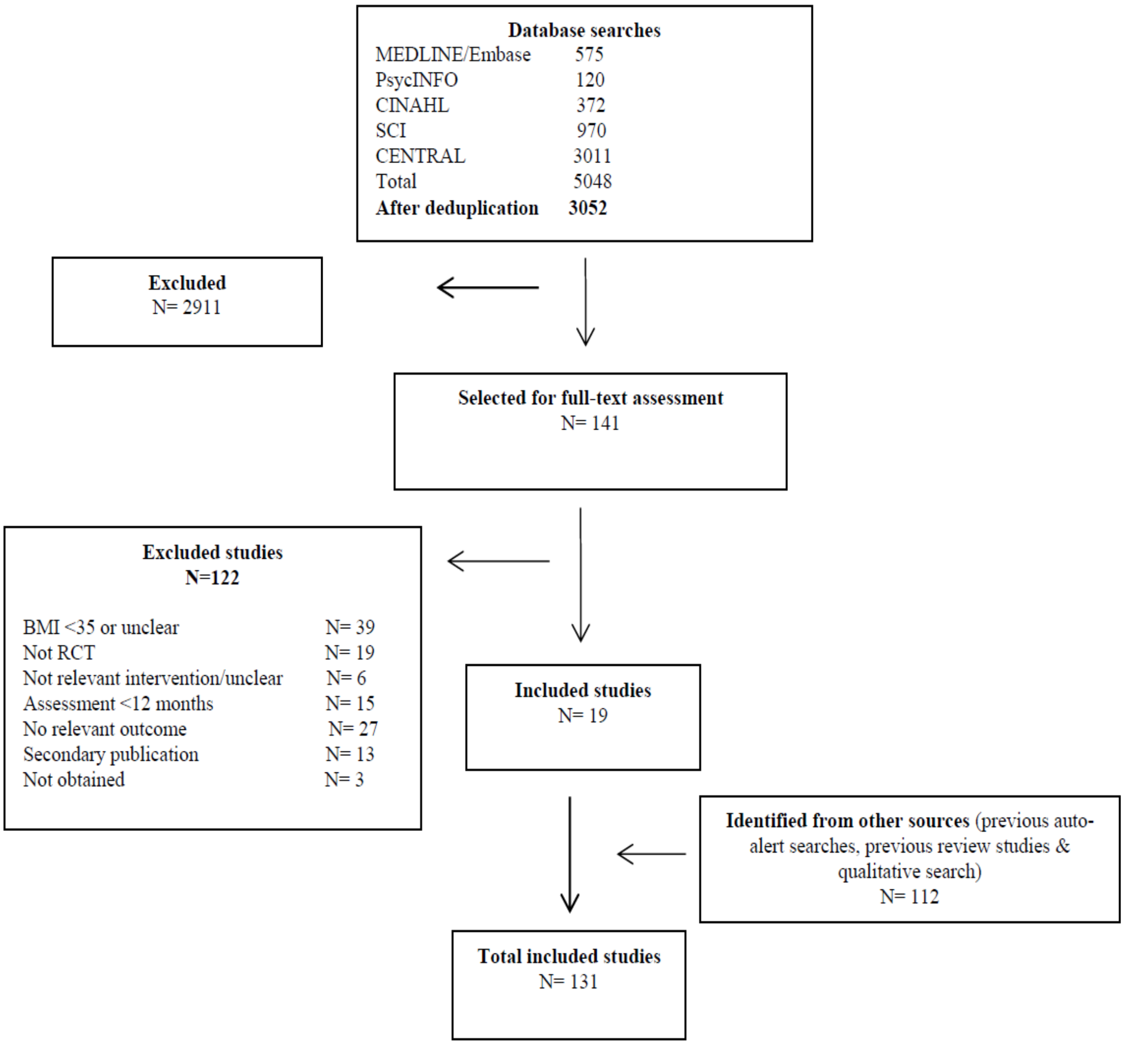

Appendix 3 List of included studies

Agras WS, Berkowitz RI, Arnow BA, Telch CF, Marnell M, Henderson J, et al. Maintenance following a very-low-calorie diet. *Journal of Consulting & Clinical Psychology* 1996;**64:**610-3.

Annesi JJ. Mediation of the relationship of behavioural treatment type and changes in psychological predictors of healthy eating by body satisfaction changes in women with obesity. *Obesity Research & Clinical Practice* 2017;**11:**97-107.

Appel LJ, Clark JM, Yeh HC, Wang NY, Coughlin JW, Daumit G, et al. Comparative effectiveness of weight-loss interventions in clinical practice. *New England Journal of Medicine* 2011;**365:**1959-68.

Azar KM, Xiao L, Ma J. Baseline obesity status modifies effectiveness of adapted diabetes prevention program lifestyle interventions for weight management in primary care. *BioMed Research International* 2013;191209.

Bacon L, Keim NL, Van Loan MD, Derricote M, Gale B, Kazaks A, et al. Evaluating a 'non-diet' wellness intervention for improvement of metabolic fitness, psychological well-being and eating and activity behaviors. *International Journal of Obesity & Related Metabolic Disorders* 2002;**26:**854-65.

Bakris G, Calhoun D, Egan B, Hellmann C, Dolker M, Kingma I. Orlistat improves blood pressure control in obese subjects with treated but inadequately controlled hypertension. *Journal of Hypertension* 2002;**20:**2257-67.

Bartels SJ, Pratt SI, Aschbrenner KA, Barre LK, Jue K, Wolfe RS, et al. Clinically Significant Improved Fitness and Weight Loss Among Overweight Persons With Serious Mental Illness. *Psychiatric Services* 2013;**64:**729-36.

Bartels SJ, Pratt SI, Aschbrenner KA, Barre LK, Naslund JA, Wolfe R, et al. Pragmatic Replication Trial of Health Promotion Coaching for Obesity in Serious Mental Illness and Maintenance of Outcomes. *American Journal of Psychiatry* 2015;**172:**344-52.

Bazzano LA, Hu T, Reynolds K, Yao L, Bunol C, Liu Y, et al. Effects of low-carbohydrate and low-fat diets: a randomized trial. *Annals of Internal Medicine* 2014;**161:**309-18.

Bennett GG, Warner ET, Glasgow RE, Askew S, Goldman J, Ritzwoller DP, et al. Obesity treatment for socioeconomically disadvantaged patients in primary care practice. *Archives of Internal Medicine* 2012;**172:**565-74.

Berry DC, Schwartz TA, McMurray RG, Skelly AH, Neal M, Hall EG, et al. The family partners for health study: a cluster randomized controlled trial for child and parent weight management. *Nutrition & Diabetes* 2014;**4:**e101.

For peer review only

Berteus Forslund H, Klingstrom S, Hagberg H, Londahl M, Torgerson JS, Lindroos AK. Should snacks be recommended in obesity treatment? A 1-year randomized clinical trial. *European Journal of Clinical Nutrition* 2008;**62:**1308-17.

Beutel ME, Dippel A, Szczepanski M, Thiede R, Wiltink J. Mid-term effectiveness of behavioral and psychodynamic inpatient treatments of severe obesity based on a randomized study. *Psychotherapy and Psychosomatics* 2006;**75:**337-45.

Bliddal H, Leeds AR, Stigsgaard L, Astrup A, Christensen R. Weight loss as treatment for knee osteoarthritis symptoms in obese patients: 1-Year results from a randomised controlled trial. *Annals of the Rheumatic Diseases* 2011;**70:**1798-803.

Brehm BJ, Lattin BL, Summer SS, Boback JA, Gilchrist GM, Jandacek RJ, et al. One-year comparison of a high-monounsaturated fat diet with a high-carbohydrate diet in type 2 diabetes. *Diabetes Care* 2009;**32:**215-20.

Broom I, Hughes E, Dodson P, Reckless J. The role of orlistat in the treatment of obese patients with mild to moderate hypercholesterolaemia: Consequences for coronary risk. *British Journal of Cardiology* 2002;**9:**460-1+8.

Broom I, Wilding J, Stott P, Myers N. Randomised trial of the effect of orlistat on body weight and cardiovascular disease risk profile in obese patiensts: UK multimorbidity study. *International Journal of Clinical Practice* 2002;**56:**494-9.

Burguera B, Jesus Tur J, Escudero AJ, Alos M, Pagan A, Cortes B, et al. An Intensive Lifestyle Intervention Is an Effective Treatment of Morbid Obesity: The TRAMOMTANA Study-A Two-Year Randomized Controlled Clinical Trial. *International journal of endocrinology* 2015.

Cheskin LJ, Mitchell AM, Jhaveri AD, Mitola AH, Davis LM, Lewis RA, et al. Efficacy of meal replacements versus a standard food-based diet for weight loss in type 2 diabetes: a controlled clinical trial. *Diabetes Educator* 2008;**34:**118-27.

Christensen P, Frederiksen R, Bliddal H, Riecke BF, Bartels EM, Henriksen M, et al. Comparison of three weight maintenance programs on cardiovascular risk, bone and vitamins in sedentary older adults. *Obesity* 2013;**21:**1982-90.

Courcoulas AP, Goodpaster BH, Eagleton JK, Belle SH, Kalarchian MA, Lang W, et al. Surgical vs medical treatments for type 2 diabetes mellitus: a randomized clinical trial. *JAMA Surgery* 2014;**149:**707-15.

Cummings DE, Arterburn DE, Westbrook EO, Kuzma JN, Stewart SD, Chan CP, et al. Gastric bypass surgery vs intensive lifestyle and medical intervention for type 2 diabetes: the CROSSROADS randomised controlled trial. *Diabetologia* 2016;**59:**945-53.

For peer review only

Dalle Grave R, Calugi S, Gavasso I, El Ghoch M, Marchesini G. A randomized trial of energy-restricted high-protein versus high-carbohydrate, low-fat diet in morbid obesity. *Obesity* 2013;**21:**1774-81.

Damschroder LJ, Lutes LD, Kirsh S, Kim HM, Gillon L, Holleman RG, et al. Small-changes obesity treatment among veterans: 12-month outcomes. *American Journal of Preventive Medicine* 2014;**47:**541-53.

Daubenmier J, Moran PJ, Kristeller J, Acree M, Bacchetti P, Kemeny ME, et al. Effects of a mndfulness-based weight loss intervention in adults with obesity: A randomized clinical trial. *Obesity* 2016;**24:**794-804.

Daumit GL, Dickerson FB, Wang NY, Dalcin A, Jerome GJ, Anderson CAM, et al. A behavioral weight-loss intervention in persons with serious mental illness. *New England Journal of Medicine* 2013;**368:**1594-602.

Davidson MH, Hauptman J, DiGirolamo M, Foreyt JP, Halsted CH, Heber D, et al. Weight control and risk factor reduction in obese subjects treated for 2 years with orlistat: A randomized controlled trial. *Journal of the American Medical Association* 1999;**281:**235-42.

Davis NJ, Tomuta N, Schechter C, Isasi CR, Segal-Isaacson CJ, Stein D, et al. Comparative study of the effects of a 1-year dietary intervention of a low-carbohydrate diet versus a low-fat diet on weight and glycemic control in type 2 diabetes. *Diabetes Care* 2009;**32:**1147-52.

Delbridge EA, Prendergast LA, Pritchard JE, Proietto J. One-year weight maintenance after significant weight loss in healthy overweight and obese subjects: Does diet composition matter? *American Journal of Clinical Nutrition* 2009;**90:**1203-14.

Dennison KF, Galante D, Dennison D, Golaszewski T. A one year post-program assessment of a computer-assisted instruction weight management program for industrial employees: lessons learned. *Journal of Health Education* 1996;**27:**38-43.

Dixon JB, O'Brien PE, Playfair J, Chapman L, Schachter LM, Skinner S, et al. Adjustable gastric banding and conventional therapy for type 2 diabetes: a randomized controlled trial. *JAMA* 2008;**299:**316-23.

Dixon JB, Schachter LM, O'Brien PE, Jones K, Grima M, Lambert G, et al. Surgical vs conventional therapy for weight loss treatment of obstructive sleep apnea: A randomized controlled trial. *JAMA* 2012;**308:**1142-9.

Djuric Z, Mirasolo J, Kimbrough L, Brown DR, Heilbrun LK, Canar L, et al. A pilot trial of spirituality counseling for weight loss maintenance in African American breast cancer survivors. *Journal of the National Medical Association* 2009;**101:**552-64.

For peer review only

Dutton GR, Nackers LM, Dubyak PJ, Rushing NC, Huynh TVT, Tan F, et al. A randomized trial comparing weight loss treatment delivered in large versus small groups. *International Journal of Behavioral Nutrition and Physical Activity* 2014;**11 (1)**

Eaton CB, Hartman SJ, Perzanowski E, Pan GH, Roberts MB, Risica PM, et al. A Randomized Clinical Trial of a Tailored Lifestyle Intervention for Obese, Sedentary, Primary Care Patients. *Annals of Family Medicine* 2016;**14:**311-9.

Ebbeling CB, Leidig MM, Feldman HA, Lovesky MM, Ludwig DS. Effects of a low-glycemic load vs low-fat diet in obese young adults: A randomized trial. *Journal of the American Medical Association* 2007;**297:**2092-102.

Esposito K, Giugliano F, Di Palo C, Giugliano G, Marfella R, D'Andrea F, et al. Effect of lifestyle changes on erectile dysfunction in obese men: A randomized controlled trial. *Journal of the American Medical Association* 2004;**291:**2978-84.

Finer N, James WPT, Kopelman PG, Lean MEJ, Williams G. One-year treatment of obesity: A randomized, double-blind, placebo-controlled, multicentre study of orlistat, a gastrointestinal lipase inhibitor. *International Journal of Obesity* 2000;**24:**306-13.

Fitzgibbon ML, Stolley MR, Schiffer L, Sharp LK, Singh V, Dyer A. Obesity reduction black intervention trial (ORBIT): 18-month results. *Obesity* 2010;**18:**2317-25.

Flechtner-Mors M, Boehm BO, Wittmann R, Thoma U, Ditschuneit HH. Enhanced weight loss with protein-enriched meal replacements in subjects with the metabolic syndrome. *Diabetes/Metabolism Research and Reviews* 2010;**26:**393-405.

Foster GD, Wyatt HR, Hill JO, Makris AP, Rosenbaum DL, Brill C, et al. Weight and metabolic outcomes after 2 years on a low-carbohydrate versus low-fat diet: A randomized trial. *Annals of Internal Medicine* 2010;**153:**147-57.

Goodpaster BH, Delany JP, Otto AD, Kuller L, Vockley J, South-Paul JE, et al. Effects of diet and physical activity interventions on weight loss and cardiometabolic risk factors in severely obese adults: a randomized trial. *JAMA* 2010;**304:**1795-802.

Gorin AA, Raynor HA, Fava J, Maguire K, Robichaud E, Trautvetter J, et al. Randomized controlled trial of a comprehensive home environment-focused weight-loss program for adults. *Health Psychology* 2013;**32:**128-37.

Green CA, Yarborough BJH, Leo MC, Stumbo SP, Perrin NA, Nichols GA, et al. Weight maintenance following the STRIDE lifestyle intervention for individuals taking antipsychotic medications. *Obesity* 2015;**23:**1995-2001.

Hakala P, Karvetti RL, Rönnemaa T. Group vs. individual weight reduction programmes in the treatment of severe obesity--a five year follow-up study. *International journal of obesity and related metabolic disorders* 1993;**17:**97-102.
Hakala P. Weight reduction programmes at a rehabilitation centre and a health centre based on group counselling and individual support: Short- and long-term follow-up study. *International Journal of Obesity* 1994;**18:**483-9.

For peer review only

Hauptman J, Lucas C, Boldrin MN, Collins H, Segal KR. Orlistat in the long-term treatment of obesity in primary care settings. *Archives of Family Medicine* 2000;**9:**160-7.

Hunt K, Wyke S, Gray CM, Anderson AS, Brady A, Bunn C, et al. A gender-sensitised weight loss and healthy living programme for overweight and obese men delivered by Scottish Premier League football clubs (FFIT): A pragmatic randomised controlled trial. *The Lancet* 2014;**383:**1211-21.

Iqbal N, Vetter ML, Moore RH, Chittams JL, Dalton-Bakes CV, Dowd M, et al. Effects of a low-intensity intervention that prescribed a low-carbohydrate vs. a low-fat diet in obese, diabetic participants. *Obesity* 2010;**18:**1733-8.

Jesudason DR, Pedersen E, Clifton PM. Weight-loss diets in people with type 2 diabetes and renal disease: a randomized controlled trial of the effect of different dietary protein amounts. *American Journal of Clinical Nutrition* 2013;**98:**494-501.

Kahleova H, Hill M, Pelikanova T. Vegetarian vs. conventional diabetic diet - A 1-year follow-up. *Cor et Vasa* 2014;**56:**e140-e4.

Kelley DE, Bray GA, Pi-Sunyer FX, Klein S, Hill J, Miles J, et al. Clinical efficacy of orlistat therapy in overweight and obese patients with insulin-treated type 2 diabetes: A 1-year randomized controlled trial. *Diabetes Care* 2002;**25:**1033-41.

Keranen AM, Savolainen MJ, Reponen AH, Kujari ML, Lindeman SM, Bloigu RS, et al. The effect of eating behavior on weight loss and maintenance during a lifestyle intervention. *Preventive Medicine* 2009;**49:**32-8.

Khoo J, Piantadosi C, Duncan R, Worthley SG, Jenkins A, Noakes M, et al. Comparing effects of a low-energy diet and a high-protein low-fat diet on sexual and endothelial function, urinary tract symptoms, and inflammation in obese diabetic men. *Journal of Sexual Medicine* 2011;**8:**2868-75.

Krebs JD, Elley CR, Parry-Strong A, Lunt H, Drury PL, Bell DA, et al. The Diabetes Excess Weight Loss (DEWL) Trial: A randomised controlled trial of high-protein versus high-carbohydrate diets over 2 years in type 2 diabetes. *Diabetologia* 2012;**55:**905-14.

Krempf M, Louvet JP, Allanic H, Miloradovich T, Joubert JM, Attali JR. Weight reduction and long-term maintenance after 18 months treatment with orlistat for obesity. *International Journal of Obesity* 2003;**27:**591-7.

For peer review only

Kumanyika SK, Fassbender JE, Sarwer DB, Phipps E, Allison KC, Localio R, et al. One-year results of the think health study of weight management in primary care practices. *Obesity* 2012;**20:**1249-57.

Kumanyika SK, Shults J, Fassbender J, Whitt MC, Brake V, Kallan MJ, et al. Outpatient weight management in African-Americans: The Healthy Eating and Lifestyle Program (HELP) study. *Preventive Medicine* 2005;**41:**488-502.

Kumanyika SK, Wadden TA, Shults J, Fassbender JE, Brown SD, Bowman MA, et al. Trial of family and friend support for weight loss in African American adults. *Archives of Internal Medicine* 2009;**169:**1795-804.

Lantz H, Peltonen M, Agren L, Torgerson JS. Intermittent versus on-demand use of a very low calorie diet: A randomized 2-year clinical trial. *Journal of Internal Medicine* 2003;**253:**463-71.

Latner JD, Ciao AC, Wendicke AU, Murakami JM, Durso LE. Community-based behavioral weight-loss treatment: Long-term maintenance of weight loss, physiological, and psychological outcomes. *Behaviour Research and Therapy* 2013;**51:**451-9.

Lillis J, Niemeier HM, Thomas JG, Unick J, Ross KM, Leahey TM, et al. A Randomized Trial of an Acceptance-Based Behavioral Intervention for Weight Loss in People with High Internal Disinhibition. *Obesity* 2016;**24:**2509-14.

Linde JA, Simon GE, Ludman EJ, Ichikawa LE, Operskalski BH, Arterburn D, et al. A randomized controlled trial of behavioral weight loss treatment versus combined weight loss/depression treatment among women with comorbid obesity and depression. *Annals of behavioral medicine* 2011;**41:**119-30.

Little P, Stuart B, Hobbs FR, Kelly J, Smith ER, Bradbury KJ, et al. An internet-based intervention with brief nurse support to manage obesity in primary care (POWeR+): a pragmatic, parallel-group, randomised controlled trial. *The Lancet Diabetes and Endocrinology* 2016;**4:**821-8.

Look Ahead Research Group, Wing RR, Bolin P, Brancati FL, Bray GA, Clark JM, et al. Cardiovascular effects of intensive lifestyle intervention in type 2 diabetes.[Erratum appears in N Engl J Med. 2014 May 8;370(19):1866]. *New England Journal of Medicine* 2013;**369:**145-54.

Lowe MR, Butryn ML, Thomas JG, Coletta M. Meal replacements, reduced energy density eating, and weight loss maintenance in primary care patients: a randomized controlled trial. *Obesity* 2014;**22:**94-100.

Ma J, Strub P, Xiao L, Lavori PW, Camargo CA, Jr., Wilson SR, et al. Behavioral weight loss and physical activity intervention in obese adults with asthma. A randomized trial. *Annals of the American Thoracic Society* 2015;**12:**1-11.

Maclaughlin HL, Hall WL, Patel AG, Blacklock RM, Swift PA, Phanish MK, et al. Weight loss, adipokines, and quality of life after sleeve gastrectomy in obese patients with stages 3-4 CKD: A randomized controlled pilot study. *American Journal of Kidney Diseases* 2014;**64:**660-3.

Manzoni GM, Cesa GL, Bacchetta M, Castelnuovo G, Conti S, Gaggioli A, et al. Virtual reality-enhanced cognitive-behavioral therapy for morbid obesity: A randomized controlled study with 1 year follow-up. *Cyberpsychology, Behavior, and Social Networking* 2016;**19:**134-40.

Martin PD, Dutton GR, Rhode PC, Horswell RL, Ryan DH, Brantley PJ. Weight loss maintenance following a primary care intervention for low-income minority women. *Obesity* 2008;**16:**2462-7.

Mayer-Davis EJ, D'Antonio AM, Smith SM, Kirkner G, Martin SL, Parra-Medina D, et al. Pounds off with empowerment (POWER): A clinical trial of weight management strategies for black and white adults with diabetes who live in medically underserved rural communities. *American Journal of Public Health* 2004;**94:**1736-42.

McRobbie H, Hajek P, Peerbux S, Kahan BC, Eldridge S, Trepel D, et al. Tackling obesity in areas of high social deprivation: clinical effectiveness and cost-effectiveness of a task-based weight management group programme - a randomised controlled trial and economic evaluation. *Health Technology Assessment* 2016;**20(79).**.

Melin I, Karlstrom B, Lappalainen R, Berglund L, Mohsen R, Vessby B. A programme of behaviour modification and nutrition counselling in the treatment of obesity: A randomised 2-y clinical trial. *International Journal of Obesity* 2003;**27:**1127-35.

Mensinger JL, Calogero RM, Stranges S, Tylka TL. A weight-neutral versus weight-loss approach for health promotion in women with high BMI: A randomized-controlled trial. *Appetite* 2016;**105:**364-74.

Miles JM, Leiter L, Hollander P, Wadden T, Anderson JW, Doyle M, et al. Effect of orlistat in overweight and obese patients with type 2 diabetes treated with metformin. *Diabetes Care* 2002;**25:**1123-8.

Mingrone G, Greco AV, Giancaterini A, Scarfone A, Castagneto M, Pugeat M. Sex hormone-binding globulin levels and cardiovascular risk factors in morbidly obese subjects before and after weight reduction induced by diet or malabsorptive surgery. *Atherosclerosis* 2002;**161:**455-62.

Mingrone G, Panunzi S, De Gaetano A, Guidone C, Iaconelli A, Leccesi L, et al. Bariatric surgery versus conventional medical therapy for type 2 diabetes. *New England Journal of Medicine* 2012;**366:**1577-85.

For peer review only

Moreno B, Bellido D, Sajoux I, Goday A, Saavedra D, Crujeiras AB, et al. Comparison of a very low-calorie-ketogenic diet with a standard low-calorie diet in the treatment of obesity. *Endocrine* 2014;**47:**793-805.

Nackers LM, Middleton KR, Dubyak PJ, Daniels MJ, Anton SD, Perri MG. Effects of prescribing 1,000 versus 1,500 kilocalories per day in the behavioral treatment of obesity: A randomized trial. *Obesity* 2013;**21:**2481-7.

Nilsen V, Bakke PS, Gallefoss F. Effects of lifestyle intervention in persons at risk for type 2 diabetes mellitus - results from a randomised, controlled trial. *BMC public health* 2011;**11:**893.

O'Neil PM, Miller-Kovach K, Tuerk PW, Becker LE, Wadden TA, Fujioka K, et al. Randomized Controlled Trial of a Nationally Available Weight Control Program Tailored for Adults with Type 2 Diabetes. *Obesity* 2016;**24:**2269-77.

Ostbye T, Stroo M, Brouwer RJ, Peterson BL, Eisenstein EL, Fuemmeler BF, et al. Steps to Health employee weight management randomized Control trial: Short-term follow-up results. *Journal of Occupational and Environmental Medicine* 2015;**57:**188-95.

Pascale RW, Wing RR, Butler BA, Mullen M, Bononi P. Effects of a behavioral weight loss program stressing calorie restriction versus calorie plus fat restriction in obese individuals with NIDDM or a family history of diabetes. *Diabetes Care* 1995;**18:**1241-8.

Pedersen E, Jesudason DR, Clifton PM. High protein weight loss diets in obese subjects with type 2 diabetes mellitus. *Nutrition, Metabolism and Cardiovascular Diseases* 2014;**24:**554-62.

Pekkarinen T, Kaukua J, Mustajoki P. Long-term weight maintenance after a 17-week weight loss intervention with or without a one-year maintenance program: A randomized controlled trial. *Journal of Obesity* 2015;Article 651460

Perri MG, Limacher MC, Durning PE, Janicke DM, Lutes LD, Bobroff LB, et al. Extended-care programs for weight management in rural communities: The Treatment of Obesity in Underserved Rural Settings (TOURS) randomized trial. *Archives of Internal Medicine* 2008;**168:**2347-54.

Perri MG, Limacher MC, Von Castel-Roberts K, Daniels MJ, Durning PE, Janicke DM, et al. Comparative effectiveness of three doses of weight-loss counseling: Two-year findings from the Rural LITE Trial. *Obesity* 2014;**22:**2293-300.

Perri MG, Nezu AM, McKelvey WF, Shermer RL, Renjilian DA, Viegener BJ. Relapse prevention training and problem-solving therapy in the long-term management of obesity. *Journal of Consulting and Clinical Psychology* 2001;**69:**722-6.

For peer review only

Poston WS, Reeves RS, Haddock CK, Stormer S, Balasubramanyam A, Satterwhite O, et al. Weight loss in obese Mexican Americans treated for 1-year with orlistat and lifestyle modification. *International Journal of Obesity & Related Metabolic Disorders* 2003;**27:**1486-93.

Purcell K, Sumithran P, Prendergast LA, Bouniu CJ, Delbridge E, Proietto J. The effect of rate of weight loss on long-term weight management: A randomised controlled trial. *The Lancet Diabetes and Endocrinology* 2014;**2:**954-62.

Rapoport L, Clark M, Wardle J. Evaluation of a modified cognitive-behavioural programme for weight management. *International Journal of Obesity* 2000;**24:**1726-37.

Reichard A, Saunders MD, Saunders RR, Donnelly JE, Lauer E, Sullivan DK, et al. A comparison of two weight management programs for adults with mobility impairments. *Disability and Health Journal* 2015;**8:**61-9.

Reis LO, Favaro WJ, Barreiro GC, Oliveira LC, Chaim EA, Fregonesi A, et al. Erectile dysfunction and hormonal imbalance in morbidly obese male is reversed after gastric bypass surgery: a prospective randomized controlled trial. *International journal of andrology* 2010;**33:**736-44.

Richelsen B, Tonstad S, Rossner S, Toubro S, Niskanen L, Madsbad S, et al. Effect of orlistat on weight regain and cardiovascular risk factors following a very-low-energy diet in abdominally obese patients: A 3-year randomized, placebo-controlled study. *Diabetes Care* 2007;**30:**27-32.

Rock CL, Flatt SW, Pakiz B, Taylor KS, Leone AF, Brelje K, et al. Weight loss, glycemic control, and cardiovascular disease risk factors in response to differential diet composition in a weight loss program in type 2 diabetes: A randomized controlled trial. *Diabetes Care* 2014;**37:**1573-80.

Rossner S, Flaten H. VLCD versus LCD in long-term treatment of obesity. *International Journal of Obesity* 1997;**21:**22-6.

Ryttig KR, Flaten H, Rossner S. Long-term effects of a very low calorie diet (Nutrilett) in obesity treatment. A prospective, randomized, comparison between VLCD and a hypocaloric diet + behavior modification and their combination. *International Journal of Obesity* 1997;**21:**574-9.

Ryttig KR, Rossner S. Weight maintenance after a very low calorie diet (VLCD) weight reduction period and the effects of VLCD supplementation. A prospective, randomized, comparative, controlled long-term trial. *Journal of Internal Medicine* 1995;**238:**299-306.

Schauer PR, Kashyap SR, Wolski K, Brethauer SA, Kirwan JP, Pothier CE, et al. Bariatric surgery versus intensive medical therapy in obese patients with diabetes. *New England Journal of Medicine* 2012;**366:**1567-76.
Shikany JM, Thomas AS, Beasley TM, Lewis CE, Allison DB. Randomized controlled trial of the Medifast 5 & 1 plan for weight loss. *International Journal of Obesity* 2013;**37:**1571-8.

Sjostrom L, Rissanen A, Andersen T, Boldrin M, Golay A, Koppeschaar HPF, et al.

For peer review only

Randomised placebo-controlled trial of orlistat for weight loss and prevention of weight regain in obese patients. *Lancet* 1998;**352:**167-72.

Soenen S, Bonomi AG, Lemmens SGT, Scholte J, Thijssen MAMA, Van Berkum F, et al. Relatively high-protein or 'low-carb' energy-restricted diets for body weight loss and body weight maintenance? *Physiology and Behavior* 2012;**107:**374-80.

Spring B, Duncan JM, Janke EA, Kozak AT, McFadden HG, Demott A, et al. Integrating technology into standard weight loss treatment a randomized controlled trial. *JAMA Internal Medicine* 2013;**173:**105-11.

Stahre L, Hallstrom T. A short-term cognitive group treatment program gives substantial weight reduction up to 18 months from the end of treatment. A randomized controlled trial. *Eating and Weight Disorders* 2005;**10:**51-8.

Stenius-Aarniala B, Poussa T, Kvarnstrom J, Gronlund EL, Ylikahri M, Mustajoki P. Immediate and long term effects of weight reduction in obese people with asthma: Randomised controlled study. *British Medical Journal* 2000;**320:**827-32.

Stern L, Iqbal N, Seshadri P, Chicano KL, Daily DA, McGrory J, et al. The Effects of Low-Carbohydrate versus Conventional Weight Loss Diets in Severely Obese Adults: One-Year Follow-up of a Randomized Trial. *Annals of Internal Medicine* 2004;**140:**778-85+I-27.

Strobl V, Knisel W, Landgraf U, Faller H. A combined planning and telephone aftercare intervention for obese patients: effects on physical activity and body weight after one year. *Journal of rehabilitation medicine* 2013;**45:**198-205.

Swinburn BA, Carey D, Hills AP, Hooper M, Marks S, Proietto J, et al. Effect of orlistat on cardiovascular disease risk in obese adults. *Diabetes Obesity & Metabolism* 2005;**7:**254-62.

Torgerson J, Agren L, Sjostrom L. Effects on body weight of strict or liberal adherence to an initial period of VLCD treatment. A randomised, one-year clinical trial of obese subjects. *International Journal of Obesity* 1999;**23:**190-7.

Torgerson JS, Hauptman J, Boldrin MN, Sjostrom L. XENical in the Prevention of Diabetes in Obese Subjects (XENDOS) Study: A randomized study of orlistat as an adjunct to lifestyle changes for the prevention of type 2 diabetes in obese patients. *Diabetes Care* 2004;**27:**155-61.

For peer review only

Torgerson JS, Lissner L, Lindroos AK, Kruijer H, Sjostrom L. VLCD plus dietary and behavioural support versus support alone in the treatment of severe obesity. A randomised two-year clinical trial. *International Journal of Obesity* 1997;**21:**987-94.

Tsai AG, Wadden TA, Rogers MA, Day SC, Moore RH, Islam BJ. A Primary Care Intervention for Weight Loss: Results of a Randomized Controlled Pilot Study. *Obesity* 2010;**18:**1614-8.

Van Name MA, Camp AW, Magenheimer EA, Li F, Dziura JD, Montosa A, et al. Effective translation of an intensive lifestyle intervention for hispanic women with prediabetes in a community health center setting. *Diabetes Care* 2016;**39:**525-31.

Villareal DT, Chode S, Parimi N, Sinacore DR, Hilton T, Armamento-Villareal R, et al. Weight loss, exercise, or both and physical function in obese older adults. *New England Journal of Medicine* 2011;**364:**1218-29.

Villareal DT, Shah K, Banks MR, Sinacore DR, Klein S. Effect of weight loss and exercise therapy on bone metabolism and mass in obese older adults: A one-year randomized controlled trial. *Journal of Clinical Endocrinology and Metabolism* 2008;**93:**2181-7.

von Gruenigen VE, Courneya KS, Gibbons HE, Kavanagh MB, Waggoner SE, Lerner E. Feasibility and effectiveness of a lifestyle intervention program in obese endometrial cancer patients: A randomized trial. *Gynecologic Oncology* 2008;**109:**19-26.

Wadden TA, Foster GD, Letizia KA. One-year behavioral treatment of obesity: Comparison of moderate and severe caloric restriction and the effects of weight maintenance therapy. *Journal of Consulting and Clinical Psychology* 1994;**62:**165-71.

Wadden TA, Berkowitz RI, Vogt RA, Steen SN, Stunkard AJ, Foster GD. Lifestyle modification in the pharmacologic treatment of obesity: a pilot investigation of a potential primary care approach. *Obesity research* 1997;**5:**218-26.

Wadden TA, Vogt RA, Foster GD, Anderson DA. Exercise and the maintenance of weight loss: 1-year follow-up of a controlled clinical trial. *Journal of Consulting and Clinical Psychology* 1998;**66:**429-33.

Wadden TA, Volger S, Sarwer DB, Vetter ML, Tsai AG, Berkowitz RI, et al. A two-year randomized trial of obesity treatment in primary care practice. *New England Journal of Medicine* 2011;**365:**1969-79.

Weinstock RS, Trief PM, Cibula D, Morin PC, Delahanty LM. Weight Loss Success in Metabolic Syndrome by Telephone Interventions: Results from the SHINE Study. *Journal of General Internal Medicine* 2013;**28:**1620-8.

For peer review only

West DS, DiLillo V, Bursac Z, Gore SA, Greene PG. Motivational interviewing improves weight loss in women with type 2 diabetes. *Diabetes Care* 2007;**30:**1081-7.

West DS, Gorin AA, Subak LL, Foster G, Bragg C, Hecht J, et al. A motivation-focused weight loss maintenance program is an effective alternative to a skill-based approach. *International Journal of Obesity* 2011;**35:**259-69.

Wing RR, Blair E, Marcus M, Epstein LH, Harvey J. Year-long weight loss treatment for obese patients with type II diabetes: Does including an intermittent very-low-calorie diet improve outcome? *American Journal of Medicine* 1994;**97:**354-62.

Wing RR, Marcus MD, Epstein LH, Jawad A. A "family-based" approach to the treatment of obese type II diabetic patients. *Journal of Consulting and Clinical Psychology* 1991;**59:**156-62.

Wing RR, Marcus MD, Salata R, Epstein LH, Miaskiewicz S, Blair EH. Effects of a very-low-calorie diet on long-term glycemic control in obese type 2 diabetic subjects. *Archives of Internal Medicine* 1991;**151:**1334-40.

Wing RR, Venditti E, Jakicic JM, Polley BA, Lang W. Lifestyle intervention in overweight individuals with a family history of diabetes. *Diabetes Care* 1998;**21:**350-9.

Wolf AM, Conaway MR, Crowther JQ, Hazen KY, Nadler JL, Oneida B, et al. Translating lifestyle intervention to practice in obese patients with type 2 diabetes: Improving Control with Activity and Nutrition (ICAN) study. *Diabetes Care* 2004;**27:**1570-6.

Wylie-Rosett J, Swencionis C, Ginsberg M, Cimino C, Wassertheil-Smoller S, Caban A, et al. Computerized weight loss intervention optimizes staff time: the clinical and cost results of a controlled clinical trial conducted in a managed care setting. *Journal of the American Dietetic Association* 2001;**101:**1155-62; quiz 63-64.

Yeh MC, Rodriguez E, Nawaz H, Gonzalez M, Nakamoto D, Katz DL. Technical skills for weight loss: 2-y follow-up results of a randomized trial. *International Journal of Obesity* 2003;**27:**1500-6.

For peer review only

PRISMA 2020 Checklist

Section and Topic	Item #	Checklist item	Location where item is reported
**TITLE**			
Title	1	Identify the report as a systematic review.	Title page (page 1)
**ABSTRACT**			
Abstract	2	See the PRISMA 2020 for Abstracts checklist.	Page 2 (and PRISMA abstracts checklist provided)
**INTRODUCTION**			
Rationale	3	Describe the rationale for the review in the context of existing knowledge.	5 to 6
Objectives	4	Provide an explicit statement of the objective(s) or question(s) the review addresses.	6
**METHODS**			
Eligibility criteria	5	Specify the inclusion and exclusion criteria for the review and how studies were grouped for the syntheses.	6 to 7
Information sources	6	Specify all databases, registers, websites, organisations, reference lists and other sources searched or consulted to identify studies. Specify the date when each source was last searched or consulted.	7 and Appendix 1
Search strategy	7	Present the full search strategies for all databases, registers and websites, including any filters and limits used.	Appendix 1
Selection process	8	Specify the methods used to decide whether a study met the inclusion criteria of the review, including how many reviewers screened each record and each report retrieved, whether they worked independently, and if applicable, details of automation tools used in the process.	7
Data collection process	9	Specify the methods used to collect data from reports, including how many reviewers collected data from each report, whether they worked independently, any processes for obtaining or confirming data from study investigators, and if applicable, details of automation tools used in the process.	7 to 9
Data items	10a	List and define all outcomes for which data were sought. Specify whether all results that were compatible with each outcome domain in each study were sought (e.g. for all measures, time points, analyses), and if not, the methods used to decide which results to collect.	7 to 9
	10b	List and define all other variables for which data were sought (e.g. participant and intervention characteristics, funding sources). Describe any assumptions made about any missing or unclear information.	7 to 9
Study risk of bias assessment	11	Specify the methods used to assess risk of bias in the included studies, including details of the tool(s) used, how many reviewers assessed each study and whether they worked independently, and if applicable, details of automation tools used in the process.	Not applicable as the focus of this analysis is not of the effects of the intervention. For this, the reader is referred to the main REBALANCE paper for details of the risk of bias

For peer review only - http://bmjopen.bmj.com/site/about/guidelines.xhtml

PRISMA 2020 Checklist

Section and Topic	Item #	Checklist item	Location where item is reported
			methods and results of the assessment
Effect measures	12	Specify for each outcome the effect measure(s) (e.g. risk ratio, mean difference) used in the synthesis or presentation of results.	7 to 9
Synthesis methods	13a	Describe the processes used to decide which studies were eligible for each synthesis (e.g. tabulating the study intervention characteristics and comparing against the planned groups for each synthesis (item #5)).	8 to 9
	13b	Describe any methods required to prepare the data for presentation or synthesis, such as handling of missing summary statistics, or data conversions.	8 to 9
	13c	Describe any methods used to tabulate or visually display results of individual studies and syntheses.	8 to 9
	13d	Describe any methods used to synthesize results and provide a rationale for the choice(s). If meta-analysis was performed, describe the model(s), method(s) to identify the presence and extent of statistical heterogeneity, and software package(s) used.	8 to 9
	13e	Describe any methods used to explore possible causes of heterogeneity among study results (e.g. subgroup analysis, meta-regression).	NA for descriptive statistics
	13f	Describe any sensitivity analyses conducted to assess robustness of the synthesized results.	NA
Reporting bias assessment	14	Describe any methods used to assess risk of bias due to missing results in a synthesis (arising from reporting biases).	NA
Certainty assessment	15	Describe any methods used to assess certainty (or confidence) in the body of evidence for an outcome.	NA for descriptive statistics
**RESULTS**			
Study selection	16a	Describe the results of the search and selection process, from the number of records identified in the search to the number of studies included in the review, ideally using a flow diagram.	10 and Appendix 2
	16b	Cite studies that might appear to meet the inclusion criteria, but which were excluded, and explain why they were excluded.	No studies were excluded. The dataset comprised 131 RCTs included in a systematic review of weight management interventions for adults with BMI ≥35kg/m2, as part of the REBALANCE project (NIHR

For peer review only

PRISMA 2020 Checklist

Section and Topic	Item #	Checklist item	Location where item is reported
			HTA15/09/04). All 131 RCTs were included in this analysis.
Study characteristics	17	Cite each included study and present its characteristics.	10 and Appendix 3
Risk of bias in studies	18	Present assessments of risk of bias for each included study.	NA (see response to item 11)
Results of individual studies	19	For all outcomes, present, for each study: (a) summary statistics for each group (where appropriate) and (b) an effect estimate and its precision (e.g. confidence/credible interval), ideally using structured tables or plots.	14, 16, 18
Results of syntheses	20a	For each synthesis, briefly summarise the characteristics and risk of bias among contributing studies.	12, 15, 17
	20b	Present results of all statistical syntheses conducted. If meta-analysis was done, present for each the summary estimate and its precision (e.g. confidence/credible interval) and measures of statistical heterogeneity. If comparing groups, describe the direction of the effect.	10 to 18
	20c	Present results of all investigations of possible causes of heterogeneity among study results.	NA
	20d	Present results of all sensitivity analyses conducted to assess the robustness of the synthesized results.	NA
Reporting biases	21	Present assessments of risk of bias due to missing results (arising from reporting biases) for each synthesis assessed.	NA
Certainty of evidence	22	Present assessments of certainty (or confidence) in the body of evidence for each outcome assessed.	NA
**DISCUSSION**			
Discussion	23a	Provide a general interpretation of the results in the context of other evidence.	19
	23b	Discuss any limitations of the evidence included in the review.	20
	23c	Discuss any limitations of the review processes used.	23
	23d	Discuss implications of the results for practice, policy, and future research.	23, 24
**OTHER INFORMATION**			
Registration and protocol	24a	Provide registration information for the review, including register name and registration number, or state that the review was not registered.	3
	24b	Indicate where the review protocol can be accessed, or state that a protocol was not prepared.	7
	24c	Describe and explain any amendments to information provided at registration or in the protocol.	NA
Support	25	Describe sources of financial or non-financial support for the review, and the role of the funders or sponsors in the review.	39-40
Competing interests	26	Declare any competing interests of review authors.	39
Availability of data, code and other materials	27	Report which of the following are publicly available and where they can be found: template data collection forms; data extracted from included studies; data used for all analyses; analytic code; any other materials used in the review.	40

For peer review only - http://bmjopen.bmj.com/site/about/guidelines.xhtml

From: Page MJ, McKenzie JE, Bossuyt PM, Boutron I, Hoffmann TC, Mulrow CD, et al. The PRISMA 2020 statement: an updated guideline for reporting systematic reviews. BMJ 2021;372:n71. doi: 10.1136/bmj.n71

For peer review only

PRISMA 2020 Checklist

For more information, visit: http://www.prisma-statement.org/

For peer review only

PRISMA 2020 for Abstracts Checklist

Section and Topic	Item #	Checklist item	Reported (Yes/No)
**TITLE**			
Title	1	Identify the report as a systematic review.	Y
**BACKGROUND**			
Objectives	2	Provide an explicit statement of the main objective(s) or question(s) the review addresses.	Y
**METHODS**			
Eligibility criteria	3	Specify the inclusion and exclusion criteria for the review.	Y
Information sources	4	Specify the information sources (e.g. databases, registers) used to identify studies and the date when each was last searched.	Y
Risk of bias	5	Specify the methods used to assess risk of bias in the included studies.	Not applicable as the focus of this analysis is not of the effects of the intervention. For this, the reader is referred to the main REBALANCE paper for details of the risk of bias methods and results of the risk of bias assessment.
Synthesis of results	6	Specify the methods used to present and synthesise results.	Y
**RESULTS**			

For peer review only - http://bmjopen.bmj.com/site/about/guidelines.xhtml

PRISMA 2020 for Abstracts Checklist

Section and Topic	Item #	Checklist item	Reported (Yes/No)
Included studies	7	Give the total number of included studies and participants and summarise relevant characteristics of studies.	Y
Synthesis of results	8	Present results for main outcomes, preferably indicating the number of included studies and participants for each. If meta-analysis was done, report the summary estimate and confidence/credible interval. If comparing groups, indicate the direction of the effect (i.e. which group is favoured).	Y
**DISCUSSION**			
Limitations of evidence	9	Provide a brief summary of the limitations of the evidence included in the review (e.g. study risk of bias, inconsistency and imprecision).	Y
Interpretation	10	Provide a general interpretation of the results and important implications.	Y
**OTHER**			
Funding	11	Specify the primary source of funding for the review.	Y
Registration	12	Provide the register name and registration number.	Y

From: Page MJ, McKenzie JE, Bossuyt PM, Boutron I, Hoffmann TC, Mulrow CD, et al. The PRISMA 2020 statement: an updated guideline for reporting systematic reviews. BMJ 2021;372:n71. doi: 10.1136/bmj.n71

For more information, visit: http://www.prisma-statement.org/

For peer review only

Does weight management research for adults with severe obesity represent them? Analysis of systematic review data.

Journal:	*BMJ Open*
Manuscript ID	bmjopen-2021-054459.R2
Article Type:	Original research
Date Submitted by the Author:	29-Apr-2022
Complete List of Authors:	Robertson, Clare; University of Aberdeen, Health Services Research Unit<br>Aceves-Martins, Magaly; University of Aberdeen, Health Services Research Unit<br>Cruickshank, Moira; University of Aberdeen, Health Services Research Unit<br>Imamura, Mari; University of Aberdeen, Health Services Research Unit<br>Avenell, Alison; University of Aberdeen, Health Services Research Unit
**Primary Subject Heading**:	Health services research
Secondary Subject Heading:	Health policy, Public health
Keywords:	PUBLIC HEALTH, Clinical trials < THERAPEUTICS, Health policy < HEALTH SERVICES ADMINISTRATION & MANAGEMENT

I, the Submitting Author has the right to grant and does grant on behalf of all authors of the Work (as defined in the below author licence), an exclusive licence and/or a non-exclusive licence for contributions from authors who are: i) UK Crown employees; ii) where BMJ has agreed a CC-BY licence shall apply, and/or iii) in accordance with the terms applicable for US Federal Government officers or employees acting as part of their official duties; on a worldwide, perpetual, irrevocable, royalty-free basis to BMJ Publishing Group Ltd ("BMJ") its licensees and where the relevant Journal is co-owned by BMJ to the co-owners of the Journal, to publish the Work in this journal and any other BMJ products and to exploit all rights, as set out in our licence.

The Submitting Author accepts and understands that any supply made under these terms is made by BMJ to the Submitting Author unless you are acting as an employee on behalf of your employer or a postgraduate student of an affiliated institution which is paying any applicable article publishing charge ("APC") for Open Access articles. Where the Submitting Author wishes to make the Work available on an Open Access basis (and intends to pay the relevant APC), the terms of reuse of such Open Access shall be governed by a Creative Commons licence – details of these licences and which Creative Commons licence will apply to this Work are set out in our licence referred to above.

Other than as permitted in any relevant BMJ Author's Self Archiving Policies, I confirm this Work has not been accepted for publication elsewhere, is not being considered for publication elsewhere and does not duplicate material already published. I confirm all authors consent to publication of this Work and authorise the granting of this licence.

For peer review only

Does weight management research for adults with severe obesity represent them? Analysis of systematic review data.

Clare Robertson (ORCiD ID 0000-0001-6019-6795), Magaly Aceves-Martins (ORCiD ID 0000-0002-9441-142X), Moira Cruickshank (ORCiD ID 0000-0002-5182-884X), Mari Imamura (ORCiD ID 0000-0003-4871-0354), Alison Avenell (ORCiD ID 0000-0003-4813-5628)

Clare Robertson, Research Fellow, Health Services Research Unit, University of Aberdeen, Aberdeen, UK

Magaly Aceves-Martins, Research Fellow, Health Services Research Unit, University of Aberdeen, Aberdeen, UK, The Rowett Institute, University of Aberdeen, Aberdeen, UK

Moira Cruickshank, Research Fellow, Health Services Research Unit, University of Aberdeen, Aberdeen, UK

Mari Imamura, Research Fellow, Health Services Research Unit, University of Aberdeen, Aberdeen, UK

Alison Avenell, Professor, Health Services Research Unit, University of Aberdeen, Aberdeen, UK

Corresponding author: Clare Robertson, Health Services Research Unit, University of Aberdeen, Health Sciences Building, Foresterhill, Aberdeen, UK, Telephone: +44 (0) 1224 438086, Email: c.robertson@abdn.ac.uk

Manuscript word count: 3999

For peer review only

ABSTRACT

Objective

Our objective was to determine the extent to which current evidence from long-term randomised controlled trials (RCTs) of weight management are generalisable and applicable to under-served adult groups with obesity (body mass index [BMI] ≥35kg/m^2).

Methods

Descriptive analysis of 131 RCTs, published after 1990 to May 2017 with ≥1 year of follow-up, included in a systematic review of long-term weight management interventions, for adults with BMI ≥35kg/m^2 (the REBALANCE project). Studies were identified from MEDLINE, EMBASE, PsychINFO, SCI, CENTRAL, and from hand-searching. Reporting of trial inclusion and exclusion criteria, trial recruitment strategies, baseline characteristics, and outcomes were analysed using a pre-defined list of characteristics informed by the PROGRESS (Place of residence, Race/ethnicity/culture/language, Occupation, Gender/sex, Religion, Education, Socioeconomic status, Social capital) -Plus framework and the UK Equality Act 2010.

Results

Few (6.1%) trials reported adapting recruitment to appeal to under-served groups. 10.0% reported culturally adapting their trial materials. Only 6.1% of trials gave any justification for their exclusion criteria, yet over half excluded participation for age or mental health reasons. Just over half (58%) of the trials reported participants' race or ethnicity, and a fifth reported socioeconomic status. Where outcomes were reported for under-served groups, the most

common analysis was by sex (47.3%) followed by race or ethnicity (16.8%). 3.1% of trials reported outcomes according to socioeconomic status.

Discussion

Although we were limited by poor trial reporting, our results indicate inadequate representation of people most at risk of obesity. Guidance for considering under-served groups may improve the appropriateness of research and inform greater engagement with health and social care services.

Funding

National Institute for Health Research Health Technology Assessment Programme (Project number: 15/09/04).

Systematic review registration

PROSPERO CRD42016040190

For peer review only - http://bmjopen.bmj.com/site/about/guidelines.xhtml

ARTICLE SUMMARY

Strengths and limitations of this study

- A unique analysis exploring whether randomised trials evaluating interventions for weight management for people with severe obesity consider the needs of under-served groups
- Data set includes up-to-date, best available randomised controlled trial evidence (considered to be the highest level of evidence to inform guidelines and clinical practice) identified using robust and exhaustive literature search strategies
- Data set includes wide-ranging weight management interventions delivered in different settings, from an international perspective
- Our analysis was limited by poor reporting of whether subgroup reporting by under-served groups was planned and whether trials were adequately powered to detect subgroup differences
- Non-randomised study designs, interventions with shorter follow-up, and unpublished studies may have been more inclusive in their designs and reporting

y only

For peer review only - http://bmjopen.bmj.com/site/about/guidelines.xhtml

Background

In high-income countries, and increasingly in low and middle-income countries, lower incomes, less education, lower socioeconomic status, and disability are associated with greater risk of obesity for adults.[1-5] While the underlying causes of obesity are varied, there is an increasing association between obesity and deprivation that is driving poorer health outcomes and increasing health inequalities.[6] For example, most of these risk factors are stronger for women than men, [1-3,7] although men with obesity may be less likely than women to undertake weight management programmes.[8] Being an adult with obesity is associated with a lower health-related quality of life than a healthy weight adult of the same socioeconomic status.[9] More severe obesity, such as BMI ≥35kg/m^2 or more, with its associated greater risks for comorbidities, reduced quality of life and premature mortality [10] is particularly related to lower socioeconomic status, [1, 11] and intellectual and physical disabilities.[4] Poorer outcomes from COVID-19 are also strongly related to obesity, particularly severe obesity.[5] For countries such as the US, UK, Australia and New Zealand, some racial or ethnic groups may also be at much greater risk of obesity, especially severe obesity. [11- 14]

Preventing obesity and providing effective interventions, particularly for people with more severe obesity, is, therefore, a major public health challenge and vital in terms of addressing health inequalities. While organisations such as the U.S. Food and Drug Administration (FDA),[15-18] the National Institutes of Health (NIH)[15] and the National Institutes of Health Research (NIHR)[19] have produced guidelines on the inclusion of individuals of all ages, sexes/genders, races and ethnicities, and other physical, sensory/perceptual, cognitive and emotional characteristics, there is a lack of accessible policy-ready evidence on what works in terms of interventions to reduce inequalities in obesity. It is also recognised that some

For peer review only - http://bmjopen.bmj.com/site/about/guidelines.xhtml

groups, (for example, socially disadvantaged, less educated, and minority race or ethnic groups) may be less likely to be recruited into randomised controlled trials (RCTs) for lifestyle interventions.[20-24] Similarly, religion [25] and sexual orientation [26, 27] have been linked to weight and body image. It is, therefore, important to understand the extent to which the current evidence base is applicable to those who are most at risk of experiencing poorer obesity-related health outcomes and have more severe obesity.

This study aimed to determine the extent to which the findings from intervention studies of weight management, as exemplified by long-term randomised controlled trials (RCTs), are generalisable and applicable to those most at risk, particularly under-served groups with severe obesity. To examine these questions, we set out:

1. to describe inclusion and exclusion criteria for RCTs of adult weight management interventions, and in those trials:
2. to describe efforts to tailor recruitment strategies to improve recruitment of people from under-served groups,
3. to describe efforts to culturally adapt interventions to increase the accessibility or appeal to shared characteristics of an under-served group,
4. to describe reported baseline characteristics and outcomes for these groups.

Methods

Our dataset comprised 131 RCTs included in a systematic review of weight management interventions for adults with BMI ≥35kg/m^2, as part of the REBALANCE project (NIHR HTA15/09/04).[10] BMI ≥35kg/m^2 was chosen as this is a cut-off often used for accessing bariatric surgery or secondary care weight management clinical services in the UK. Eligible interventions included diet (including, but not limited to, very low calorie diets and meal

For peer review only - http://bmjopen.bmj.com/site/about/guidelines.xhtml

replacements), lifestyle (including combinations of diet, physical activity, and types of counselling), bariatric surgery, or orlistat. RCTs were restricted to publications after 1990 up to May 2017 to reflect more recent clinical practice. Literature searching was conducted in June 2016 and updated in April/May 2017. Details of the literature search method and search strategy are available in Appendix 1 of the supplementary files. Trials had to report long-term data on weight change (≥ 1 year of follow-up) and include trial populations with a baseline mean or median BMI $\geq 35 kg/m^2$. The decision to focus on long-term RCTs for this study was informed by the preference for high-quality, long-term evidence of lasting effectiveness in guideline documents [8, 28- 31] and are, therefore, most likely to influence treatment policy decisions. Reports published as abstracts or conference proceedings only were excluded. Three reviewers screened titles, abstracts and full text reports with a 10% quality assessment check. We attempted to contact the first, second and last authors of the main publications to identify all additional materials (i.e. protocols, trial materials and diet books) to inform our data extraction for the main REBALANCE report. Full details of the completed REBALANCE project, including the protocol, have been published.[10]

In the absence of definitions of under-served groups, we identified under-served groups by using protected characteristics informed by the PROGRESS-Plus framework[32] and the UK Equality Act 2010.[33] Four reviewers (MA-M, MC, MI and CR) conducted double data coding of each RCT for their reporting of whether trials reported details of their inclusion and exclusion criteria, trial recruitment strategies, baseline characteristics, and outcome reporting for the following characteristic groups, with disagreements resolved by consensus:

- Older age
- Physical health

For peer review only - http://bmjopen.bmj.com/site/about/guidelines.xhtml

- Mental health (including, but not limited to, depression, psychosis, schizophrenia, substance abuse and eating disorders)
- Comorbidities (e.g. Types 1 and 2 diabetes mellitus)
- Gender/sex (including RCTs recruiting only men or women)
- Sexual orientation
- Gender reassignment
- Marriage or civil partnership status
- Pregnancy
- Religion or belief
- Place of residence/housing (including residents of supported accommodation and homeowner status)
- Race or ethnicity
- Language
- Occupational status
- Education/literacy
- Socioeconomic status (SES), including individual SES and participants recruited from rural or disadvantaged geographical locations
- Social capital (including social support networks and/or social isolation)
- PROGRESS Plus (personal characteristics associated with discrimination (e.g. age, disability), features of relationships (e.g. smoking parents, excluded from school), time-dependent relationships (e.g. leaving the hospital, respite care, other instances where a person may be temporarily at a disadvantage)

For inclusion and exclusion criteria, trials were coded by pre-defined categories indicating whether any of the characteristic groups were clearly reported in the inclusion/exclusion

For peer review only - http://bmjopen.bmj.com/site/about/guidelines.xhtml

For peer review only

criteria, or, where details were not reported, whether the setting of the trial encouraged/discouraged inclusion of individuals from a particular characteristic group (e.g. recruitment was set in a health centre predominantly serving people from a characteristic group), or by whether it was unclear that the trial included/excluded people from any of the characteristic groups. For baseline and outcome reporting, we coded whether the protected characteristic was reported and, if reported, whether it was reported for individual treatment groups or the trial population as a whole. Where subgroup analyses were reported, we coded these according to whether it was clear/unclear from the study report that analyses were pre-planned. For a trial to be coded as having adapted their recruitment strategy for an under-served group, additional efforts to employ strategies that would appeal to that particular group (e.g. held recruitment days in particular settings or developed recruitment materials in multiple languages) had to be demonstrated. Trials that solely recruited from one characteristic group using conventional recruitment methods (e.g. newspaper or radio advertisements) were not coded as having an adapted recruitment strategy. Similarly, trials had to demonstrate that their interventions were designed with an under-served group in mind to be coded as having delivered a culturally adapted intervention. The focus of this study was to provide a description of trial methods and trial reporting to answer each of our research questions in relation to under-served groups; therefore, no formal statistical analysis was conducted.

Ethics approval

Ethical approval was not required.

Patient and public involvement

For peer review only - http://bmjopen.bmj.com/site/about/guidelines.xhtml

Although we did not consult patient representatives for this particular analysis, three patient representatives were members of the REBALANCE project Advisory Group, who contributed to developing the research questions, data interpretation and reporting of the research findings.

Results

From the total of 131 included trials, 19 were identified from database searching and 112 were identified from autoalert searching. The PRISMA (Preferred Reporting Items for Systematic Reviews and Meta-Analyses) flow chart and list of included studies are presented in Appendices 2 and 3 of the supplementary files. Of the 131 trials, 41 (31.3%) provided us with additional materials for their publications for the main REBALANCE report, although most of the information for the current analysis was obtained from the primary publications. The majority (81/131, 61.8%) of included studies were set in North America (80 in the USA and 1 in the USA and Canada), 41 out of 131 (31.3%) were in European countries (including 8 in the UK), 8 (6.1%) were in the Southern Hemisphere (six in Australia, one in New Zealand and one in Australia and New Zealand) and one in Brazil. None of the trials were set in low-income countries. Just under half (62/131, 47.3%) of the studies were published between 2011 and 2017. Five (3.8%) trials were linked to publications reporting qualitative data.[34-38] Few trials had follow-up durations longer than 12 months, with the exceptions of the US Look AHEAD trial [39] (median duration of 9.6 years), and four trials with follow-up times of five years. [40-43] The Look AHEAD trial was the largest trial, including over 5000 participants. Interventions were wide ranging, including very low calorie (19/131, 14.5%), orlistat (12/131 9.2%), bariatric surgery (11/131, 8.4%) and other lifestyle weight management programmes incorporating diet and physical activity advice (89/131, 67.9%).

For peer review only - http://bmjopen.bmj.com/site/about/guidelines.xhtml

Details of the characteristics of the included studies can be found in the supplementary files of the REBALANCE report.[10]

Trial recruitment

More than half of the trials (71/131, 54%) recruited participants either solely or partially through a health service provider, for example, either solely from outpatient clinics and general practices,[44, 45] or by physician referral and targeted mailing.[46] Recruitment methods were unclear or not reported in 14 trials (10.7%).[47-59] Recruitment methods for the other trials were mainly advertisements in local newspapers or other media. Based on their reporting, only three (2.3%) trials were judged to have adapted their recruitment strategies to appeal to under-served groups.[36, 60, 61] These pre-planned strategies included holding pre-recruitment presentations in US schools,[60] recruitment events at football stadiums of Scottish Premier League football clubs [36] and having bilingual staff take informed consent and provide written consent forms in both English and Spanish languages.[61] It was unclear in a further five (3.8%) trials whether recruitment strategies had been adapted beyond conventional methods.[53-55,62, 63]

Regarding adaptions to interventions, seven trials (5.3%, six from the US and one from New Zealand) [61, 63-68] recruited participants from diverse racial or ethnic groups and reported cultural adaptations to their interventions. Five (3.8%) of these trials [63-67] included advice on regional or culturally adapted recipes and foods for specific ethnic groups. Two trials (1.5%) [61, 68] had interventions that were delivered by bilingual staff. One trial [68] also reported that the intervention was designed for delivery in populations with limited literacy and numeracy and impaired access to health-promoting resources. While two trials [52, 69] recruited participants from workplace settings (an automobile manufacturer and a university; both were not

For peer review only - http://bmjopen.bmj.com/site/about/guidelines.xhtml

considered to meet the PROGRESS-Plus occupation definition), and one trial recruited married participants,[70] the trials did not report any attempts to alter their recruitment strategies or interventions to appeal to under-served occupation or social capital groups. The trials did not report recruitment strategies or adaptions to interventions for sexual orientation.

A further five trials (all from the US) [71-75] sought recruitment from specific racial or ethnic groups and reported intervention adaptations to increase cultural salience. Four of these trials [71-74] included culturally specific dietary advice and recipes. One trial described including bilingual interventions [71] and three trials described including interventionists from specific racial or ethnic groups.[75,73,74] Two trials [73, 74] described using logos and programme identification "for African-Americans", with one of these trials [73] including a video greeting from an African-American principal investigator.

The number of trials reporting inclusion and exclusion criteria by protected characteristic groups is presented in Table 1. Four older trials (3.1%) [47, 52, 76,77] did not report any inclusion criteria, and inclusion criteria was unclear in one further study.[42] Seven (5.3%) [52, 70, 78-82] trials did not report any explicit exclusion criteria and did not report that they had no exclusion criteria. Eight (6.1%) trials reported either full [36, 38, 45, 72, 83, 84] or partial [60, 85] justification for their exclusion criteria. Justification for exclusion criteria included prevention of poor adherence and losses to follow-up, [38, 45, 84, 85] such as substance abuse, mental health problems, or cognitive impairment (that might, in the opinion of the investigators, hinder participation), lower BMI cut-offs for Asian people,[60] non-English language speakers where the intervention required English language comprehension,[84] influence of pregnancy and breastfeeding on weight,[84] taking medications that influence weight,[72] and contraindications or safety concerns associated with participating in the

For peer review only - http://bmjopen.bmj.com/site/about/guidelines.xhtml

intervention (e.g. risk of participants with cardiovascular disease participating in exercise programmes).[36, 45, 84, 85] Over half (58.0%) of the trials reported excluding people with mental health conditions and 44.2% excluded people with substance abuse or addiction issues. The majority of trials also excluded adults from older age groups and based on current or planned pregnancy.

Twenty-one (16.0%) trials were judged to have inclusion criteria that might have implicitly excluded certain disadvantaged groups, such as people who do not have healthcare insurance,[69, 79] people who do not belong to a particular religious community group,[87] people without regular internet [35] or telephone [88] access, and English language comprehension.[44, 45, 49, 56, 60, 68, 89-96] In a further 40 (30.5%) trials, it was unclear if trial recruitment could have implicitly excluded disadvantaged groups. Few trials reported their inclusion criteria so as to include particular under-served groups or were judged to have made efforts to maximise trial recruitment from these groups. When trial recruitment was targeted, this was usually to recruit women only (19 trials) or people with Type 2 diabetes (28 trials).

For peer review only - http://bmjopen.bmj.com/site/about/guidelines.xhtml

Table 1 The number (and percent) of trials (n=131) reporting inclusion and exclusion criteria by protected characteristics included in the REBALANCE systematic review of RCTs

	Inclusion			Exclusion
	Protected characteristic is reported in the inclusion criteria, n (%)	Protected characteristic is not reported in inclusion criteria, but an effort was made to recruit from the protected characteristic group, n (%)	Unclear if the protected characteristic was targeted for inclusion or if the trial unintentionally recruited solely/mainly from the protected characteristic group, n (%)	Protected characteristic is reported in the exclusion criteria, or the reported inclusion criteria clearly excluded the protected characteristic, n (%)
Place of residence/housing	5 (3.8%)	0	0	0
Race/ethnicity	7 (5.3%)	5 (3.8%)	2 (1.5%)	1 (0.8%)
Occupation status	0	0	0	0
Women only	19 (14.5%)	0	4 (3.1%)	0
Pregnancy	0	0	0	72 (54.9%)
Men only	4 (3.0%)	0	1 (0.76%)	5 (3.8%)
Religion/belief	1 (0.8%)			
Education/literacy	0	0	0	0
Socioeconomic status	3 (2.3%)	3 (2.3%)	0	0
Marital status	1 (0.8%)	0	0	1 (0. 8%)
Older age	2 (1.5%)	1 (0.8%)	0	82 (62.6%)[a]
Physical health	10 (7.6%)	0	0	51 (38.9%)
Diabetes Type 1	0	0	0	15 (11.5%)
Diabetes Type 2	28 (21.4%)	0	0	29 (22.1%)
Diabetes (Type 1 and 2 or Type not reported)	0	0	0	3 (2.3%)
Mental health	6 (4.6%)	0	0	76 (58.0%)
Substance abuse	0	0	0	58 (44.2%)
Eating disorder	0	0	0	35 (26.7%)
Language	0	0	0	16 (12.2%)

a. includes 8 RCTs recruiting participants up to 75 years, 1 RCT recruited participants up to 76 years and 3 RCTs recruited participants aged up to 80 years

For peer review only - http://bmjopen.bmj.com/site/about/guidelines.xhtml

Reporting of baseline characteristics

Details of the number of trials reporting baseline characteristics of their participants by each of the protected characteristic groups are presented in Table 2. The majority of trials reported age (99.2%) and sex (97.7%) in their description of baseline participant characteristics. Just over half of the trials (58.0%) reported race or ethnicity. Education history was less well-reported (40.5%). Socioeconomic status was reported by 22.9%, and occupation status by 21.4%. Of the trials that were not specifically for people with diabetes, six (4.6%) included diabetes in their reporting of baseline characteristics. Two (1.5%) trials reported whether people lived alone or not (coded as social capital).[97, 98] Few trials reported details of the other protected characteristics, and none reported details of gender reassignment, sexual orientation or pregnancy.

For peer review only - http://bmjopen.bmj.com/site/about/guidelines.xhtml

Table 2 The number (and percent) of trials (n=131) reporting protected characteristics at baseline in the REBALANCE systematic review of RCTs

	**Protected characteristic is reported at baseline for each intervention arm**	**Protected characteristic is reported at baseline for the whole trial**	**Total**
Age	126 (96.2%)	4 (3.0%)	130 (99.2%)
Physical health	10 (7.6%)	-	10 (7.6%)
Mental health	10 (7.6%)	-	10 (7.6%)
Diabetes	6 (4.6%)	-	6 (4.6%)
Sex	126 (96.2%)	2 (1.5%)	128 (97.7%)
Gender reassignment	0	0	0
Sexual orientation	0	0	0
Marriage/civil partnership status	38 (29.0%)	-	38 (29.0%)
Pregnancy	0	0	0
Place of residence/housing	6 (4.6%)	-	6 (4.6%)
Occupation status	27 (20.6%)	1 (0.8%)	28 (21.4%)
Education/literacy	51 (38.9%)	2 (1.5%)	53 (40.5%)
Socioeconomic status	29 (22.1%)	1 (0.8%)	30 (22.9%)
Social capital	2 (1.5%)	-	2 (1.5%)
Religion/belief	1 (0.8%)	-	1 (0.8%)
Race/ethnicity	74 (56.5%)	2 (1.5%)	76 (58.0%)
PROGRESS Plus	2 (1.5%)	-	2 (1.5%)

For peer review only

For peer review only - http://bmjopen.bmj.com/site/about/guidelines.xhtml

Outcome reporting

Details of the number of trials reporting outcomes by each of the protected characteristics are shown in Table 3. Very few trials reported outcomes by protected characteristic groups. Where outcomes were reported by protected characteristics, the most common group was sex (47.3%) followed by race or ethnicity (16.8%).

For peer review only

For peer review only - http://bmjopen.bmj.com/site/about/guidelines.xhtml

Table 3 The number (and percent) of trials (n=131) reporting outcome data for protected characteristics in the REBALANCE systematic review of RCTs

	Trial recruitment was targeted at people from the protected characteristic group	One or more outcome(s) reported for the protected characteristic in planned subgroup analysis	One or more outcome(s) reported for the protected characteristic – unclear if subgroup analysis was pre-planned	Total
Older Age	2 (1.5%)[a]	2 (1.5%)	3 (2.3%)	7 (5.3%)
Physical health	10 (7.6%)	0	0	10 (7.6%)
Mental health	6 (4.6%)	0	2 (1.5%)	8 (6.1%)
Diabetes	28 (21.3%)	0	1 (0.8%)	29 (22.1%)
Sex	23 (17.5%) [b]	17 (13.0%)	22 (16.8%)	62 (47.3%)
Gender reassignment	0	0	0	0
Sexual orientation	0	0	0	0
Marriage/civil partnership status	1 (0.8%)	1 (0.8%)	3 (2.3%)	5 (3.8%)
Pregnancy	0	0	0	0
Place of residence/housing	0	2 (1.5%)	0	2 (1.5%)
Occupation status	2 (1.5%)	2 (1.5%)	4 (3.1%)	8 (6.1%)
Education/literacy	0	1 (0.8%)	6 (4.6%)	7 (5.3%)
Socioeconomic status	5 (3.8%)	4 (3.1%)	3 (2.3%)	12 (9.2%)
Social capital	1 (0.8%)	3 (2.3%)	2 (1.5%)	6 (4.6%)
Religion/belief	1 (0.8%)	0	0	1 (0.8%)
Race/ethnicity	8 (6.1%)	5 (3.8%)	9 (6.9%)	22 (16.8%)
PROGRESS Plus	0	0	0	0

a. Both trials recruited participants aged ≥65 years
b. 19 women only trials, 4 men only trials

For peer review only

Discussion

Main findings

Our findings demonstrate that most trialists testing weight management strategies to help adults with severe obesity fail to consider populations who are most at risk of poorer health outcomes. Almost all trials were from high-income countries, where lower socioeconomic status and income are associated with a greater prevalence of obesity, particularly severe obesity.[1-5] Few trials reported adapting recruitment to appeal to under-served groups or reported culturally adapting their trial materials for ethnic groups or people with limited English language literacy or numeracy. This is concerning as limiting the accessibility or appeal of trials could limit the representativeness of the trial population, and thus limit the generalisability of trial findings. Only 6.1% of trials gave any justification for their exclusion criteria, yet more than half excluded participation for age or mental health reasons. Where justification for exclusion was reported, the rationale included excluding people who were deemed likely to have poor intervention adherence or were more likely to be lost to follow-up, such as people with substance abuse, cognitive impairment, or mental health problems. Excluding these groups could lead to an unrealistic estimation of the real-world effectiveness of interventions. Just over half of the trials reported participants' race or ethnicity, and only around a fifth reported socioeconomic status. Where outcomes were reported for under-served groups, the most common analysis was by sex (47.3%) followed by race or ethnicity (16.8%); however, where analyses were presented as subgroups, it was often unclear whether these analyses were planned or unplanned. Similarly, some smaller trials might have been underpowered to detect differences in treatment effects between subgroups, but this was also unclear from trial reporting. This finding was also demonstrated by Liu and colleagues,[99] who highlighted a lack of transparent reporting of intentions to analyse race and ethnicity subgroups in Cochrane intervention reviews. Only 3.1% of the trials we reviewed reported

For peer review only - http://bmjopen.bmj.com/site/about/guidelines.xhtml

outcomes according to socioeconomic status. Few trials reported outcomes by the other protected characteristics.

Although we were limited by the available information in the published reports, our findings are concerning. In almost all trials, it is difficult to assess the generalisability of findings to the wider population of adults with severe obesity. There is clear evidence [1-5,11,12] that under-served groups with lower incomes, less education, lower socioeconomic status, intellectual and physical disabilities, or poorer mental health are more at risk of obesity, particularly severe obesity, in high-income countries, especially the US which provided the majority of trials examined. We do not have relevant data to be able to comment on the reasons for poor reporting. Nevertheless, the lack of reporting for characteristics reflecting under-served groups suggests that trial investigators did not consider, or faced barriers that prevented their inclusion in the design, recruitment, and analysis or reporting of their interventions.

Our finding that few trials adapted their recruitment methods or interventions to appeal to under-served groups suggests lack of engagement with under-served people with obesity in the design of services. This is important, given that a systematic review of qualitative research by Sutcliffe and colleagues [100] showed how service users have perspectives that should inform weight management services to improve their reach. From systematic reviews, researchers have clearly demonstrated the need to involve communities in all stages of research in order to enhance the engagement and generalisability of that research, acknowledging that this requires extended timeframes and greater costs.[21, 101] For example, Ni She and colleagues[102] undertook a rapid realist review of the mechanisms and resources needed to engage under-served, seldom-heard groups in health and social care research, with items grouped by an expert panel under the headings of environmental and social planning,

For peer review only - http://bmjopen.bmj.com/site/about/guidelines.xhtml

service provision, guidelines, fiscal measures, communication and marketing, and regulation and legislation. In the US, Arnegard and colleagues[103] have also called on the NIH's stakeholder groups to redouble their efforts to encourage sex- and gender-aware reporting of biomedical investigations. We endorse this call following the findings from our previous systematic review of weight management interventions for men with obesity.[8] Our review highlighted the paucity of evidence for men, who are less likely to take part in weight management interventions and the lack of engagement of men in all aspects of intervention design, and optimal trial recruitment processes of weight management. [8]

While the reasons for the under-representation of under-served groups in RCTs are likely to be complex and multifaceted, with many known and unknown barriers to participation, there is evidence that, for some groups, willingness to participate is not a predominant factor.[104] Mindful of the need to improve the engagement of under-served groups in research in the UK, the National Institute for Health Research set up the INCLUDE project, [19] which has led to the INCLUDE ethnicity framework; [105] providing four key questions on who should be involved in research, and how to facilitate involvement. Others have investigated and found a lack of external validity in trials for people with asthma,[106] type 2 diabetes,[107, 108] and neurological disease,[109] or a failure to justify exclusion criteria in trials of cardiovascular disease prevention [110] other than for safety reasons. These publications did not consider the socioeconomic status or educational attainment of trial participants. In a systematic review of 305 trials of clinical conditions, He and colleagues [111] found high exclusion rates in trials for people with hypertension (83.0%), lipid-lowering drugs in primary prevention (85.9%), type 2 diabetes (81.7%), COPD (84.3%) and asthma (96.0%), with no strong evidence that exclusion rates had changed with time.

For peer review only - http://bmjopen.bmj.com/site/about/guidelines.xhtml

More recently, others have also highlighted the small number of intervention studies testing out weight management for under-served adults,[112] or policies to assist socioeconomically disadvantaged groups.[113] A 2015 systematic review of interventions aimed at reducing socioeconomic inequalities for adults with obesity [114] found that primary care-delivered tailored weight loss programmes and group weight loss interventions had the most evidence of potential effectiveness in reducing obesity, at least in the short term, among low-income women, but there were few individual-level intervention studies and a lack of long term evidence of effectiveness.

Strengths and limitations

We used categories informed by the PROGRESS-PLUS framework [32] and the UK Equality Act 2010 [33] as the key characteristics for identifying those under-served participants that should be considered for study design, public patient involvement, recruitment, analysis and reporting, not just for trials of weight management, but trials generally. O'Neill[32] has shown how the prior PROGRESS framework can be used as an equity lens for systematic reviews and methodological studies; however, NIHR's INCLUDE project has recently published a more extensive list of categories of under-served groups to consider with regard to representation in trials.[115]

Our literature search attempted to identify all long-term randomised trials published since 1990 for adults with BMI $\geq$35kg/m^2 irrespective of the type of lifestyle intervention, including comparisons with orlistat and bariatric surgery. Although we included publications in any language from any country, we cannot exclude the possibility that we failed to find some trials, particularly those from low income countries, which might not be listed in the databases we searched.

For peer review only - http://bmjopen.bmj.com/site/about/guidelines.xhtml

While we originally contacted authors for all available additional materials relating to our main research question for the REBALANCE project,[10] we did not re-contact authors to obtain additional information relating to the current analysis. We were also limited by poor reporting by trial authors. Some trials were statistically underpowered to detect subgroup differences, and this might explain under-reporting of under-served characteristics; however, this was unclear from the trial reports. It is also possible that some trialists were unable to obtain relevant baseline data for some under-served groups if this was deemed sensitive by an ethics committee, e.g. sexual orientation. Nevertheless, we consider that most characteristics are pivotal to interpreting these trials into real-world guidance and services, so we would expect their presentation in trial publications, especially at baseline. We assessed long-term RCT evidence because it is most likely to inform guidance on weight management.[28-31] Other study designs, interventions with shorter follow-up, and unpublished studies may have been more inclusive in their designs and reporting.

Recommendations for research

Trialists should improve reporting of their justification of inclusion and exclusion criteria to meet current CONSORT statement guidelines,[116] and report sufficient data to allow comparisons between their populations and the populations for whom the interventions apply. Including core criteria for baseline reporting within the CONSORT checklist [116] could help to improve the completeness of reporting of these factors. NIHR's INCLUDE project's ethnicity framework provides important factors to consider with regard to ethnic groups (https://www.trialforge.org/trial-forge-centre/include/), which can help provide transferable considerations for other under-served groups. However, a wider equity lens may be needed in the face of groups with multiple disadvantages. Although guidance for research will aid

For peer review only - http://bmjopen.bmj.com/site/about/guidelines.xhtml

considerations of equity, we do not yet have ways of assessing when proportional representation in larger trials and sub-group reporting for under-served groups is insufficient. This research should be explicitly conducted with and for these under-served groups, ensuring user involvement in all stages of the research process.

Conclusions and recommendations for practice

Long-term RCTs of weight management in people with BMI ≥35mg^2 have inadequate representation of and engagement with under-served groups, who are particularly relevant for health and social care services. Thus, guidance for weight management research on how to improve the representation of under-served groups in clinical trials may improve the appropriateness of that research and help inform greater engagement of under-served communities with health and social care services.

For peer review only - http://bmjopen.bmj.com/site/about/guidelines.xhtml

REFERENCES

1. Booth HP, Charlton J, Gulliford MC. Socioeconomic inequality in morbid obesity with body mass index more than 40 kg/m^2 in the United States and England. *SSM Popul Health* 2017;3:172–178
2. Devaux M, Sassi F. Social inequalities in obesity and overweight in 11 OECD countries. *Eur J Public Health* 2013; 23(3):464–469
3. El-Sayed AM, Scarborough P, Galea S. Unevenly distributed: a systematic review of the health literature about socioeconomic inequalities in adult obesity in the United Kingdom. *BMC Public Health* 2012, 12:18
4. Hsieh K, Rimmer JH, Heller T. Obesity and associated factors in adults with intellectual disability. *J Intellect Disabil Res* 2014;58(9):851-863
5. Public Health England. Excess Weight and COVID-19 Insights from new evidence [Internet]. London: PHE publications; 2021 [cited February 2021]. Available from: https://assets.publishing.service.gov.uk/government/uploads/system/uploads/attachment_data/file/907966/PHE_insight_Excess_weight_and_COVID-19__FINAL.pdf
6. Holmes J. Tackling obesity: The role of the NHS in a whole-system approach. London: The Kings Fund 2021.
7. Grech A, Allman-Farinelli M. Prevalence and period trends of overweight and obesity in Australian young adults. *Eur J Clin Nutr* 2016; 70: 1083–1085
8. Robertson C, Archibald D, Avenell A, et al. Systematic reviews of and integrated report on the quantitative, qualitative and economic evidence base for the management of obesity in men. *Health Technol Assess* 2014;18(35).
9. Minet Kinge J, Morris S. Socioeconomic variation in the impact of obesity on health-related quality of life. *Soc Sci Med* 2010;71:1864e1871

For peer review only - http://bmjopen.bmj.com/site/about/guidelines.xhtml

10. Avenell A, Robertson C, Skea Z, et al. Bariatric surgery, lifestyle interventions and orlistat for severe obesity: the REBALANCE mixed-methods systematic review and economic evaluation. *Health Technol Assess* 2018;22(68).

11. Green MA, Rowe F. Explaining the widening distribution of Body Mass Index: A decomposition analysis of trends for England, 2002–2004 and 2012–2014. *Area* 2020;1–11

12. Ogden CL, Fryar CD, Martin CB, et al. Trends in Obesity Prevalence by Race and Hispanic Origin—1999-2000 to 2017-2018. *JAMA* 2020;324(12):1208–1210

13. Menigoz, K., Nathan, A. & Turrell, G. Ethnic differences in overweight and obesity and the influence of acculturation on immigrant bodyweight: evidence from a national sample of Australian adults. *BMC Public Health* 2016; 16, 932

14. New Zealand Ministry of Health. Obesity statistics. 2020. Available from https://www.health.govt.nz/nz-health-statistics/health-statistics-and-data-sets/obesity-statistics (Accessed October 2021)

15. U.S.Food and Drug Administration (FDA). Guidance for industry: collection of race and ethnicity data in clinical trials. https://www.fda.gov/media/75453/download Accessed March 2021

16. U.S.Food and Drug Administration (FDA). Evaluation and reporting of age-, race-, and ethnicity-specific data in medical device clinical studies. https://www.fda.gov/media/98686/download Accessed March 2021

17. U.S.Food and Drug Administration (FDA). Design considerations for devices intended for home use. https://www.fda.gov/media/84830/download Accessed March 2021

For peer review only - http://bmjopen.bmj.com/site/about/guidelines.xhtml

18. National Institutes of Health [internet]. U.S. 2019. Inclusion policies for research involving human subjects. https://grants.nih.gov/policy/inclusion.htm (accessed March 2021)

19. National Institute for Health Research [Internet]. London: The Institute; 2020 [cited February 2021]. Improving inclusion of under-served groups in clinical research: Guidance from the NIHR INCLUDE project. Available from: www.nihr.ac.uk/documents/improving-inclusion-of-under-served-groups-in-clinical-research-guidance-from-include-project/25435

20. Unger JM, Gralow JR, Albain KS, Ramsey SD, Hershman DL. Patient Income Level and Cancer Clinical Trial Participation: A Prospective Survey Study. *JAMA Oncol*. 2016;2(1):137-9.

21. Bonevski, B., Randell, M., Paul, C. et al. Reaching the hard-to-reach: a systematic review of strategies for improving health and medical research with socially disadvantaged groups. *BMC Med Res Methodol* 2014;14:42

22. Sateren W.B., Trimble E.L., Abrams J. et al. How Sociodemographics, Presence of Oncology Specialists, and Hospital Cancer Programs Affect Accrual to Cancer Treatment Trials. *J Clin Oncol* 2002 20:8, 2109-2117

23. Smart, A., Harrison, E. Under¬representation of minority ethnic groups in UK medical research. *Ethnicity and Health* 2017, 22: 65–82

24. Sardar MR, Badri M, Prince CT, Seltzer J, Kowey PR. Underrepresentation of Women, Elderly Patients, and Racial Minorities in the Randomized Trials Used for Cardiovascular Guidelines. *JAMA Intern Med*. 2014;174(11):1868–1870

25. Yeary, K. H. K., Sobal, J., and Wethington, E. Religion and body weight: a review of quantitative studies. *Obes Rev* 2017, 18: 1210– 122

For peer review only - http://bmjopen.bmj.com/site/about/guidelines.xhtml

26. de Souza P, Ciclitira KE. Men and Dieting: A Qualitative Analysis. *J Health Psychol* 2005;10(6):793-804

27. Gough B, Flanders G. Celebrating 'obese' bodies: Gay 'bears' talk about weight, body image and health. *Int J Mens Health* 2009;8:235–53

28. National Institute for Health and Care Excellence. Weight management: lifestyle services for overweight or obese adults. London; NICE 2014. URL https://www.nice.org.uk/guidance/ph53 (accessed May 2021)

29. National Health and Medical Research Council. Clinical practice guidelines for the management of overweight and obesity in adults, adolescents and children in Australia. Melbourne: National Health and Medical Research Council 2013

30. Hartmann-Boyce J, Johns D, Aveyard P, et al. Managing overweight and obese adults: update review. The clinical effectiveness of long-term weight management schemes for adults: Review 1a. Oxford: University of Oxford 2013

31. Hartmann-Boyce J, Johns D, Aveyard P, et al. How components of behavioural weight management programmes affect weight change: Review 1b. Oxford: University of Oxford 2013

32. O'Neill J, Tabish H, Welch V, et al. Applying an equity lens to interventions: using PROGRESS ensures consideration of socially stratifying factors to illuminate inequities in health. *J Clin Epidemiol* 2014, 67(1):56-64

33. Government Equalities Office, Equality and Human Rights Commission [Internet]. London: Government Digital Service; 2013 [cited February 2021]. Equality Act 2010: guidance. Available from: https://www.gov.uk/guidance/equality-act-2010-guidance

34. Yeh MC, Rodriguez E, Nawaz H, et al. Technical skills for weight loss: 2-y follow-up results of a randomized trial. *Int J Obes Relat Metab Disord* 2003;27:1500–6

For peer review only

For peer review only - http://bmjopen.bmj.com/site/about/guidelines.xhtml

35. Little P, Stuart B, Hobbs FR, et al. Randomised controlled trial and economic analysis of an internet-based weight management programme: POWeR+ (Positive Online Weight Reduction). *Health Technol Assess* 2017;21(4)

36. Hunt K, Gray CM, Maclean A, Smillie S, Bunn C, Wyke S. Do weight management programmes delivered at professional football clubs attract and engage high risk men? A mixed-methods study. *BMC Public Health* 2014;14:50

37. McRobbie H, Hajek P, Peerbux S, et al. Tackling obesity in areas of high social deprivation: clinical effectiveness and cost-effectiveness of a task-based weight management group programme – a randomised controlled trial and economic evaluation. *Health Technol Assess* 2016;20(79)

38. Green CA, Yarborough BJ, Leo MC, et al. Weight maintenance following the STRIDE lifestyle intervention for individuals taking antipsychotic medications. *Obesity* 2015;23:1995–2001

39. The Look AHEAD Research Group. Cardiovascular effects of intensive lifestyle intervention in type 2 diabetes. *N Engl J Med* 2013;369:145–54

40. Hakala P, Karvetti RL, Rönnemaa T. Group vs. individual weight reduction programmes in the treatment of severe obesity – a five year follow-up study. I*nt J Obes Relat Metab Disord* 1993;17:97–102

41. Hakala P. Weight reduction programmes at a rehabilitation centre and a health centre based on group counselling and individual support: short- and long-term follow-up study. *Int J Obes Relat Metab Disord* 1994;18:483–9

42. Mingrone G, Greco AV, Giancaterini A, Scarfone A, Castagneto M, Pugeat M. Sex hormone binding globulin levels and cardiovascular risk factors in morbidly obese subjects before and after weight reduction induced by diet or malabsorptive surgery. *Atherosclerosis* 2002;161:455–62

For peer review only - http://bmjopen.bmj.com/site/about/guidelines.xhtml

43. Schauer PR, Kashyap SR, Wolski K, Brethauer SA, Kirwan JP, Pothier CE, et al. Bariatric surgery versus intensive medical therapy in obese patients with diabetes. *N Engl J Med 2012*;366:1567–76

44. Bliddal H, Leeds AR, Stigsgaard L, Astrup A, Christensen R. Weight loss as treatment for knee osteoarthritis symptoms in obese patients: 1-year results from a randomised controlled trial. *Ann Rheum Dis* 2011;70:1798–803

45. Azar KM, Xiao L, Ma J. Baseline obesity status modifies effectiveness of adapted diabetes prevention program lifestyle interventions for weight management in primary care. *Biomed Res Int* 2013;2013:191209

46. Appel LJ, Clark JM, Yeh HC, Wang NY, Coughlin JW, Daumit G, et al. Comparative effectiveness of weight-loss interventions in clinical practice. *N Engl J Med* 2011;365:1959–68.

47. Agras WS, Berkowitz RI, Arnow BA, et al. Maintenance following a very-low-calorie diet. *J Consult Clin Psychol* 1996;64:610–13

48. Bakris G, Calhoun D, Egan B, Hellmann C, Dolker M, Kingma I, Orlistat and Resistant Hypertension Investigators. Orlistat improves blood pressure control in obese subjects with treated but inadequately controlled hypertension. *J Hypertens* 2002;20:2257–67

49. Bartels SJ, Pratt SI, Aschbrenner KA, et al. Clinically significant improved fitness and weight loss among overweight persons with serious mental illness. *Psychiatr Serv* 2013;64:729–36

50. Brehm BJ, Lattin BL, Summer SS, Boback JA, Gilchrist GM, Jandacek RJ, D'Alessio DA. One-year comparison of a high-monounsaturated fat diet with a high-carbohydrate diet in type 2 diabetes. *Diabetes Care* 2009;32:215–20

For peer review only - http://bmjopen.bmj.com/site/about/guidelines.xhtml

51. Broom I, Wilding J, Stott P, Myers N, UK Multimorbidity Study Group. Randomised trial of the effect of orlistat on body weight and cardiovascular disease risk profile in obese patients: UK Multimorbidity Study. *Int J Clin Pract* 2002;56:494–9

52. Dennison KF, Galante D, Dennison D, Golaszewski T. A one year post-program assessment of a computer-assisted instruction weight management program for industrial employees: lessons learned. *J Health Educ* 1996;27:38–43

53. Hauptman J, Lucas C, Boldrin MN, Collins H, Segal KR. Orlistat in the long-term treatment of obesity in primary care settings. *Arch Fam Med* 2000;9:160–7

54. Kahleova H, Hill M, Pelikanova T. Vegetarian vs. conventional diabetic diet – a 1-year follow-up. *Cor Vasa* 2014;56:e140–4

55. Kelley DE, Bray GA, Pi-Sunyer FX, et al. Clinical efficacy of orlistat therapy in overweight and obese patients with insulin-treated type 2 diabetes: a 1-year randomized controlled trial. *Diabetes Care* 2002;25:1033–41

56. Ströbl V, Knisel W, Landgraf U, Faller H. A combined planning and telephone aftercare intervention for obese patients: effects on physical activity and body weight after one year. *J Rehabil Med* 2013;45:198–205

57. Swinburn BA, Carey D, Hills AP, Hooper M, Marks S, Proietto J, et al. Effect of orlistat on cardiovascular disease risk in obese adults. *Diabetes Obes Metab* 2005;7:254–62

58. Wadden TA, Volger S, Sarwer DB, Vetter ML, Tsai AG, Berkowitz RI, et al. A two-year randomized trial of obesity treatment in primary care practice. *N Engl J Med* 2011;365:1969–79

59. Wing RR, Marcus MD, Salata R, Epstein LH, Miaskiewicz S, Blair EH. Effects of a very-low-calorie diet on long-term glycemic control in obese type 2 diabetic subjects. *Arch Intern Med* 1991;151:1334–40

For peer review only - http://bmjopen.bmj.com/site/about/guidelines.xhtml

60. Berry DC, Schwartz TA, McMurray RG, et al. The family partners for health study: a cluster randomized controlled trial for child and parent weight management. *Nutr Diabetes* 2014;4:e101

61. Van Name MA, Camp AW, Magenheimer EA, et al. Effective translation of an intensive lifestyle intervention for Hispanic women with prediabetes in a community health center setting. *Diabetes Care* 2016;39:525–31

62. Khoo J, Piantadosi C, Duncan R, Worthley SG, Jenkins A, Noakes M, et al. Comparing effects of a low-energy diet and a high-protein low-fat diet on sexual and endothelial function, urinary tract symptoms, and inflammation in obese diabetic men. J Sex Med 2011;8:2868–75

63. Kumanyika SK, Fassbender JE, Sarwer DB, et al. One-year results of the Think Health! study of weight management in primary care practices. *Obesity* 2012;20:1249–57

64. Krebs JD, Elley CR, Parry-Strong A, et al. The Diabetes Excess Weight Loss (DEWL) Trial: a randomised controlled trial of high-protein versus high-carbohydrate diets over 2 years in type 2 diabetes. *Diabetologia* 2012;55:905–14

65. Mayer-Davis EJ, D'Antonio AM, Smith SM, et al. Pounds off with empowerment (POWER): a clinical trial of weight management strategies for black and white adults with diabetes who live in medically underserved rural communities. *Am J Public Health* 2004;94:1736–42

66. Perri MG, Limacher MC, Durning PE, et al. Extended-care programs for weight management in rural communities: the Treatment of Obesity in Underserved Rural Settings (TOURS) randomized trial. *Arch Intern Med* 2008;168:2347–54

For peer review only - http://bmjopen.bmj.com/site/about/guidelines.xhtml

67. Perri MG, Limacher MC, von Castel-Roberts K, et al. Comparative effectiveness of three doses of weight-loss counseling: two-year findings from the rural LITE trial. *Obesity* 2014;22:2293–300

68. Bennett GG, Warner ET, Glasgow RE, et al. Obesity treatment for socioeconomically disadvantaged patients in primary care practice. *Arch Intern Med* 2012;172:565–74

69. Ostbye T, Stroo M, Brouwer RJ, et al. Steps to Health employee weight management randomized control trial: Short-term follow-up results. *J Occup Environ Med* 2015;57:188-95

70. Wing RR, Marcus MD, Epstein LH, Jawad A. A 'family-based' approach to the treatment of obese type II diabetic patients. J Consult Clin Psychol 1991;59:156–62

71. Poston WS, Reeves RS, Haddock CK, et al. Weight loss in obese Mexican Americans treated for 1-year with orlistat and lifestyle modification. *Int J Obes Relat Metab Disord* 2003;27:1486–93

72. Martin PD, Dutton GR, Rhode PC, Horswell RL, Ryan DH, Brantley PJ. Weight loss maintenance following a primary care intervention for low-income minority women. *Obesity* 2008;16:2462–7

73. Kumanyika SK, Shults J, Fassbender J, et al. Outpatient weight management in African-Americans: the Healthy Eating and Lifestyle Program (HELP) study. *Prev Med* 2005;41:488–502

74. Kumanyika SK, Wadden TA, Shults J, et al. Trial of family and friend support for weight loss in African American adults. *Arch Intern Med* 2009;169:1795–804

75. Fitzgibbon ML, Stolley MR, Schiffer L, Sharp LK, Singh V, Dyer A. Obesity Reduction Black Intervention Trial (ORBIT): 18-month results. *Obesity* 2010;18:2317–25

For peer review only - http://bmjopen.bmj.com/site/about/guidelines.xhtml

76. Wadden TA, Berkowitz RI, Vogt RA, Steen SN, Stunkard AJ, Foster GD. Lifestyle modification in the pharmacologic treatment of obesity: a pilot investigation of a potential primary care approach. *Obes Res* 1997;5:218–26

77. Wadden TA, Vogt RA, Foster GD, Anderson DA. Exercise and the maintenance of weight loss: 1-year follow-up of a controlled clinical trial. *J Consult Clin Psychol* 1998;66:429–33

78. Torgerson JS, Lissner L, Lindroos AK, Kruijer H, Sjöström L. VLCD plus dietary and behavioural support versus support alone in the treatment of severe obesity. A randomised two-year clinical trial. *Int J Obes Relat Metab Disord* 1997;21:987–94

79. Melin I, Karlström B, Lappalainen R, Berglund L, Mohsen R, Vessby B. A programme of behaviour modification and nutrition counselling in the treatment of obesity: a randomised 2-y clinical trial. *Int J Obes Relat Metab Disord* 2003;27:1127–35

80. Pascale RW, Wing RR, Butler BA, Mullen M, Bononi P. Effects of a behavioral weight loss program stressing calorie restriction versus calorie plus fat restriction in obese individuals with NIDDM or a family history of diabetes. *Diabetes Care* 1995;18:1241–8

81. Perri MG, Nezu AM, McKelvey WF, Shermer RL, Renjilian DA, Viegener BJ. Relapse prevention training and problem-solving therapy in the long-term management of obesity. *J Consult Clin Psychol* 2001;69:722–6

82. Richelsen B, Tonstad S, Rössner S, et al. Effect of orlistat on weight regain and cardiovascular risk factors following a very-low-energy diet in abdominally obese patients: a 3-year randomized, placebo-controlled study. *Diabetes Care* 2007;30:27–32

For peer review only

83. Cummings DE, Arterburn DE, Westbrook EO, et al. Gastric bypass surgery vs intensive lifestyle and medical intervention for type 2 diabetes: the CROSSROADS randomised controlled trial. *Diabetologia* 2016;59:945–53

84. Daubenmier J, Moran PJ, Kristeller J, et al. Effects of a mindfulness-based weight loss intervention in adults with obesity: a randomized clinical trial. *Obesity* 2016;24:794–804

85. Dixon JB, Schachter LM, O'Brien PE, Jones K, Grima M, Lambert G, et al. Surgical vs. conventional therapy for weight loss treatment of obstructive sleep apnea: a randomized controlled trial. JAMA 2012;308:1142–9

86. Dutton GR, Nackers LM, Dubyak PJ, et al. A randomized trial comparing weight loss treatment delivered in large versus small groups. *Int J Behav Nutr Phys Act* 2014;11:123

87. Latner JD, Ciao AC, Wendicke AU, Murakami JM, Durso LE. Community-based behavioural weight-loss treatment: long-term maintenance of weight loss, physiological, and psychological outcomes. *Behav Res Ther* 2013;51:451–9

88. Lowe MR, Butryn ML, Thomas JG, Coletta M. Meal replacements, reduced energy density eating, and weight loss maintenance in primary care patients: a randomized controlled trial. *Obesity* 2014;22:94–100

89. Christensen P, Frederiksen R, Bliddal H, et al. Comparison of three weight maintenance programs on cardiovascular risk, bone and vitamins in sedentary older adults. *Obesity* 2013;21:1982–90

90. Damschroder LJ, Lutes LD, Kirsh S, et al. Small-changes obesity treatment among veterans: 12-month outcomes. *Am J Prev Med* 2014;47:541–53

For peer review only - http://bmjopen.bmj.com/site/about/guidelines.xhtml

91. Eaton CB, Hartman SJ, Perzanowski E, et al. A randomized clinical trial of a tailored lifestyle intervention for obese, sedentary, primary care patients. *Ann Fam Med* 2016;14:311–19

92. Wolf AM, Conaway MR, Crowther JQ, et al. Improving Control with Activity and Nutrition (ICAN) Study. Translating lifestyle intervention to practice in obese patients with type 2 diabetes: Improving Control with Activity and Nutrition (ICAN) study. *Diabetes Care* 2004;27:1570–6

93. Mensinger JL, Calogero RM, Stranges S, Tylka TL. A weight-neutral versus weight-loss approach for health promotion in women with high BMI: a randomized-controlled trial. *Appetite* 2016;105:364–74

94. Nackers LM, Middleton KR, Dubyak PJ, Daniels MJ, Anton SD, Perri MG. Effects of prescribing 1,000 versus 1,500 kilocalories per day in the behavioral treatment of obesity: a randomized trial. *Obesity* 2013;21:2481–7

95. Nilsen V, Bakke PS, Gallefoss F. Effects of lifestyle intervention in persons at risk for type 2 diabetes mellitus – results from a randomised, controlled trial. *BMC Public Health* 2011;11:893

96. O'Neil PM, Miller-Kovach K, Tuerk PW, et al. Randomized controlled trial of a nationally available weight control program tailored for adults with type 2 diabetes. *Obesity* 2016;24:2269–77

97. Djuric Z, Mirasolo J, Kimbrough L, Brown DR, Heilbrun LK, Canar L, et al. A pilot trial of spirituality counseling for weight loss maintenance in African American breast cancer survivors. *J Natl Med Assoc* 2009;101:552–64

98. Reichard A, Saunders MD, Saunders RR, Donnelly JE, Lauer E, Sullivan DK, Ptomey L. A comparison of two weight management programs for adults with mobility impairments. *Disabil Health J* 2015;8:61–9

For peer review only - http://bmjopen.bmj.com/site/about/guidelines.xhtml

99. Liu P, Ross JS, Ioannidis JP, Dhruva SS, Vasiliou V, Wallach JD. Prevalence and significance of race and ethnicity subgroup analyses in Cochrane intervention reviews. *Clinical Trials* 2020;17(2):231-234

100. Sutcliffe K, Melendez-Torres GJ, Burchet, HED, Richardso, M, Rees R, Thomas J. The importance of service-users' perspectives: A systematic review of qualitative evidence reveals overlooked critical features of weight management programmes. *Health Expect* 2018;21: 563– 573

101. De Las Nueces, D, Hacker K, DiGirolamo, A, Hicks LS. A Systematic Review of Community-Based Participatory Research to Enhance Clinical Trials in Racial and Ethnic Minority Groups. *Health Serv Res* 2012;47:1363-1386

102. Ní Shé, É, Morton, S, Lambert, V, et al. Clarifying the mechanisms and resources that enable the reciprocal involvement of seldom heard groups in health and social care research: A collaborative rapid realist review process. *Health Expect* 2019; 22: 298– 306

103. Arnegard ME, Whitten LA, Hunter C, and Clayton JA. Sex as a Biological Variable: A 5-Year Progress Report and Call to Action. *J Women's Health*. 2020.858-864.

104. Wendler D, Kington R, Madans J, Van Wye G, Christ-Schmidt H, et al. Are racial and ethnic minorities less willing to participate in health research? *PLoS Med* 2006; 3(2): e19.

105. Trial Forge [Internet]. Aberdeen, Scotland: Trial Forge; 2020 [cited February 2021]. The INCLUDE ethnicity framework. Available from: https://www.trialforge.org/trial-forge-centre/include/

106. Travers J, Marsh S, Williams M, et al. External validity of randomised controlled trials in asthma: to whom do the results of the trials apply? *Thorax* 2007;62:219-223

For peer review only - http://bmjopen.bmj.com/site/about/guidelines.xhtml

107. Saunders CD, Byrne B, Guthrie RS, et al. External validity of randomized controlled trials of glycaemic control and vascular disease: how representative are participants? *Diabet Med* 2013;30:300– 308

108. Hoppe C, Kerr D. Minority underrepresentation in cardiovascular outcome trials for type 2 diabetes. *Lancet Diabetes Endocrinol* 2017;5(1):13

109. Trivedi RB, Humphreys K. Participant exclusion criteria in treatment research on neurological disorders: Are unrepresentative study samples problematic? *Contemp Clin Trials* 2015;44:20–25

110. Schmid AF, Groenwold RHH, van Deldena JJM, et al. Justification of exclusion criteria was underreported in a review of cardiovascular trials. *J Clin Epidemiol* 2014;67:635-644

111. He J, Morales DR, Guthrie B. Exclusion rates in randomized controlled trials of treatments for physical conditions: a systematic review. *Trials* 2020;21:228

112. Hayba, N, Partridge SR, Nour MM, Grech A, Allman Farinelli M. Effectiveness of lifestyle interventions for preventing harmful weight gain among young adults from lower socioeconomic status and ethnically diverse backgrounds: a systematic review. *Obes Rev* 2018;19:333– 346

113. Olstad DL, Ancilotto R, Teychenne M, et al. Can targeted policies reduce obesity and improve obesity-related behaviours in socioeconomically disadvantaged populations? A systematic review. *Obes Rev* 2017;18:791– 807

114. Bambra CL, Hillier FC, Cairns J-M, Kasim A, Moore HJ, Summerbell CD. How effective are interventions at reducing socioeconomic inequalities in obesity among children and adults? Two systematic reviews. *Public Health Res* 2015;3(1).

For peer review only - http://bmjopen.bmj.com/site/about/guidelines.xhtml

115. Witham MD, Anderson E, Carroll C, et al. Developing a roadmap to improve trial delivery for under-served groups: results from a UK multi-stakeholder process. *Trials* 2020;21:694

116. Moher D, Hopewell S, Schulz K F, Montori V, GÃ¸tzsche P C, Devereaux P J et al. CONSORT 2010 Explanation and Elaboration: updated guidelines for reporting parallel group randomised trials *BMJ* 2010; 340 :c869

For peer review only

Acknowledgements

We thank the members of the REBALANCE Project and Advisory Groups for their contributions to the REBALANCE project. We thank Shaun Treweek and Heidi Gardner, Health Services Research Unit, University of Aberdeen, for helpful discussions on trial generalisability and inclusion of under-served groups.

Competing interests

None.

Author Contributorship

CR, MA-M, MC, MI, AA contributed to conception and design, acquisition of data, or analysis and interpretation of data, and revised data critically for important intellectual content of this manuscript. All authors critically reviewed the manuscript and approved the final version submitted for publication. CR takes responsibility for the integrity of the data and the accuracy of the data analysis.

For peer review only - http://bmjopen.bmj.com/site/about/guidelines.xhtml

Funding

The REBALANCE study was funded by the National Institute for Health Research (NIHR) Health Technology Assessment Programme (Project number: 15/09/04). The views expressed are those of the author(s) and not necessarily those of the NHS, the NIHR or the Department of Health. The Health Services Research Unit is funded by the Chief Scientist Office of the Scottish Government Health and Social Care Directorates.

Transparency statement

CR affirms that the manuscript is an honest, accurate, and transparent account of the study being reported; that no important aspects of the study have been omitted; and that any discrepancies from the study as planned have been explained

Data sharing

Study data are all available from the NIHR journals library ((REBALANCE) REview of Behaviour And Lifestyle interventions for severe obesity: AN evidenCE synthesis (nihr.ac.uk) or can be obtained from the lead author.

For peer review only

Appendix 1 Literature search strategies

Literature searching was conducted in June 2016 and updated in April/May 2017. The following databases were searched without language restriction: MEDLINE, MEDLINE Epub Ahead of Print & MEDLINE In-Process, PsychINFO, Cumulative Index to Nursing and Allied Health Literature (CINAHL), Science Citation Index (SCI), Cochrane Central Register of Controlled Trials (CENTRAL), and ClincalTrials.gov. Ovid MEDLINE and EMBASE autoalerts have been in place since 2002 and results are screened regularly for long-term RCTs on obesity management in adults (≥ 1 year of follow-up). Copies of relevant reports are retained and the reviewers hand-searched these reports eligible studies. Therefore, search strategies for MEDLINE and EMBASE excluded the results of the autoalert search (using the Boolean operator NOT). A supplementary search of MEDLINE was undertaken to identify systematic reviews of severe or morbid obesity, and reference lists were scrutinised for additional studies.

RCTs for Review 1 (weight loss or weight maintenance programmes for adults with obesity and Review 5 (weight loss or weight maintenance programmes for adults with obesity compared with bariatric surgery)
MEDLINE and EMBASE
Ovid multifile search: http://shibboleth.ovid.com/
Database: Embase <1988 to 2017 Week 17>, Ovid MEDLINE(R) Epub Ahead of Print, In-Process & Other Non-Indexed Citations, Ovid MEDLINE(R) Daily and Ovid MEDLINE(R) <1946 to Present> 25th April 2017
Date of Search: 25th April 2017
1 Obesity, Morbid/dh, dt, su, th use ppez
2 Morbid Obesity/dt, su, th use emed
3 Obesity, Morbid/ use ppez
4 Morbid Obesity/ use emed
5 (obes$ adj3 (morbid$ or severe$)).tw.
6 (morbid obesity or severe obesity).kw.
7 (bmi adj ("27$" or "28$" or "29$")).tw.
8 (bmi adj ("30$" or "31$" or "32$" or "33$" or "34$" or "35$" or "36$" or "37$" or "38$" or "39$")).tw.
9 (bmi adj ("40$" or "41$" or "42$" or "43$" or "44$" or "45$" or "46$" or "47$" or "48$" or "49$")).tw.
10 (bmi adj ("50$" or "51$" or "52$" or "53$" or "54$" or "55$")).tw.
11 (body mass index adj ("35$" or "36$" or "37$" or "38$" or "39$")).tw.
12 (body mass index adj ("40$" or "41$" or "42$" or "43$" or "44$" or "45$" or "46$" or "47$" or "48$" or "49$")).tw.
13 (body mass index adj ("50$" or "51$" or "52$" or "53$" or "54$" or "55$")).tw.
14 or/3-12
15 exp bariatric surgery/
16 bariatric.tw,kw.

For peer review only

17 (gastric adj3 (bypass or bypass or band$ or balloon$)).tw.
18 (gastroplast$ or gastrect$).tw.
19 (jejunoileal adj3 (bypass or by pass)).tw.
20 lipectom$.tw
21 diet therapy/ use ppez or caloric restriction/ use ppez or diet, carbohydrate-restricted/ use ppez or diet, fat-restricted/ use ppez or diet, reducing/ use ppez
22 diet/ use emed or low calory diet/ use emed or low carbohydrate diet/ use emed
23 (diet adj3 (restrict$ or reduc$ or modif$ or calorie$)).tw.
24 (calori$ adj3 (reduc$ or restrict$ or limit$)).tw.
25 exp anti-obesity agents/ use ppez
26 tetrahydrolipstatin/ use emed
27 sibutramine/ use emed
28 rimonabant/ use emed
29 (appetite adj3 (reduc$ or depress$)).tw.
30 (orlistat or xenical).tw,rn.
31 (sibutramin$ or arcalion or reductil or medaria or meridia).tw,rn.
32 (rimonabant or acomplia or zimulti).tw,rn.
33 exp exercise/
34 exp sports/
35 (exercise or aerobic or physical activ$).tw.
36 weight loss/ use ppez
37 weight reduction/ use emed
38 (weight adj3 (reduc$ or loss or lose)).tw.
39 Weight Reduction Programs/ use ppez
40 exp Body Weight Management/ use emed
41 Behavior Therapy/
42 Health Education/
43 Counseling/ use ppez
44 Nutritional counseling/ use emed
45 Life Style/ use ppez
46 Lifestyle modification/ use emed
47 (modif$ adj3 (behavio?r or diet or eat? or eating)).tw.
48 ((diet or weight) adj3 (educat$ or counsel$)).tw.

For peer review only

49 or/15-46
50 1 or 2 or (14 and 49)
51 randomized controlled trial.pt.
52 controlled clinical trial.pt
53 randomi?ed.ab.
54 placebo.ab.
55 drug therapy.fs.
56 randomly.ab.
57 trial.ab.
58 groups.ab
59 nonhuman/ not human/
60 exp clinical trial/ use emed
61 randomization/ use emed
62 51 or 52 or 53 or 54 or 55 or 56 or 57 or 58 or 60 or 61
63 exp animals/ use ppez not humans/ use ppez
64 nonhuman/ use emed or animal/ use emed
65 64 not exp human/ use emed
66 62 not (63 or 65)
67 50 and 66
68 67 not (abstract or letter or note or editorial or comment).pt.
69 limit 68 to yr="1990 -Current"
70 obesity/
71 (obesity adj2 (morbid or diabet$)).tw.
72 obesity, morbid/ use ppez
73 morbid obesity/ use emed
74 obes$.tw.
75 weight loss/ use ppez
76 weight reduction/ use emed
77 (weight adj1 (los$ or reduc$ or maint$ or control)).tw.
78 (diet adj5 weight).tw.
79 overweight.tw.
80 or/70-79
81 80 and 66

For peer review only

82 69 not 81

PsycINFO

Ovid: http://shibboleth.ovid.com/

Database: PsycINFO <1987 to April Week 3 2017>

Date of Search: 25th April 2017

1 (obes$ adj3 (morbid$ or severe$)).tw.
2 (bmi adj ("27$" or "28$" or "29$")).tw.
3 (bmi adj ("30$" or "31$" or "32$" or "33$" or "34$" or "35$" or "36$" or "37$" or "38$" or "39$")).tw.
4 (bmi adj ("40$" or "41$" or "42$" or "43$" or "44$" or "45$" or "46$" or "47$" or "48$" or "49$")).tw.
5 (bmi adj ("50$" or "51$" or "52$" or "53$" or "54$" or "55$")).tw. (
6 (body mass index adj ("27$" or "28$" or "29$")).tw.
7 (body mass index adj ("30$" or "31$" or "32$" or "33$" or "34$" or "35$" or "36$" or "37$" or "38$" or "39$")).tw.
8 (body mass index adj ("40$" or "41$" or "42$" or "43$" or "44$" or "45$" or "46$" or "47$" or "48$" or "49$")).tw.
9 (body mass index adj ("50$" or "51$" or "52$" or "53$" or "54$" or "55$")).tw. (7)
10 or/1-9
11 clinical trials/
12 randomi?ed.tw,md.
13 randomly.tw,md.
14 trial?.tw,md
15 or/11-14
16 10 and 15

CINAHL

http://search.ebscohost.com

!990- 25th April 2017

For peer review only

Date of Search: 25th April 2017
S1 (MH "Obesity, Morbid") OR TX (obes|* N3 (morbid or sever*))
S2 TX (bmi N3 (27 or 28 or 29 or 30 or 31 or 32 or 33 or 34 or 35 or 36 or 37 or 38 or 39).) OR TX (bmi N3 (40 or 41 or 42 or 43 or 44 or 45 or 46 or 47 or 48 or 49).) OR TX (bmi N3 (50 or 51 or 52 or 53 or 54 or 55).)
S3 TX (body mass index N3 (35 or 36 or 37 or 38 or 39).) OR TX (body mass index N3 (40 or 41 or 42 or 43 or 44 or 45 or 46 or 47 or 48 or 49).) OR TX (body mass index N3 (50 or 51 or 52 or 53 or 54 or 55).)
S4 S1 OR S2 OR S3
S5 (MH "Bariatric Surgery+")
S6 TX bariatric OR TX gastroplasty
S7 TX bariatric OR TX gastroplast* AND TX gastrect*
S8 TX (gastric N3 (by pass or by pass or band* or balloon*)) OR TX (jejunoileal N3 (bypass or by pass) OR TX lipectom*
S9 (MH "Diet Therapy") OR (MH "Diet, Reducing") OR (MH "Restricted Diet") OR (MH "Diet, Fat-Restricted") OR (MH "Diet, Low Carbohydrate")
S10 TX (diet N3 (restrict* or reduc* or modif* or calorie*))) OR TX (calori* N3 (reduc* or restrict* or limit*)).)
S11 (MH "Antiobesity Agents+")
S12 TX (orlistat or xenical) OR TX (sibutramin$ or arcalion or reductil or medaria or meridia) OR TX (rimonabant or acomplia or zimulti)
S13 (MH "Exercise+")
S14 TX exercise or aerobic or physical activ*).
S15 (MH "Physical Fitness")
S16 (MH "Weight Loss")
S17 TX weight N3 (reduc* or loss or lose)).
S18 (MH "Weight Reduction Programs")
S19 (MH "Counseling")
S20 (MH "Life Style") OR (MH "Life Style Changes")
S21 TX (modif* N3 (behavior or behaviour or diet or eat* or eating) OR TX ((diet or weight) N3 (educat* or counsel*)).)

For peer review only

S22 S5 OR S6 OR S7 OR S8 OR S9 OR S10 OR S11 OR S12 OR S13 OR S14 OR S15 OR S16 OR S17 OR S18 OR S19 OR S21
S23 S4 AND S22 Limiters - Published Date: 19900101-20161231
S24 S4 AND S22 Limiters - Exclude MEDLINE records

Science Citation Index
www.webofknowledge.com
1988 - 25th April 2017
Date of Search: 25th April 2017

1 (TS=(morbid NEAR/3 obes$)) AND DOCUMENT TYPES: (Article)
2 (TS=(severe$ NEAR/3 obes$)) AND DOCUMENT TYPES: (Article)
3 (TS=(bmi NEAR/3 (27 or 28 or 29 or 30 or 31 or 32 or 33 or 34 or 35 or 36 or 37 or 38 or 39))) AND DOCUMENT TYPES: (Article)
4 ((TS=(bmi NEAR/3 (40 or 41 or 42 or 43 or 44 or 45 or 46 or 47 or 48 or 49)))) AND DOCUMENT TYPES: (Article)
5 ((TS=(bmi NEAR/3 (50 or 51 or 52 or 53 or 54 or 55)))) AND DOCUMENT TYPES: (Article)
6 #5 OR #4 OR #3 OR #2 OR #1
7 (TS=(randomized or randomised or randomly)) AND DOCUMENT TYPES: (Article)
8 (TS=clinical trial*) AND DOCUMENT TYPES: (Article)
9 #8 OR #7
10 #9 AND #6
11 (TS=(bariatric or gastroplast* or gastrect*)) AND DOCUMENT TYPES: (Article)
12 (TS=((gastric or jejunoileal) NEAR/3 bypass)) AND DOCUMENT TYPES: (Article)
13 ((TS=((gastric or jejunoileal) NEAR/3 "by pass"))) AND DOCUMENT TYPES: (Article)
14 (TS=(gastric NEAR/3 (band* or balloon*))) AND DOCUMENT TYPES: (Article)

For peer review only

15 (TS=(diet NEAR/3 (restrict* or reduc* or modif* or calorie*)).) AND DOCUMENT TYPES: (Article)
16 (TS=(calori* NEAR/3 (reduc* or restrict* or limit*)).) AND DOCUMENT TYPES: (Article)
17 (TS=(appetite NEAR/3 (reduc* or depress*)).) AND DOCUMENT TYPES: (Article)
18 (TS=(orlistat or xenical).) AND DOCUMENT TYPES: (Article)
19 (TS=(sibutramin or arcalion or reductil or medaria or meridia).) AND DOCUMENT TYPES: (Article)
20 (TS=(rimonabant or acomplia or zimulti).) AND DOCUMENT TYPES: (Article)
21 (TS=(exercise or aerobic or physical active*).) AND DOCUMENT TYPES: (Article)
22 (TS=(weight NEAR/3 (reduc* or loss or lose))) AND DOCUMENT TYPES: (Article)
23 (TS=(modif* NEAR/3 (behavior or behaviour or diet or eat? or eating)).) AND DOCUMENT TYPES: (Article)
24 (TS=(diet NEAR/3 (educat* or counsel*)).) AND DOCUMENT TYPES: (Article)
25 (TS=(weight NEAR/3 (educat* or counsel*))) AND DOCUMENT TYPES: (Article)
26 #25 OR #24 OR #23 OR #22 OR #21 OR #20 OR #19 OR #18 OR #17 OR #16 OR #15 OR #14 OR #13 OR #12 OR #11
#27 #10 AND #26

Cochrane Central Register of Controlled Trials

www.thecochranelibrary.com
Issue 4 April 2017

Date of Search: 25th April 2017

#1 MeSH descriptor: [Obesity, Morbid] explode all trees and with qualifier(s): [Diet therapy - DH, Surgery - SU, Therapy - TH]

For peer review only

#2 MeSH descriptor: [Obesity, Morbid] explode all trees
#3 obes* near/3 (morbid* or severe*):ti,ab,kw (Word variations have been searched)
#4 bmi near/1 (27* or 28* or 29* or 30* or 31* or 32* or 33* or 34* or 35* or 36* or 37* or 38* or 39*):ti,ab,kw or bmi near/1 (40* or 41* or 42* or 43* or 44* or 45* or 46* or 47* or 48* or 49*):ti,ab,kw or bmi near/1 (50* or 51* or 52* or 53* or 54* or 55*):ti,ab,kw (Word variations have been searched)
#5 body mass index near/1 (27* or 28* or 29* or 30* or 31* or 32* or 33* or 34* or 35* or 36* or 37* or 38* or 39*):ti,ab,kw or body mass index near/1 (40* or 41* or 42* or 43* or 44* or 45* or 46* or 47* or 48* or 49*):ti,ab,kw or body mass index near/1 (50* or 51* or 52* or 53* or 54* or 55*):ti,ab,kw (Word variations have been searched)
#6 #2 or #3 or #4 or #5
#7 MeSH descriptor: [Bariatric Surgery] explode all trees
#8 bariatric:ti,ab,kw (Word variations have been searched)
#9 gastric near/3 (bypass or by pass or band* or balloon*):ti,ab,kw or gastroplast* or gastrect*:ti,ab,kw or lipectom*:ti,ab,kw or jejunoileal near/3 (bypass or by pass):ti,ab,kw (Word variations have been searched)
#10 MeSH descriptor: [Diet Therapy] explode all trees
#11 MeSH descriptor: [Diet, Reducing] explode all trees
#12 MeSH descriptor: [Diet, Fat-Restricted] explode all trees
#13 MeSH descriptor: [Diet, Carbohydrate-Restricted] explode all trees
#14 MeSH descriptor: [Caloric Restriction] explode all trees
#15 diet near/3 (restrict* or reduc* or modif* or calorie*):ti,ab,kw or calori* near/3 (reduc* or restrict* or limit*):ti,ab,kw (Word variations have been searched)
#16 MeSH descriptor: [Anti-Obesity Agents] explode all trees
#17 appetite near/3 (reduc* or depress*):ti,ab,kw or orlistat or xenical:ti,ab,kw or sibutramin or arcalion or reductil or medaria or meridia:ti,ab,kw or rimonabant or acomplia or zimulti:ti,ab,kw (Word variations have been searched)
#18 MeSH descriptor: [Exercise] explode all trees
#19 MeSH descriptor: [Sports] explode all trees
#20 exercise or aerobic* or physical activ*:ti,ab,kw (Word variations have been searched)

For peer review only

#21 MeSH descriptor: [Weight Loss] explode all trees
#22 MeSH descriptor: [Weight Reduction Programs] explode all trees
#23 weight near/3 (reduc* or loss or lose) .:ti,ab,kw (Word variations have been searched)
#24 weight near/3 (reduc* or loss or lose) .:ti,ab,kw (Word variations have been searched)
#25 MeSH descriptor: [Behavior Therapy] explode all trees
#26 MeSH descriptor: [Health Education] explode all trees
#27 MeSH descriptor: [Counseling] explode all trees
#28 MeSH descriptor: [Life Style] explode all trees
#29 diet near/3 (educat* or counsel*):ti,ab,kw or modif* near/3 (behaviour or behavior or diet or eat or eating):ti,ab,kw or weight near/3 (educat* or counsel*):ti,ab,kw (Word variations have been searched)
#30 #7 or #8 or #9 or #10 or #11 or #12 or #13 or #14 or #15 or #16 or #17 or #18 or #19 or #20 or #21 or #22 or #23 or #24 or #25 or #26 or #27 or #28 or #29
#31 #6 and #30
#32 #1 or #31 Publication Year from 1990 to 2016, in Trials
#33 conference abstract (Word variations have been searched)
#34 #32 not #33

For peer review only

Appendix 2 PRISMA flow diagram

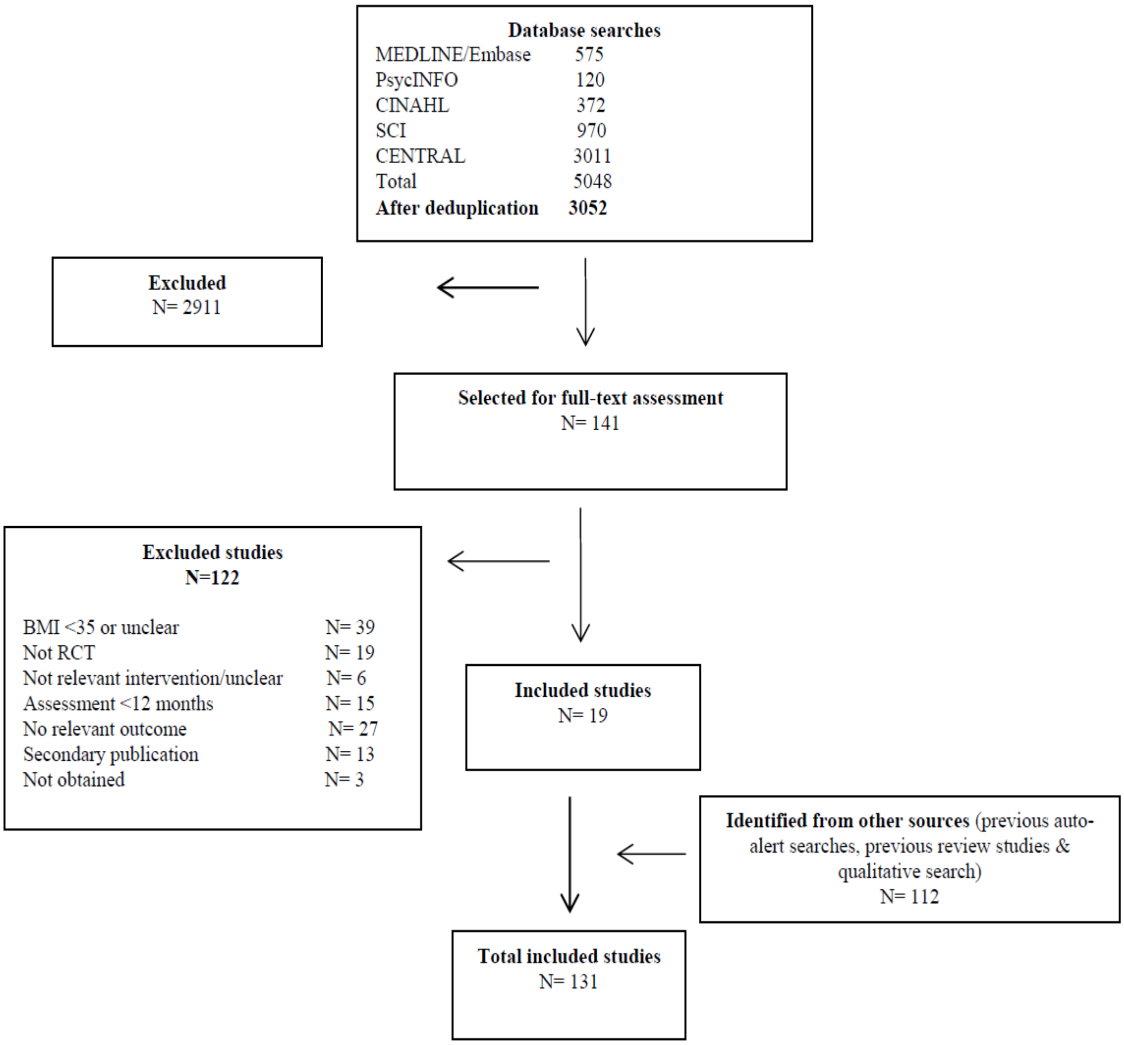

Appendix 3 List of included studies

Agras WS, Berkowitz RI, Arnow BA, Telch CF, Marnell M, Henderson J, et al. Maintenance following a very-low-calorie diet. *Journal of Consulting & Clinical Psychology* 1996;**64:**610-3.

Annesi JJ. Mediation of the relationship of behavioural treatment type and changes in psychological predictors of healthy eating by body satisfaction changes in women with obesity. *Obesity Research & Clinical Practice* 2017;**11:**97-107.

Appel LJ, Clark JM, Yeh HC, Wang NY, Coughlin JW, Daumit G, et al. Comparative effectiveness of weight-loss interventions in clinical practice. *New England Journal of Medicine* 2011;**365:**1959-68.

Azar KM, Xiao L, Ma J. Baseline obesity status modifies effectiveness of adapted diabetes prevention program lifestyle interventions for weight management in primary care. *BioMed Research International* 2013;191209.

Bacon L, Keim NL, Van Loan MD, Derricote M, Gale B, Kazaks A, et al. Evaluating a 'non-diet' wellness intervention for improvement of metabolic fitness, psychological well-being and eating and activity behaviors. *International Journal of Obesity & Related Metabolic Disorders* 2002;**26:**854-65.

Bakris G, Calhoun D, Egan B, Hellmann C, Dolker M, Kingma I. Orlistat improves blood pressure control in obese subjects with treated but inadequately controlled hypertension. *Journal of Hypertension* 2002;**20:**2257-67.

Bartels SJ, Pratt SI, Aschbrenner KA, Barre LK, Jue K, Wolfe RS, et al. Clinically Significant Improved Fitness and Weight Loss Among Overweight Persons With Serious Mental Illness. *Psychiatric Services* 2013;**64:**729-36.

Bartels SJ, Pratt SI, Aschbrenner KA, Barre LK, Naslund JA, Wolfe R, et al. Pragmatic Replication Trial of Health Promotion Coaching for Obesity in Serious Mental Illness and Maintenance of Outcomes. *American Journal of Psychiatry* 2015;**172:**344-52.

Bazzano LA, Hu T, Reynolds K, Yao L, Bunol C, Liu Y, et al. Effects of low-carbohydrate and low-fat diets: a randomized trial. *Annals of Internal Medicine* 2014;**161:**309-18.

Bennett GG, Warner ET, Glasgow RE, Askew S, Goldman J, Ritzwoller DP, et al. Obesity treatment for socioeconomically disadvantaged patients in primary care practice. *Archives of Internal Medicine* 2012;**172:**565-74.

Berry DC, Schwartz TA, McMurray RG, Skelly AH, Neal M, Hall EG, et al. The family partners for health study: a cluster randomized controlled trial for child and parent weight management. *Nutrition & Diabetes* 2014;**4:**e101.

For peer review only

Berteus Forslund H, Klingstrom S, Hagberg H, Londahl M, Torgerson JS, Lindroos AK. Should snacks be recommended in obesity treatment? A 1-year randomized clinical trial. *European Journal of Clinical Nutrition* 2008;**62:**1308-17.

Beutel ME, Dippel A, Szczepanski M, Thiede R, Wiltink J. Mid-term effectiveness of behavioral and psychodynamic inpatient treatments of severe obesity based on a randomized study. *Psychotherapy and Psychosomatics* 2006;**75:**337-45.

Bliddal H, Leeds AR, Stigsgaard L, Astrup A, Christensen R. Weight loss as treatment for knee osteoarthritis symptoms in obese patients: 1-Year results from a randomised controlled trial. *Annals of the Rheumatic Diseases* 2011;**70:**1798-803.

Brehm BJ, Lattin BL, Summer SS, Boback JA, Gilchrist GM, Jandacek RJ, et al. One-year comparison of a high-monounsaturated fat diet with a high-carbohydrate diet in type 2 diabetes. *Diabetes Care* 2009;**32:**215-20.

Broom I, Hughes E, Dodson P, Reckless J. The role of orlistat in the treatment of obese patients with mild to moderate hypercholesterolaemia: Consequences for coronary risk. *British Journal of Cardiology* 2002;**9:**460-1+8.

Broom I, Wilding J, Stott P, Myers N. Randomised trial of the effect of orlistat on body weight and cardiovascular disease risk profile in obese patiensts: UK multimorbidity study. *International Journal of Clinical Practice* 2002;**56:**494-9.

Burguera B, Jesus Tur J, Escudero AJ, Alos M, Pagan A, Cortes B, et al. An Intensive Lifestyle Intervention Is an Effective Treatment of Morbid Obesity: The TRAMOMTANA Study-A Two-Year Randomized Controlled Clinical Trial. *International journal of endocrinology* 2015.

Cheskin LJ, Mitchell AM, Jhaveri AD, Mitola AH, Davis LM, Lewis RA, et al. Efficacy of meal replacements versus a standard food-based diet for weight loss in type 2 diabetes: a controlled clinical trial. *Diabetes Educator* 2008;**34:**118-27.

Christensen P, Frederiksen R, Bliddal H, Riecke BF, Bartels EM, Henriksen M, et al. Comparison of three weight maintenance programs on cardiovascular risk, bone and vitamins in sedentary older adults. *Obesity* 2013;**21:**1982-90.

Courcoulas AP, Goodpaster BH, Eagleton JK, Belle SH, Kalarchian MA, Lang W, et al. Surgical vs medical treatments for type 2 diabetes mellitus: a randomized clinical trial. *JAMA Surgery* 2014;**149:**707-15.

Cummings DE, Arterburn DE, Westbrook EO, Kuzma JN, Stewart SD, Chan CP, et al. Gastric bypass surgery vs intensive lifestyle and medical intervention for type 2 diabetes: the CROSSROADS randomised controlled trial. *Diabetologia* 2016;**59:**945-53.

For peer review only

Dalle Grave R, Calugi S, Gavasso I, El Ghoch M, Marchesini G. A randomized trial of energy-restricted high-protein versus high-carbohydrate, low-fat diet in morbid obesity. *Obesity* 2013;**21:**1774-81.

Damschroder LJ, Lutes LD, Kirsh S, Kim HM, Gillon L, Holleman RG, et al. Small-changes obesity treatment among veterans: 12-month outcomes. *American Journal of Preventive Medicine* 2014;**47:**541-53.

Daubenmier J, Moran PJ, Kristeller J, Acree M, Bacchetti P, Kemeny ME, et al. Effects of a mndfulness-based weight loss intervention in adults with obesity: A randomized clinical trial. *Obesity* 2016;**24:**794-804.

Daumit GL, Dickerson FB, Wang NY, Dalcin A, Jerome GJ, Anderson CAM, et al. A behavioral weight-loss intervention in persons with serious mental illness. *New England Journal of Medicine* 2013;**368:**1594-602.

Davidson MH, Hauptman J, DiGirolamo M, Foreyt JP, Halsted CH, Heber D, et al. Weight control and risk factor reduction in obese subjects treated for 2 years with orlistat: A randomized controlled trial. *Journal of the American Medical Association* 1999;**281:**235-42.

Davis NJ, Tomuta N, Schechter C, Isasi CR, Segal-Isaacson CJ, Stein D, et al. Comparative study of the effects of a 1-year dietary intervention of a low-carbohydrate diet versus a low-fat diet on weight and glycemic control in type 2 diabetes. *Diabetes Care* 2009;**32:**1147-52.

Delbridge EA, Prendergast LA, Pritchard JE, Proietto J. One-year weight maintenance after significant weight loss in healthy overweight and obese subjects: Does diet composition matter? *American Journal of Clinical Nutrition* 2009;**90:**1203-14.

Dennison KF, Galante D, Dennison D, Golaszewski T. A one year post-program assessment of a computer-assisted instruction weight management program for industrial employees: lessons learned. *Journal of Health Education* 1996;**27:**38-43.

Dixon JB, O'Brien PE, Playfair J, Chapman L, Schachter LM, Skinner S, et al. Adjustable gastric banding and conventional therapy for type 2 diabetes: a randomized controlled trial. *JAMA* 2008;**299:**316-23.

Dixon JB, Schachter LM, O'Brien PE, Jones K, Grima M, Lambert G, et al. Surgical vs conventional therapy for weight loss treatment of obstructive sleep apnea: A randomized controlled trial. *JAMA* 2012;**308:**1142-9.

Djuric Z, Mirasolo J, Kimbrough L, Brown DR, Heilbrun LK, Canar L, et al. A pilot trial of spirituality counseling for weight loss maintenance in African American breast cancer survivors. *Journal of the National Medical Association* 2009;**101:**552-64.

For peer review only

Dutton GR, Nackers LM, Dubyak PJ, Rushing NC, Huynh TVT, Tan F, et al. A randomized trial comparing weight loss treatment delivered in large versus small groups. *International Journal of Behavioral Nutrition and Physical Activity* 2014;**11 (1)**

Eaton CB, Hartman SJ, Perzanowski E, Pan GH, Roberts MB, Risica PM, et al. A Randomized Clinical Trial of a Tailored Lifestyle Intervention for Obese, Sedentary, Primary Care Patients. *Annals of Family Medicine* 2016;**14:**311-9.

Ebbeling CB, Leidig MM, Feldman HA, Lovesky MM, Ludwig DS. Effects of a low-glycemic load vs low-fat diet in obese young adults: A randomized trial. *Journal of the American Medical Association* 2007;**297:**2092-102.

Esposito K, Giugliano F, Di Palo C, Giugliano G, Marfella R, D'Andrea F, et al. Effect of lifestyle changes on erectile dysfunction in obese men: A randomized controlled trial. *Journal of the American Medical Association* 2004;**291:**2978-84.

Finer N, James WPT, Kopelman PG, Lean MEJ, Williams G. One-year treatment of obesity: A randomized, double-blind, placebo-controlled, multicentre study of orlistat, a gastrointestinal lipase inhibitor. *International Journal of Obesity* 2000;**24:**306-13.

Fitzgibbon ML, Stolley MR, Schiffer L, Sharp LK, Singh V, Dyer A. Obesity reduction black intervention trial (ORBIT): 18-month results. *Obesity* 2010;**18:**2317-25.

Flechtner-Mors M, Boehm BO, Wittmann R, Thoma U, Ditschuneit HH. Enhanced weight loss with protein-enriched meal replacements in subjects with the metabolic syndrome. *Diabetes/Metabolism Research and Reviews* 2010;**26:**393-405.

Foster GD, Wyatt HR, Hill JO, Makris AP, Rosenbaum DL, Brill C, et al. Weight and metabolic outcomes after 2 years on a low-carbohydrate versus low-fat diet: A randomized trial. *Annals of Internal Medicine* 2010;**153:**147-57.

Goodpaster BH, Delany JP, Otto AD, Kuller L, Vockley J, South-Paul JE, et al. Effects of diet and physical activity interventions on weight loss and cardiometabolic risk factors in severely obese adults: a randomized trial. *JAMA* 2010;**304:**1795-802.

Gorin AA, Raynor HA, Fava J, Maguire K, Robichaud E, Trautvetter J, et al. Randomized controlled trial of a comprehensive home environment-focused weight-loss program for adults. *Health Psychology* 2013;**32:**128-37.

Green CA, Yarborough BJH, Leo MC, Stumbo SP, Perrin NA, Nichols GA, et al. Weight maintenance following the STRIDE lifestyle intervention for individuals taking antipsychotic medications. *Obesity* 2015;**23:**1995-2001.

Hakala P, Karvetti RL, Rönnemaa T. Group vs. individual weight reduction programmes in the treatment of severe obesity--a five year follow-up study. *International journal of obesity and related metabolic disorders* 1993;**17:**97-102.
Hakala P. Weight reduction programmes at a rehabilitation centre and a health centre based on group counselling and individual support: Short- and long-term follow-up study. *International Journal of Obesity* 1994;**18:**483-9.

For peer review only

Hauptman J, Lucas C, Boldrin MN, Collins H, Segal KR. Orlistat in the long-term treatment of obesity in primary care settings. *Archives of Family Medicine* 2000;**9:**160-7.

Hunt K, Wyke S, Gray CM, Anderson AS, Brady A, Bunn C, et al. A gender-sensitised weight loss and healthy living programme for overweight and obese men delivered by Scottish Premier League football clubs (FFIT): A pragmatic randomised controlled trial. *The Lancet* 2014;**383:**1211-21.

Iqbal N, Vetter ML, Moore RH, Chittams JL, Dalton-Bakes CV, Dowd M, et al. Effects of a low-intensity intervention that prescribed a low-carbohydrate vs. a low-fat diet in obese, diabetic participants. *Obesity* 2010;**18:**1733-8.

Jesudason DR, Pedersen E, Clifton PM. Weight-loss diets in people with type 2 diabetes and renal disease: a randomized controlled trial of the effect of different dietary protein amounts. *American Journal of Clinical Nutrition* 2013;**98:**494-501.

Kahleova H, Hill M, Pelikanova T. Vegetarian vs. conventional diabetic diet - A 1-year follow-up. *Cor et Vasa* 2014;**56:**e140-e4.

Kelley DE, Bray GA, Pi-Sunyer FX, Klein S, Hill J, Miles J, et al. Clinical efficacy of orlistat therapy in overweight and obese patients with insulin-treated type 2 diabetes: A 1-year randomized controlled trial. *Diabetes Care* 2002;**25:**1033-41.

Keranen AM, Savolainen MJ, Reponen AH, Kujari ML, Lindeman SM, Bloigu RS, et al. The effect of eating behavior on weight loss and maintenance during a lifestyle intervention. *Preventive Medicine* 2009;**49:**32-8.

Khoo J, Piantadosi C, Duncan R, Worthley SG, Jenkins A, Noakes M, et al. Comparing effects of a low-energy diet and a high-protein low-fat diet on sexual and endothelial function, urinary tract symptoms, and inflammation in obese diabetic men. *Journal of Sexual Medicine* 2011;**8:**2868-75.

Krebs JD, Elley CR, Parry-Strong A, Lunt H, Drury PL, Bell DA, et al. The Diabetes Excess Weight Loss (DEWL) Trial: A randomised controlled trial of high-protein versus high-carbohydrate diets over 2 years in type 2 diabetes. *Diabetologia* 2012;**55:**905-14.

Krempf M, Louvet JP, Allanic H, Miloradovich T, Joubert JM, Attali JR. Weight reduction and long-term maintenance after 18 months treatment with orlistat for obesity. *International Journal of Obesity* 2003;**27:**591-7.

For peer review only

Kumanyika SK, Fassbender JE, Sarwer DB, Phipps E, Allison KC, Localio R, et al. One-year results of the think health study of weight management in primary care practices. *Obesity* 2012;**20:**1249-57.

Kumanyika SK, Shults J, Fassbender J, Whitt MC, Brake V, Kallan MJ, et al. Outpatient weight management in African-Americans: The Healthy Eating and Lifestyle Program (HELP) study. *Preventive Medicine* 2005;**41:**488-502.

Kumanyika SK, Wadden TA, Shults J, Fassbender JE, Brown SD, Bowman MA, et al. Trial of family and friend support for weight loss in African American adults. *Archives of Internal Medicine* 2009;**169:**1795-804.

Lantz H, Peltonen M, Agren L, Torgerson JS. Intermittent versus on-demand use of a very low calorie diet: A randomized 2-year clinical trial. *Journal of Internal Medicine* 2003;**253:**463-71.

Latner JD, Ciao AC, Wendicke AU, Murakami JM, Durso LE. Community-based behavioral weight-loss treatment: Long-term maintenance of weight loss, physiological, and psychological outcomes. *Behaviour Research and Therapy* 2013;**51:**451-9.

Lillis J, Niemeier HM, Thomas JG, Unick J, Ross KM, Leahey TM, et al. A Randomized Trial of an Acceptance-Based Behavioral Intervention for Weight Loss in People with High Internal Disinhibition. *Obesity* 2016;**24:**2509-14.

Linde JA, Simon GE, Ludman EJ, Ichikawa LE, Operskalski BH, Arterburn D, et al. A randomized controlled trial of behavioral weight loss treatment versus combined weight loss/depression treatment among women with comorbid obesity and depression. *Annals of behavioral medicine* 2011;**41:**119-30.

Little P, Stuart B, Hobbs FR, Kelly J, Smith ER, Bradbury KJ, et al. An internet-based intervention with brief nurse support to manage obesity in primary care (POWeR+): a pragmatic, parallel-group, randomised controlled trial. *The Lancet Diabetes and Endocrinology* 2016;**4:**821-8.

Look Ahead Research Group, Wing RR, Bolin P, Brancati FL, Bray GA, Clark JM, et al. Cardiovascular effects of intensive lifestyle intervention in type 2 diabetes.[Erratum appears in N Engl J Med. 2014 May 8;370(19):1866]. *New England Journal of Medicine* 2013;**369:**145-54.

Lowe MR, Butryn ML, Thomas JG, Coletta M. Meal replacements, reduced energy density eating, and weight loss maintenance in primary care patients: a randomized controlled trial. *Obesity* 2014;**22:**94-100.

Ma J, Strub P, Xiao L, Lavori PW, Camargo CA, Jr., Wilson SR, et al. Behavioral weight loss and physical activity intervention in obese adults with asthma. A randomized trial. *Annals of the American Thoracic Society* 2015;**12:**1-11.
Maclaughlin HL, Hall WL, Patel AG, Blacklock RM, Swift PA, Phanish MK, et al. Weight loss, adipokines, and quality of life after sleeve gastrectomy in obese patients with stages 3-4 CKD: A randomized controlled pilot study. *American Journal of Kidney Diseases* 2014;**64:**660-3.

For peer review only - http://bmjopen.bmj.com/site/about/guidelines.xhtml

Manzoni GM, Cesa GL, Bacchetta M, Castelnuovo G, Conti S, Gaggioli A, et al. Virtual reality-enhanced cognitive-behavioral therapy for morbid obesity: A randomized controlled study with 1 year follow-up. *Cyberpsychology, Behavior, and Social Networking* 2016;**19:**134-40.

Martin PD, Dutton GR, Rhode PC, Horswell RL, Ryan DH, Brantley PJ. Weight loss maintenance following a primary care intervention for low-income minority women. *Obesity* 2008;**16:**2462-7.

Mayer-Davis EJ, D'Antonio AM, Smith SM, Kirkner G, Martin SL, Parra-Medina D, et al. Pounds off with empowerment (POWER): A clinical trial of weight management strategies for black and white adults with diabetes who live in medically underserved rural communities. *American Journal of Public Health* 2004;**94:**1736-42.

McRobbie H, Hajek P, Peerbux S, Kahan BC, Eldridge S, Trepel D, et al. Tackling obesity in areas of high social deprivation: clinical effectiveness and cost-effectiveness of a task-based weight management group programme - a randomised controlled trial and economic evaluation. *Health Technology Assessment* 2016;**20(79).**.

Melin I, Karlstrom B, Lappalainen R, Berglund L, Mohsen R, Vessby B. A programme of behaviour modification and nutrition counselling in the treatment of obesity: A randomised 2-y clinical trial. *International Journal of Obesity* 2003;**27:**1127-35.

Mensinger JL, Calogero RM, Stranges S, Tylka TL. A weight-neutral versus weight-loss approach for health promotion in women with high BMI: A randomized-controlled trial. *Appetite* 2016;**105:**364-74.

Miles JM, Leiter L, Hollander P, Wadden T, Anderson JW, Doyle M, et al. Effect of orlistat in overweight and obese patients with type 2 diabetes treated with metformin. *Diabetes Care* 2002;**25:**1123-8.

Mingrone G, Greco AV, Giancaterini A, Scarfone A, Castagneto M, Pugeat M. Sex hormone-binding globulin levels and cardiovascular risk factors in morbidly obese subjects before and after weight reduction induced by diet or malabsorptive surgery. *Atherosclerosis* 2002;**161:**455-62.

Mingrone G, Panunzi S, De Gaetano A, Guidone C, Iaconelli A, Leccesi L, et al. Bariatric surgery versus conventional medical therapy for type 2 diabetes. *New England Journal of Medicine* 2012;**366:**1577-85.

For peer review only

Moreno B, Bellido D, Sajoux I, Goday A, Saavedra D, Crujeiras AB, et al. Comparison of a very low-calorie-ketogenic diet with a standard low-calorie diet in the treatment of obesity. *Endocrine* 2014;**47:**793-805.

Nackers LM, Middleton KR, Dubyak PJ, Daniels MJ, Anton SD, Perri MG. Effects of prescribing 1,000 versus 1,500 kilocalories per day in the behavioral treatment of obesity: A randomized trial. *Obesity* 2013;**21:**2481-7.

Nilsen V, Bakke PS, Gallefoss F. Effects of lifestyle intervention in persons at risk for type 2 diabetes mellitus - results from a randomised, controlled trial. *BMC public health* 2011;**11:**893.

O'Neil PM, Miller-Kovach K, Tuerk PW, Becker LE, Wadden TA, Fujioka K, et al. Randomized Controlled Trial of a Nationally Available Weight Control Program Tailored for Adults with Type 2 Diabetes. *Obesity* 2016;**24:**2269-77.

Ostbye T, Stroo M, Brouwer RJ, Peterson BL, Eisenstein EL, Fuemmeler BF, et al. Steps to Health employee weight management randomized Control trial: Short-term follow-up results. *Journal of Occupational and Environmental Medicine* 2015;**57:**188-95.

Pascale RW, Wing RR, Butler BA, Mullen M, Bononi P. Effects of a behavioral weight loss program stressing calorie restriction versus calorie plus fat restriction in obese individuals with NIDDM or a family history of diabetes. *Diabetes Care* 1995;**18:**1241-8.

Pedersen E, Jesudason DR, Clifton PM. High protein weight loss diets in obese subjects with type 2 diabetes mellitus. *Nutrition, Metabolism and Cardiovascular Diseases* 2014;**24:**554-62.

Pekkarinen T, Kaukua J, Mustajoki P. Long-term weight maintenance after a 17-week weight loss intervention with or without a one-year maintenance program: A randomized controlled trial. *Journal of Obesity* 2015;Article 651460

Perri MG, Limacher MC, Durning PE, Janicke DM, Lutes LD, Bobroff LB, et al. Extended-care programs for weight management in rural communities: The Treatment of Obesity in Underserved Rural Settings (TOURS) randomized trial. *Archives of Internal Medicine* 2008;**168:**2347-54.

Perri MG, Limacher MC, Von Castel-Roberts K, Daniels MJ, Durning PE, Janicke DM, et al. Comparative effectiveness of three doses of weight-loss counseling: Two-year findings from the Rural LITE Trial. *Obesity* 2014;**22:**2293-300.

Perri MG, Nezu AM, McKelvey WF, Shermer RL, Renjilian DA, Viegener BJ. Relapse prevention training and problem-solving therapy in the long-term management of obesity. *Journal of Consulting and Clinical Psychology* 2001;**69:**722-6.

For peer review only

Poston WS, Reeves RS, Haddock CK, Stormer S, Balasubramanyam A, Satterwhite O, et al. Weight loss in obese Mexican Americans treated for 1-year with orlistat and lifestyle modification. *International Journal of Obesity & Related Metabolic Disorders* 2003;**27:**1486-93.

Purcell K, Sumithran P, Prendergast LA, Bouniu CJ, Delbridge E, Proietto J. The effect of rate of weight loss on long-term weight management: A randomised controlled trial. *The Lancet Diabetes and Endocrinology* 2014;**2:**954-62.

Rapoport L, Clark M, Wardle J. Evaluation of a modified cognitive-behavioural programme for weight management. *International Journal of Obesity* 2000;**24:**1726-37.

Reichard A, Saunders MD, Saunders RR, Donnelly JE, Lauer E, Sullivan DK, et al. A comparison of two weight management programs for adults with mobility impairments. *Disability and Health Journal* 2015;**8:**61-9.

Reis LO, Favaro WJ, Barreiro GC, Oliveira LC, Chaim EA, Fregonesi A, et al. Erectile dysfunction and hormonal imbalance in morbidly obese male is reversed after gastric bypass surgery: a prospective randomized controlled trial. *International journal of andrology* 2010;**33:**736-44.

Richelsen B, Tonstad S, Rossner S, Toubro S, Niskanen L, Madsbad S, et al. Effect of orlistat on weight regain and cardiovascular risk factors following a very-low-energy diet in abdominally obese patients: A 3-year randomized, placebo-controlled study. *Diabetes Care* 2007;**30:**27-32.

Rock CL, Flatt SW, Pakiz B, Taylor KS, Leone AF, Brelje K, et al. Weight loss, glycemic control, and cardiovascular disease risk factors in response to differential diet composition in a weight loss program in type 2 diabetes: A randomized controlled trial. *Diabetes Care* 2014;**37:**1573-80.

Rossner S, Flaten H. VLCD versus LCD in long-term treatment of obesity. *International Journal of Obesity* 1997;**21:**22-6.

Ryttig KR, Flaten H, Rossner S. Long-term effects of a very low calorie diet (Nutrilett) in obesity treatment. A prospective, randomized, comparison between VLCD and a hypocaloric diet + behavior modification and their combination. *International Journal of Obesity* 1997;**21:**574-9.

Ryttig KR, Rossner S. Weight maintenance after a very low calorie diet (VLCD) weight reduction period and the effects of VLCD supplementation. A prospective, randomized, comparative, controlled long-term trial. *Journal of Internal Medicine* 1995;**238:**299-306.

Schauer PR, Kashyap SR, Wolski K, Brethauer SA, Kirwan JP, Pothier CE, et al. Bariatric surgery versus intensive medical therapy in obese patients with diabetes. *New England Journal of Medicine* 2012;**366:**1567-76.

Shikany JM, Thomas AS, Beasley TM, Lewis CE, Allison DB. Randomized controlled trial of the Medifast 5 & 1 plan for weight loss. *International Journal of Obesity* 2013;**37:**1571-8.

Sjostrom L, Rissanen A, Andersen T, Boldrin M, Golay A, Koppeschaar HPF, et al.

For peer review only

Randomised placebo-controlled trial of orlistat for weight loss and prevention of weight regain in obese patients. *Lancet* 1998;**352:**167-72.

Soenen S, Bonomi AG, Lemmens SGT, Scholte J, Thijssen MAMA, Van Berkum F, et al. Relatively high-protein or 'low-carb' energy-restricted diets for body weight loss and body weight maintenance? *Physiology and Behavior* 2012;**107:**374-80.

Spring B, Duncan JM, Janke EA, Kozak AT, McFadden HG, Demott A, et al. Integrating technology into standard weight loss treatment a randomized controlled trial. *JAMA Internal Medicine* 2013;**173:**105-11.

Stahre L, Hallstrom T. A short-term cognitive group treatment program gives substantial weight reduction up to 18 months from the end of treatment. A randomized controlled trial. *Eating and Weight Disorders* 2005;**10:**51-8.

Stenius-Aarniala B, Poussa T, Kvarnstrom J, Gronlund EL, Ylikahri M, Mustajoki P. Immediate and long term effects of weight reduction in obese people with asthma: Randomised controlled study. *British Medical Journal* 2000;**320:**827-32.

Stern L, Iqbal N, Seshadri P, Chicano KL, Daily DA, McGrory J, et al. The Effects of Low-Carbohydrate versus Conventional Weight Loss Diets in Severely Obese Adults: One-Year Follow-up of a Randomized Trial. *Annals of Internal Medicine* 2004;**140:**778-85+I-27.

Strobl V, Knisel W, Landgraf U, Faller H. A combined planning and telephone aftercare intervention for obese patients: effects on physical activity and body weight after one year. *Journal of rehabilitation medicine* 2013;**45:**198-205.

Swinburn BA, Carey D, Hills AP, Hooper M, Marks S, Proietto J, et al. Effect of orlistat on cardiovascular disease risk in obese adults. *Diabetes Obesity & Metabolism* 2005;**7:**254-62.

Torgerson J, Agren L, Sjostrom L. Effects on body weight of strict or liberal adherence to an initial period of VLCD treatment. A randomised, one-year clinical trial of obese subjects. *International Journal of Obesity* 1999;**23:**190-7.

Torgerson JS, Hauptman J, Boldrin MN, Sjostrom L. XENical in the Prevention of Diabetes in Obese Subjects (XENDOS) Study: A randomized study of orlistat as an adjunct to lifestyle changes for the prevention of type 2 diabetes in obese patients. *Diabetes Care* 2004;**27:**155-61.

For peer review only

Torgerson JS, Lissner L, Lindroos AK, Kruijer H, Sjostrom L. VLCD plus dietary and behavioural support versus support alone in the treatment of severe obesity. A randomised two-year clinical trial. *International Journal of Obesity* 1997;**21:**987-94.

Tsai AG, Wadden TA, Rogers MA, Day SC, Moore RH, Islam BJ. A Primary Care Intervention for Weight Loss: Results of a Randomized Controlled Pilot Study. *Obesity* 2010;**18:**1614-8.

Van Name MA, Camp AW, Magenheimer EA, Li F, Dziura JD, Montosa A, et al. Effective translation of an intensive lifestyle intervention for hispanic women with prediabetes in a community health center setting. *Diabetes Care* 2016;**39:**525-31.

Villareal DT, Chode S, Parimi N, Sinacore DR, Hilton T, Armamento-Villareal R, et al. Weight loss, exercise, or both and physical function in obese older adults. *New England Journal of Medicine* 2011;**364:**1218-29.

Villareal DT, Shah K, Banks MR, Sinacore DR, Klein S. Effect of weight loss and exercise therapy on bone metabolism and mass in obese older adults: A one-year randomized controlled trial. *Journal of Clinical Endocrinology and Metabolism* 2008;**93:**2181-7.

von Gruenigen VE, Courneya KS, Gibbons HE, Kavanagh MB, Waggoner SE, Lerner E. Feasibility and effectiveness of a lifestyle intervention program in obese endometrial cancer patients: A randomized trial. *Gynecologic Oncology* 2008;**109:**19-26.

Wadden TA, Foster GD, Letizia KA. One-year behavioral treatment of obesity: Comparison of moderate and severe caloric restriction and the effects of weight maintenance therapy. *Journal of Consulting and Clinical Psychology* 1994;**62:**165-71.

Wadden TA, Berkowitz RI, Vogt RA, Steen SN, Stunkard AJ, Foster GD. Lifestyle modification in the pharmacologic treatment of obesity: a pilot investigation of a potential primary care approach. *Obesity research* 1997;**5:**218-26.

Wadden TA, Vogt RA, Foster GD, Anderson DA. Exercise and the maintenance of weight loss: 1-year follow-up of a controlled clinical trial. *Journal of Consulting and Clinical Psychology* 1998;**66:**429-33.

Wadden TA, Volger S, Sarwer DB, Vetter ML, Tsai AG, Berkowitz RI, et al. A two-year randomized trial of obesity treatment in primary care practice. *New England Journal of Medicine* 2011;**365:**1969-79.

Weinstock RS, Trief PM, Cibula D, Morin PC, Delahanty LM. Weight Loss Success in Metabolic Syndrome by Telephone Interventions: Results from the SHINE Study. *Journal of General Internal Medicine* 2013;**28:**1620-8.

For peer review only

West DS, DiLillo V, Bursac Z, Gore SA, Greene PG. Motivational interviewing improves weight loss in women with type 2 diabetes. *Diabetes Care* 2007;**30:**1081-7.

West DS, Gorin AA, Subak LL, Foster G, Bragg C, Hecht J, et al. A motivation-focused weight loss maintenance program is an effective alternative to a skill-based approach. *International Journal of Obesity* 2011;**35:**259-69.

Wing RR, Blair E, Marcus M, Epstein LH, Harvey J. Year-long weight loss treatment for obese patients with type II diabetes: Does including an intermittent very-low-calorie diet improve outcome? *American Journal of Medicine* 1994;**97:**354-62.

Wing RR, Marcus MD, Epstein LH, Jawad A. A "family-based" approach to the treatment of obese type II diabetic patients. *Journal of Consulting and Clinical Psychology* 1991;**59:**156-62.

Wing RR, Marcus MD, Salata R, Epstein LH, Miaskiewicz S, Blair EH. Effects of a very-low-calorie diet on long-term glycemic control in obese type 2 diabetic subjects. *Archives of Internal Medicine* 1991;**151:**1334-40.

Wing RR, Venditti E, Jakicic JM, Polley BA, Lang W. Lifestyle intervention in overweight individuals with a family history of diabetes. *Diabetes Care* 1998;**21:**350-9.

Wolf AM, Conaway MR, Crowther JQ, Hazen KY, Nadler JL, Oneida B, et al. Translating lifestyle intervention to practice in obese patients with type 2 diabetes: Improving Control with Activity and Nutrition (ICAN) study. *Diabetes Care* 2004;**27:**1570-6.

Wylie-Rosett J, Swencionis C, Ginsberg M, Cimino C, Wassertheil-Smoller S, Caban A, et al. Computerized weight loss intervention optimizes staff time: the clinical and cost results of a controlled clinical trial conducted in a managed care setting. *Journal of the American Dietetic Association* 2001;**101:**1155-62; quiz 63-64.

Yeh MC, Rodriguez E, Nawaz H, Gonzalez M, Nakamoto D, Katz DL. Technical skills for weight loss: 2-y follow-up results of a randomized trial. *International Journal of Obesity* 2003;**27:**1500-6.

For peer review only

PRISMA 2020 Checklist

Section and Topic	Item #	Checklist item	Location where item is reported
**TITLE**			
Title	1	Identify the report as a systematic review.	Title page (page 1)
**ABSTRACT**			
Abstract	2	See the PRISMA 2020 for Abstracts checklist.	Page 2 (and PRISMA abstracts checklist provided)
**INTRODUCTION**			
Rationale	3	Describe the rationale for the review in the context of existing knowledge.	5 to 6
Objectives	4	Provide an explicit statement of the objective(s) or question(s) the review addresses.	6
**METHODS**			
Eligibility criteria	5	Specify the inclusion and exclusion criteria for the review and how studies were grouped for the syntheses.	6 to 7
Information sources	6	Specify all databases, registers, websites, organisations, reference lists and other sources searched or consulted to identify studies. Specify the date when each source was last searched or consulted.	7 and Appendix 1
Search strategy	7	Present the full search strategies for all databases, registers and websites, including any filters and limits used.	Appendix 1
Selection process	8	Specify the methods used to decide whether a study met the inclusion criteria of the review, including how many reviewers screened each record and each report retrieved, whether they worked independently, and if applicable, details of automation tools used in the process.	7
Data collection process	9	Specify the methods used to collect data from reports, including how many reviewers collected data from each report, whether they worked independently, any processes for obtaining or confirming data from study investigators, and if applicable, details of automation tools used in the process.	7 to 9
Data items	10a	List and define all outcomes for which data were sought. Specify whether all results that were compatible with each outcome domain in each study were sought (e.g. for all measures, time points, analyses), and if not, the methods used to decide which results to collect.	7 to 9
	10b	List and define all other variables for which data were sought (e.g. participant and intervention characteristics, funding sources). Describe any assumptions made about any missing or unclear information.	7 to 9
Study risk of bias assessment	11	Specify the methods used to assess risk of bias in the included studies, including details of the tool(s) used, how many reviewers assessed each study and whether they worked independently, and if applicable, details of automation tools used in the process.	Not applicable as the focus of this analysis is not of the effects of the intervention. For this, the reader is referred to the main REBALANCE paper for details of the risk of bias

For peer review only

PRISMA 2020 Checklist

Section and Topic	Item #	Checklist item	Location where item is reported
			methods and results of the assessment
Effect measures	12	Specify for each outcome the effect measure(s) (e.g. risk ratio, mean difference) used in the synthesis or presentation of results.	7 to 9
Synthesis methods	13a	Describe the processes used to decide which studies were eligible for each synthesis (e.g. tabulating the study intervention characteristics and comparing against the planned groups for each synthesis (item #5)).	8 to 9
	13b	Describe any methods required to prepare the data for presentation or synthesis, such as handling of missing summary statistics, or data conversions.	8 to 9
	13c	Describe any methods used to tabulate or visually display results of individual studies and syntheses.	8 to 9
	13d	Describe any methods used to synthesize results and provide a rationale for the choice(s). If meta-analysis was performed, describe the model(s), method(s) to identify the presence and extent of statistical heterogeneity, and software package(s) used.	8 to 9
	13e	Describe any methods used to explore possible causes of heterogeneity among study results (e.g. subgroup analysis, meta-regression).	NA for descriptive statistics
	13f	Describe any sensitivity analyses conducted to assess robustness of the synthesized results.	NA
Reporting bias assessment	14	Describe any methods used to assess risk of bias due to missing results in a synthesis (arising from reporting biases).	NA
Certainty assessment	15	Describe any methods used to assess certainty (or confidence) in the body of evidence for an outcome.	NA for descriptive statistics
**RESULTS**			
Study selection	16a	Describe the results of the search and selection process, from the number of records identified in the search to the number of studies included in the review, ideally using a flow diagram.	10 and Appendix 2
	16b	Cite studies that might appear to meet the inclusion criteria, but which were excluded, and explain why they were excluded.	No studies were excluded. The dataset comprised 131 RCTs included in a systematic review of weight management interventions for adults with BMI ≥35kg/m2, as part of the REBALANCE project (NIHR

For peer review only

PRISMA 2020 Checklist

Section and Topic	Item #	Checklist item	Location where item is reported
			HTA15/09/04). All 131 RCTs were included in this analysis.
Study characteristics	17	Cite each included study and present its characteristics.	10 and Appendix 3
Risk of bias in studies	18	Present assessments of risk of bias for each included study.	NA (see response to item 11)
Results of individual studies	19	For all outcomes, present, for each study: (a) summary statistics for each group (where appropriate) and (b) an effect estimate and its precision (e.g. confidence/credible interval), ideally using structured tables or plots.	14, 16, 18
Results of syntheses	20a	For each synthesis, briefly summarise the characteristics and risk of bias among contributing studies.	12, 15, 17
	20b	Present results of all statistical syntheses conducted. If meta-analysis was done, present for each the summary estimate and its precision (e.g. confidence/credible interval) and measures of statistical heterogeneity. If comparing groups, describe the direction of the effect.	10 to 18
	20c	Present results of all investigations of possible causes of heterogeneity among study results.	NA
	20d	Present results of all sensitivity analyses conducted to assess the robustness of the synthesized results.	NA
Reporting biases	21	Present assessments of risk of bias due to missing results (arising from reporting biases) for each synthesis assessed.	NA
Certainty of evidence	22	Present assessments of certainty (or confidence) in the body of evidence for each outcome assessed.	NA
**DISCUSSION**			
Discussion	23a	Provide a general interpretation of the results in the context of other evidence.	19
	23b	Discuss any limitations of the evidence included in the review.	20
	23c	Discuss any limitations of the review processes used.	23
	23d	Discuss implications of the results for practice, policy, and future research.	23, 24
**OTHER INFORMATION**			
Registration and protocol	24a	Provide registration information for the review, including register name and registration number, or state that the review was not registered.	3
	24b	Indicate where the review protocol can be accessed, or state that a protocol was not prepared.	7
	24c	Describe and explain any amendments to information provided at registration or in the protocol.	NA
Support	25	Describe sources of financial or non-financial support for the review, and the role of the funders or sponsors in the review.	39-40
Competing interests	26	Declare any competing interests of review authors.	39
Availability of data, code and other materials	27	Report which of the following are publicly available and where they can be found: template data collection forms; data extracted from included studies; data used for all analyses; analytic code; any other materials used in the review.	40

For peer review only - http://bmjopen.bmj.com/site/about/guidelines.xhtml

From: Page MJ, McKenzie JE, Bossuyt PM, Boutron I, Hoffmann TC, Mulrow CD, et al. The PRISMA 2020 statement: an updated guideline for reporting systematic reviews. BMJ 2021;372:n71. doi: 10.1136/bmj.n71

For peer review only

PRISMA 2020 Checklist

For more information, visit: http://www.prisma-statement.org/

For peer review only

PRISMA 2020 for Abstracts Checklist

Section and Topic	Item #	Checklist item	Reported (Yes/No)
**TITLE**			
Title	1	Identify the report as a systematic review.	Y
**BACKGROUND**			
Objectives	2	Provide an explicit statement of the main objective(s) or question(s) the review addresses.	Y
**METHODS**			
Eligibility criteria	3	Specify the inclusion and exclusion criteria for the review.	Y
Information sources	4	Specify the information sources (e.g. databases, registers) used to identify studies and the date when each was last searched.	Y
Risk of bias	5	Specify the methods used to assess risk of bias in the included studies.	Not applicable as the focus of this analysis is not of the effects of the intervention. For this, the reader is referred to the main REBALANCE paper for details of the risk of bias methods and results of the risk of bias assessment.
Synthesis of results	6	Specify the methods used to present and synthesise results.	Y
**RESULTS**			

For peer review only

PRISMA 2020 for Abstracts Checklist

Section and Topic	Item #	Checklist item	Reported (Yes/No)
Included studies	7	Give the total number of included studies and participants and summarise relevant characteristics of studies.	Y
Synthesis of results	8	Present results for main outcomes, preferably indicating the number of included studies and participants for each. If meta-analysis was done, report the summary estimate and confidence/credible interval. If comparing groups, indicate the direction of the effect (i.e. which group is favoured).	Y
**DISCUSSION**			
Limitations of evidence	9	Provide a brief summary of the limitations of the evidence included in the review (e.g. study risk of bias, inconsistency and imprecision).	Y
Interpretation	10	Provide a general interpretation of the results and important implications.	Y
**OTHER**			
Funding	11	Specify the primary source of funding for the review.	Y
Registration	12	Provide the register name and registration number.	Y

From: Page MJ, McKenzie JE, Bossuyt PM, Boutron I, Hoffmann TC, Mulrow CD, et al. The PRISMA 2020 statement: an updated guideline for reporting systematic reviews. BMJ 2021;372:n71. doi: 10.1136/bmj.n71

For more information, visit: http://www.prisma-statement.org/

For peer review only
